# A preliminary study of the probitive value of personality assessment in medical school admissions within the United States

**DOI:** 10.1186/s12909-022-03901-x

**Published:** 2022-12-23

**Authors:** A. Peter Eveland, Sabrina R. Wilhelm, Stephanie Wong, Lissett G. Prado, Sanford H. Barsky

**Affiliations:** 1 Department of Medical Education, Cancer Center and Institute for Personalized Medicine, California University of Science and Medicine (CUSM), 1501 Violet Street, Colton, CA 92324 USA; 2 Department of Pathology, Anatomy and Cell Biology and the Clinical and Translational Research Center of Excellence, Meharry Medical College, 1005 Dr. D.B. Todd Jr. Boulevard, Nashville, TN 37208 USA

**Keywords:** Medical school admissions, Medical school interviews, Personality assessments, Neo personality inventory-revised test

## Abstract

**Background:**

Allopathic medicine faces a daunting challenge of selecting the best applicants because of the very high applicant / matriculant ratio. The quality of graduates ultimately reflects the quality of medical practice. Alarming recent trends in physician burnout, misconduct and suicide raise questions of whether we are selecting the right candidates. The United States (US) lags far behind the United Kingdom (UK) and Europe in the study of non-cognitive tests in medical school admissions. Although more recently, medical schools in both the UK, Europe and the US have begun to use situational judgement tests such as the Computer-Based Assessment for Sampling Personal Characteristics (CASPer) and the situational judgement test (SJT), recently developed by the Association of American Medical Colleges (AAMC) and that these tests are, in a sense non-cognitive in nature, direct personality tests per se have not been utilized. We have historically used, in the admissions process within the US, knowledge, reasoning and exam performance, all of which are largely influenced by intelligence and also improved with practice. Personality, though also undoubtedly influenced by intelligence, is fundamentally different and subject to different kinds of measurements.

**Methods:**

A popular personality measurement used over the past two decades within the US in business and industry, but not medical school has been the Neo Personality Inventory – Revised (NEO-PI-R) Test. This test has not been utilized regularly in allopathic medicine probably because of the paucity of exploratory retrospective and validating prospective studies. The hypothesis which we tested was whether NEO-PI-R traits exhibited consistency between two institutions and whether their measurements showed probative value in predicting academic performance.

**Results:**

Our retrospective findings indicated both interinstitutional consistencies and both positive and negative predictive values for certain traits whose correlative strengths exceeded traditional premed metrics: medical college admission test (MCAT) scores, grade point average (GPA), etc. for early academic performance.

**Conclusions:**

Our exploratory studies should catalyze larger and more detailed confirmatory studies designed to validate the importance of personality traits not only in predicting early medical school performance but also later performance in one’s overall medical career.

**Supplementary Information:**

The online version contains supplementary material available at 10.1186/s12909-022-03901-x.

## Background

Intelligence and personality are two indelible components of the human condition. Cognitive skills, knowledge, reasoning and exam performance, on the other hand, can be acquired and improved through practice [1]. We nearly exclusively use the latter in the medical school admission process in the US, and largely ignore personality, at least by formal assessment. Alarming recent trends in physician burnout, misconduct and suicide raise additional questions of whether we are selecting the right candidates in our medical school admissions process. It is not entirely clear why in the US we persist in mainly using premed cognitive assessments in selecting whom to accept to medical school. Not only have we continually ignored non-cognitive assessments in the admissions process but we have not even conducted retrospective or prospective studies examining their potential value in predicting early medical school performance or later performance in one’s overall medical career. This dearth of US studies stands in contrast to UK and European studies which consist of a number of large cohort studies examining non-cognitive testing which include both modifiable as well as non-modifiable personality traits and their predictive values during and at completion of medical school [2–11].

Although more recently, medical schools in both the UK, Europe and the US have begun to use situational judgement tests such as CASPer and SJT, recently developed by the AAMC and that these tests are, in a sense non-cognitive in nature [12, 13], direct personality tests per se have not been utilized. An increasingly popular formal measurement of personality, however, which has evolved over the past two decades, is the NEO-PI-R Test, a measurement of five major domains of personality as well as six facets that define each of the domains (Table 1) [14, 15]. The NEO-PI-R is a psychological personality inventory consisting of the Five Factor Domain (Model): Extraversion, Agreeableness, Conscientiousness, Neuroticism and Openness to Experience. The test also measures six subordinate dimensions, known as ‘facets’ of each of the five factor model personality domains. The NEO-PI-R consists of 240 items of descriptions of behavior answered on a five point scale, ranging from “strongly disagree” to strongly agree” [14]. The test is available both online and in paper form and has been used widely in the evaluation of employee applications in business, industry, law enforcement and selectively high pressured occupations, e.g., air traffic controllers [16, 17]. The test has not formally or officially been used in the allopathic medical school admissions process in the United States for reasons that are not totally clear. Perhaps one reason it has not been used is that there have been a paucity of exploratory retrospective and validating prospective studies examining the value of formal personality assessment in the medical school setting.

**Table 1:** The Revised NEO Personality Inventory Test (NEO PI-R)

**(N) Neuroticism**
(N1) Anxiety
(N2) Hostility
(N3) Depression
(N4) Self-Consciousness
(N5) Impulsiveness
(N6) Vulnerability to Stress
**(E) Extraversion**
(E1) Warmth
(E2) Gregariousness
(E3) Assertiveness
(E4) Activity
(E5) Excitement Seeking
(E6) Positive Emotion
**(O) Openness to experience**
(O1) Fantasy
(O2) Aesthetics
(O3) Feelings
(O4) Actions
(O5) Ideas
(O6) Values
**(A) Agreeableness**
(A1) Trust
(A2) Straightforwardness
(A3) Altruism
(A4) Compliance
(A5) Modesty
(A6) Tendermindedness
**(C) Conscientiousness**
(C1) Competence
(C2) Order
(C3) Dutifulness
(C4) Achievement Striving
(C5) Self-Discipline
(C6) Deliberation

Even though the NEO-PI-R Test has been used sparingly in the US, there have, in fact, been a number of studies specifically examining personality and medical school performance with the majority of studies occurring outside of the United States [18–31]. The results of these studies have been mixed with some showing added predictive value of certain personality traits over cognitive tests and others showing no added value. The vast majority of these studies did not use the NEO-PI-R instrument specifically as the measurement of personality. Most of the studies used subjective measurements of performance in the clinical years involving patient interactions [32, 33]. However none of these studies used the NEO-PI-R either singularly or in combination with cognitive premed measurements to grant or deny admission to medical school.

It can be argued however that since the medical school admissions process uses either in-person or virtual interviews [34], that some aspects of applicant personality invariably surface during the interview process and may influence decisions of acceptance [35, 36]. However that is different than a formal, systematic, objective, quantitative and reproducible measurement of personality as can be offered by the NEO-PI-R test. Overall, there has been, in fact, only a paucity of studies examining personality traits of medical applicants and matriculants [37]. Exploratory retrospective and confirmatory prospective studies of the NEO-PI-R are first needed to justify its routine use in the medical school admissions process. For these studies to be valid, the NEO-PI-R test must be separately administered to all applicants granted an interview but must not at all be used, at least initially, to influence the admissions process and interviewees must not be told whether the test will influence or not influence the decision process. This is exactly what we did in the present study. The hypothesis which we tested was whether personality traits measured by NEO-PI-R are consistent between two institutions and whether they have value in predicting academic success as well as failure, greater than traditional premed metrics (MCAT, GPA, etc.).

## Methods

This study was conducted under strict Family Educational Rights and Privacy Act (FERPA) guidelines. All data had been collected as part of the routine admissions process and subjects de-identified. The present study was approved by the California University of Science and Medicine (CUSM)’s institutional review board (IRB) (HS-2020–04). We had previously collected 2 year’s worth of matriculant data from Mercer University School of Medicine (MUSM) under an approved IRB (H0312123). All raw data analyzed in the study is provided as Supplementary files (Additional files 1, 2, 3, 4 and 5).

This study was conducted blindly. The individuals at both institutions who administered the NEO-PI-R to the interviewees and recorded the results did not participate in any other aspects of the medical school interview or admissions process, did not interact with members of the Admissions Committee in any way nor participate in the deliberations or decisions of the Admissions Committee. Numerical values of NEO traits and subtraits from all interviewees from the classes of 2022 and 2023 at CUSM and all students from the classes of 2006 and 2007 at MUSM were descriptively summarized using means, standard deviations, minimums, maximums, ranges, and variances. The CUSM class composition and demographics for 2022 and 2023 is depicted (Table 2). Comparisons of means between NEO personality traits of CUSM and MUSM students were conducted using one-way analysis of variance (ANOVA) to determine any statistically significant differences. An alpha value of 0.05 was considered significant.

**Table 2:** Demographics of NEO-PI-R Test

**Demographics**	**Class of 2022**		**Class of 2023**	
	**N**	**%**	**N**	**%**
**Sex**				
Female	147	49%	195	44%
Male	153	51%	248	56%
**Ethnicity**				
Hispanic	54	18%	93	21%
Asian	96	32%	182	41%
Caucasian	129	43%	133	30%
African American	3	1%	9	2%
Other	18	6%	26	6%

NEO traits between those with good vs poor performance at MUSM were compared using an independent sample t-test. Poor performance had three subcategories: repeating a single course, repeating multiple courses or dropping out of school. NEO traits of students with good performance were compared to NEO traits of students with poor performance.

Comparisons between accepted students and rejected students at CUSM were assessed by conducting an independent sample t-test for NEO traits of accepted students and NEO traits of rejected students for both classes of 2022 and 2023, and each year individually.

NEO traits of accepted and rejected students from CUSM and MUSM were subsequently compared using independent sample t-tests in the following categories: (1) MUSM vs CUSM All Accepted, (2) MUSM vs CUSM All Rejected, (3) MUSM vs CUSM Year 1 Accepted, (4) MUSM vs CUSM Year 1 Rejected, (5) MUSM vs CUSM Year 2 Accepted, (6) MUSM vs CUSM Year 2 Rejected.

Correlations between different NEO traits in CUSM students were calculated using a 2-tailed Pearson bivariate correlation and charted as a matrix. An alpha value of 0.05 was considered significant. Correlations between NEO traits in CUSM students and select examination scores were similarly calculated. Correlations between NEO traits in CUSM students and typical premedical admissions metrics as well as medical school performance metrics were also calculated.

Differences in NEO traits between male and female accepted and rejected applicants were compared using independent sample t-tests for both CUSM classes of 2022 and 2023.

A more detailed enumeration of the tests and comparisons that were conducted is provided (Table 3).

**Table 3:** Summary of analyses

1. (2-27-2020) CUSM descriptive statistics (all applicants interviewed)
1. N, range, minimum, maximum, mean, standard error of the mean, standard deviation, and variance calculated for each NEO trait and subcategories
2. (2-27-2020) Mercer descriptive statistics (class of 2006 and 2007)
1. N, range, minimum, maximum, mean, standard error of the mean, standard deviation, and variance calculated for each NEO trait and subcategories
3. (2-27-2020) CUSM vs Mercer Comparisons (One way ANOVA)
1. One way ANOVA was used to compare the means of each trait to see if there was any statistically significant difference in traits between CUSM and Mercer students
2. Reported statistics: sum of squares, df, mean square, F, significance (both between groups and within groups)
3. An alpha value of 0.05 was considered significant
1. A (agreeableness), A6 (tender-mindedness) were stat sig
4. (3-2-2020) Mercer performance data
1. All NEO traits and subtraits compared using an independent sample t-test for equality of means
2. Mercer performance data was broadly divided into the following:
1. Good vs poor performance (latter group included students who: repeated a year, took a LOA, or quit)
1. Only 14 in the poor performance group (not enough power?)
2. Group 0 = Good performance (*N* = 102)
3. Group 1 = Bad performance (*N* = 14)
4. None of the traits had significant differences
2. Good vs LOA or quit t-test
1. Group 0 = Good performance (*N* = 102)
2. Group 2 = LOA + quit (*N* = 5)
3. E5 (excitement-seeking) stat sig
3. LOA vs quit t-test
1. Group 2 = LOA (*N* = 4)
2. Group 3 = quit (*N* = 1)
3. E2 (gregariousness) stat sig
4. Repeat year vs LOA t-test
1. Group 1 = Repeat year (*N* = 9)
2. Group 2 = LOA (*N* = 4)
3. No stat sig
5. Repeat year vs Quit t-test
1. Group 1 = Repeat year (*N* = 9)
2. Group 3 = quit (*N* = 1)
3. E5 (excitement-seeking) stat sig
5. (3-15-2020) CUSM Accepted vs Rejected Analyses (2022 and 2023)
1. All NEO traits and subtraits compared using an independent sample t-test for equality of means
2. CUSM Accepted Year 1 and 2
1. 1AY = ℅ 2022 accepted (*N* = 65)
2. 2AY = ℅ 2023 accepted (*N* = 98)
3. N, O, N3, N4, E6, O2, A1, A6 stat sig
3. CUSM All Accepted vs Rejected
1. AY = All accepted *N* = 163
2. RY = All rejected *N* = 811
3. N, N1, N2, N3, N4, N5, N6 stat sig
4. CUSM Year 1 Accepted vs Rejected
1. 1AY = ℅ 2022 accepted (*N* = 65)
2. 1RY = ℅ 2022 rejected (*N* = 361)
3. O6 stat sig
5. CUSM Year 2 Accepted vs Rejected
1. 2AY = ℅ 2023 accepted (*N* = 98)
2. 2RY = ℅ 2023 rejected (*N* = 450)
3. N, N1, N2, N3, N4, N5, N6 stat sig
6. (3-15-2020) Mercer vs CUSM Accept vs Reject Comparisons
1. All NEO traits and subtraits compared using an independent sample t-test for equality of means
2. Mercer vs CUSM All Accepted
1. MERCER *N* = 116
2. CUSM *N* = 163
3. N, O, A, C, N1-N6, E1, E2, E4, E6, O2, O4, O5, O6, A1-A6, C1, C3-C6 stat sig
3. Mercer vs CUSM All Rejected
1. MERCER *N* = 116
2. CUSMREJ *N* = 811
3. NOAC, N1-6, E1, E2, E4, E6, O2,O4-6, A1-6, C1-6 stat sig
4. Mercer vs CUSM Year 1 Accepted
1. MERCER *N* = 116
2. 1AY = CUSM Year 1 Accepted *N* = 65
3. NOA, N1-6, E1, E4, O2, O4-6,A1-6, C1, C3-6 stat sig
5. Mercer vs CUSM Year 1 Rejected
1. MERCER *N* = 116
2. 1RY = CUSM Year 1 Rejected *N* = 361
3. NOAC, N1-6, E1, E2, E4, E6, O2, O4-6, A1-6, C1, C3-6 stat sig
6. Mercer vs CUSM Year 2 Accepted
1. MERCER *N* = 116
2. 2AY = CUSM Year 2 Accepted *N* = 98
3. NOAC, N1-6, E1, E2, E6, O2, O4-6, A1-6, C1-6
7. Mercer vs CUSM Year 2 Rejected
1. MERCER *N* = 116
2. 2RY = CUSM Year 2 Rejected *N* = 450
3. NOAC, N1-6, E1-2, E4, E6, O2, O4-6, A1-6, C1-6
7. (3-22-2020) CUSM NEO trait correlations
1. Pearson Bivariate Correlations (2-tailed)
1. An alpha value of 0.05 was considered significant. An alpha value of 0.01 was considered significantly higher.
2. All NEO traits and subtraits correlation calculated and charted as a matrix
8. (3-22-2020) CUSM Class Rank Trait Correlations 2022 and 2023
1. Pearson Bivariate Correlations (2-tailed)
2. Rank correlated with all NEO traits and subtraits for 2022 and 2023 and charted as a matrix
3. Rank values:
1. 1 = Bottom 10%
2. 2 = Middle 80%
3. 3 = Top 10%
9. (4-24-2020) CUSM Premed vs NEO on performance 2022 and 2023
1. Pearson Bivariate Correlations (2-tailed)
1. An alpha value of 0.05 was considered significant. An alpha value of 0.01 was considered significantly higher.
2. One version truncated that includes correlations between premed metrics (MCAT, CGPA, BCPM) and medical school performance metrics averaged (NBME AVG, MCQ AVG, LAB AVG, CP AVG, IRAT AVG, OSCE AVG, CRS AVG)
3. Truncated correlations between NEO traits and subtraits with averaged med school performance metrics
4. Complete version that correlates premed or NEO traits to medical school performance in each individual class
10. (5-22-2020) M vs F Accepted vs Rejected
1. All NEO traits and subtraits compared using an independent sample t-test for equality of means
1. Year 1 = Class of 2022; Year 2 = Class of 2023
2. Year 1 Accepted M vs F
3. Year 1 Accepted vs Rejected
4. Year 1 Rejected M vs F
5. Year 1 and 2 Accepted M vs F
6. Year 1 and 2 Accepted vs Rejected
7. Year 1 and 2 Rejected M vs F
8. Year 2 Accepted M vs F
9. Year 2 Accepted vs Rejected
10. Year 2 Rejected M vs F

## Results

The hypothesis which we tested was whether personality traits as measured by the NEO-PI-R Test have predictive value in early medical school performance and whether this predictive value was stronger than traditional premed metrics (MCAT, GPA, etc.). Obviously, if support for this hypothesis could be obtained from this study, it would argue possibly for an expanded role of the NEO-PI-R Test in the medical school admissions process or at least for additional confirmatory retrospective and validatory prospective studies.

At MUSM, the Admissions Committee did not formally use the NEO-PI-R test to evaluate prospective applicants and were completely blinded to the NEO-PI-R Test results. Therefore, any correlations between personality scores and academic performance were made on an unselected and therefore seemingly unbiased population, at least on the surface. In the present study we re-analyzed the MUSM raw data. We also made comparisons between the MUSM and CUSM data.

The present study also examined 2 years of CUSM applicant and matriculant data for NEO-PI-R, premedical parameters, demographic data and medical school performance data for potential predictive value of the NEO-PI-R vs traditional premed parameters.

Even though the MUSM data and the CUSM data were derived from different populations of medical school applicants, approximately 15 years apart, with different demographic features, (eg., the male / female ratio was much higher at MUSM), from different schools with different admission criteria, and from different geographic areas of the United States, the NEO-PI-R was remarkably consistent in the personality mean scores and ranges between the two groups of students. 29 of 30 facets of personality showed no differences in score distribution between the populations (*p* = 0.87; *p* = 0.78). The single facet showing a difference between the two populations was (A6) Tender-Mindedness (*p* = 0.007). This facet accounted for a difference in its member domain (A) Agreeableness (*p* = 0.034). The fact that 29/30 personality facets showed no differences between the MUSM and CUSM student populations demonstrated the remarkable consistency of the NEO-PI-R. This consistency spanned decades, schools, demographics and geographies.

Re-analysis of the MUSM data revealed a number of interesting findings. For one there were significant differences in one major personality domain as well as many of its facets between males *v* females. The one major domain which showed differences was (C) Conscientiousness with females scoring higher (*p* = 0.012). Females also scored higher in two of its facets: (C2) Order (*p* = 0.026) and (C6) Deliberation (*p* = 0.02). Within the domain of (A) Agreeableness, the facet (A4) Compliance showed higher scores in males (*p* = 0.032).

A number of personality domains and facets correlated with either academic success or failure in both males and females. Academic success was defined by separate and cumulative course performance and academic failure was defined as having to repeat a single course or multiple courses or dropping out of school. The predictive values of these personality domains and facets were compared to the predictive values of traditional premed metrics like MCAT verbal reasoning (VR), MCAT physical sciences (PS): chemistry, physics and MCAT biological sciences (BS): biology, biochemistry, genetics, physiology, molecular biology, microbiology, evolution, organic chemistry. At the time of the MUSM study, the MCAT was divided into MCAT VR, MCAT PS and MCAT BS. The MCAT BS scores positively correlated with 7 different course performances (*p* = 0.05; Pearson 0.6) and the MCAT PS positively correlated with 2 course performances (*p* = 0.05; Pearson 0.6) whereas MCAT VR negatively correlated with 4 course performances (*p* = 0.01; Pearson -0.7). However, none of the MCAT scores correlated with academic failure.

A number of personality domains and facets also correlated positively (significant and positive Pearson coefficients) with course performances. Most of these fell within the (C) Conscientiousness domain which included (C3) Dutifulness, 5 courses (*p* = 0.03; Pearson 0.8); (C4) Achievement Striving, 4 courses (*p* = 0.04; Pearson 0.7); and (C5) Self-Discipline, 7 courses (*p* = 0.02; Pearson 0.9). Collectively, the facets within the (C) Conscientiousness domain correlated with academic success in more courses than the MCAT BS and MCAT PS scores.

However, the most striking finding in the MUSM data was the negative correlations (significant and negative Pearson coefficients) with academic failure. The personality domains and facets which provided strong negative correlations with academic failure (repeating a single course, multiple courses or dropping out of school) fell mainly within the (N) Neuroticism domain including facets (N2) Angry Hostility (*p* = 0.05; Pearson -0.7) and (N3) Depression (*p* = 0.05; Pearson -0.7) and the (O) Openness to Experience domain which included facets (O2) Aesthetics (*p* = 0.05; Pearson -0.7) and (O3) Feelings (*p* = 0.05; Pearson -0.7). The facets within the (O) Openness to Experience domain negatively correlated with repeating not just one but multiple courses (*p* = 0.028; Pearson -0.9). Select personality domains and facets therefore potentially add value to the admissions process as a negative predictor of academic failure.

Similarly to the MUSM students whose admissions to medical school were not at all based on the NEO-PI-R test, CUSM did not use the NEO-PI-R test to formally influence admissions. In the first class which was admitted (the class of 2022), 29 of 30 facets of personality predictably showed no differences in score distribution between the accepted vs rejected applicants (*p* = 0.250). In the second class which was admitted (the class of 2023), there were differences in only 1 domain: (N) Neuroticism. In fact, all of the facets within this domain showed differences between accepted vs. rejected applicants (*p* = 0.02). Although the NEO-PI-R test was not formally used as an Admissions Criteria and whose results were not made available to the Admissions Committee, it was entirely possible that the interviewers were sensitive to neurotic personality traits of certain applicants that negatively impacted their decisions on acceptance. It would seem then from this observation that this domain may have factored into the admission decision.

Analysis of the CUSM data revealed both similarities and differences compared to the MUSM data. The personality profiles of males vs females were again different but mainly fell in facets within the (E) Extraversion, (O) Openness to Experience and (A) Agreeableness domains (*p* = 0.02). CUSM accepted approximately equal number of males and female students whereas MUSM accepted only a limited number of female students at that time. The difference in male / female ratio between the two classes could explain the discrepancy in the differing personality facets.

Since there is currently more of an emphasis on evaluating medical school student performance to comply with the rigors of the Liaison Committee on Medical Education (LCME) accreditation process than there was 15 years ago, CUSM used a number of performance metrics that were not available at MUSM which included Multiple Choice Questions (MCQs), National Board of Medical Examiners (NBME) (both raw and scaled), Laboratory, Case Presentation, Individual Reading Assurance Test (iRAT), Objective Structured Clinical Examination (OSCE), Course Final Grade (derived from a composite of measurements depicted below) and Overall Averages (Table 4).

**Table 4:** Enumeration of various assessments

1. Assessment and Course Grading. Assessments are outcomes based so that learners and faculty can evaluate progress in the development of competencies expected for the course. Some scores will be earned individually, some scores will be earned as a team. It is the student’s responsibility to read the Student Assessment Handbook and familiarize themselves with the policies, regulations and procedures regarding assessments and evaluations.			
	**When**	**%**	**No lab**
iRAT/tRAT quiz (*participation*)	Start of Flipped Class	5	10
Student Case Presentations	Fridays	10	10
Lab Practical OSPE	End of course	15	
End of course MCQ	End of course	40	45
NBME MCQ Exam (internally scaled score)	End of course	20	25
Peer evaluation (3%)	End of course	10	10
Attendance (3%)			
Completion of course/faculty evaluations (4%)			
2. iRAT/tRAT quizzes. These questions (in USMLE Step 1 format) are based on the pre-assigned material for flipped classroom sessions; two questions are set from each session. The scores from the RAT quizzes during the course provide students with an indication of how they are progressing in the course (and serve as feedback) as well as identifying topics and concepts that may require additional study. The participation earned in the quizzes contribute to the final grade in the course.			
3. Multiple Choice Exam (MCQ). This examination is administered during the exam week at the end of the course. The number of questions depends on the duration of the course. Questions are in USMLE Step 1 format. The exam covers all material presented during the course, and the make-up of the exam reflects the weight that each discipline contributed to the course.			
4. NBME Standardized tests. There is an NBME test during the exam week. The test contains 75 questions and examines material learned during the course. The standard of the questions in the exam is based on USMLE Step 1 and provides students an opportunity to assess their preparation for the Step 1 exam. Performance in the NBME exam is internally scaled to account for variability in exam difficulty (NBME exam difficulty does vary between test versions).			
5. Practice Lab practical exam.			
a. Mock/Practice Lab Practical Examination: There is an optional laboratory practical examination during the last week of the course that covers all laboratory material studied (anatomy, physiology, histology, pathology, microbiology).			
b. End-of-course Lab Practical Examination: There is a lab practical examination (objective structured lab exam OSPE) at the end of the course that covers all the laboratory material learned during the course (anatomy, physiology, histology, pathology, microbiology).			
6. Clinical case presentation. Students are assessed by instructors who facilitate the clinical presentations, using rubric-based evaluation forms. The scores from certain presentations contribute towards the final course grade. The scores from certain presentations contribute towards the final course grade.			
7. Other Assessments.			
a. Peer Evaluation. Students evaluate their team members using a peer-to-peer evaluation form. Peer evaluation occurs twice during the course: the first evaluation during the course, the second occurs during the last week of the course.			
b. Attendance. One element of the peer evaluation is to collect information relating to attendance during the course. This contributes to the final grade.			
c. Course/Faculty Evaluation. Students are required to provide feedback regarding the course and faculty teaching. Students will receive and must complete a survey evaluating the course and faculty teaching in the course. Non-compliance reduces the grade assigned to this category.			
In addition independent of overall Course grade is a measurement termed **OSCE** (also mentioned in the list of metrics provided previously): Objective Structured Clinical Exam. This is where standardized patients are used to assess clinical encounters. This is included as an additional independent.			

The Course Final Grade (Raw Score) was derived from a composite of the detailed measurements as depicted (Table 4). In addition, other premedical metrics that were available included overall MCAT, overall GPA and Biology, Chemistry, Physics, Mathematics (BCPM) grade point average. Presently, only an overall MCAT score was available because the MCAT was no longer broken into MCAT VR, MCAT PS and MCAT BS as it was for the MUSM data.

At CUSM, presently, academic failure was defined as the need to repeat a course but since no CUSM students to date, however, have been required to repeat a course due to students’ 100% successful attempts at remediation, academic failure per se could not be correlated with NEO-PI-R measurements, Academic performance (success or lack thereof) based on various assessments including examination scores (Table 3) could be measured and was used in this study.

With traditional premed metrics, MCAT scores surprisingly did not significantly correlate with any of the above-mentioned assessments (*p* = 0.5). However, BCPM significantly correlated with 3 of the assessments (*p* = 0.01; Pearson 0.7) and was therefore the best of the objective metrics.

However, the most striking finding discovered was the very strong negative correlations (significant and negative Pearson coefficients) with academic performance by certain personality domains and facets. The personality domains and facets which provided strong negative correlations with academic performance fell mainly within the (N) Neuroticism domain including facets (N2) Angry Hostility, (N3) Depression, (N5) Impulsiveness and (N6) Vulnerability (all, *p* = 0.02; Pearson -0.8). These facets negatively correlated with as many as 4 of the assessments, which were more assessments than those that correlated with the BCPM. Interestingly, the (N) Neuroticism domain including facets (N2) Anger Hostility and (N3) Depression were also the same personality domain and facets that predicted academic failure at MUSM.

We need to comment further on our data generated by our multiple analyses of overall relatively small sample size in this preliminary study.

Firstly, given that we conducted multiple Pearson’s correlation tests on the data and which therefore were subject to type 1 error, we needed to adjust for potential false positives using statistical techniques developed in the past [38]. This would be considered standard practice in the personality literature where multiple hypotheses are tested on the same underlying data. In response to this issue, we applied the specific Bonferroni correction to our data [39]. While we still can not completely exclude a type I error because of our relatively small sample size, for some of the personality traits vs academic performance, the *p* values still approached significance even when applying the Bonferroni correction.

Secondly, it could be argued that we should interpret our significant correlations in a more direct manner to gauge the magnitude of the exact association between personality trait scores and medical school performance. For instance, when discussing the correlations between personality traits and academic performance, it would be helpful if there was a clearer explanation of what a correlation value of Pearson = 0.8 might mean. For instance, for every one unit increase in a personality XXX there was a YYY increase / decrease in corresponding student’s academic performance. Personality traits usually demonstrate correlations at best in the ± 0.10 to ± 0.30 range with most outcomes. This was the case in our study for the majority of personality traits measured by the NEO-PI-R. However certain specific NEO-PI-R traits stood out for both positive and negative correlations with academic performance with Pearsons ± 0.7 or greater and it is these specific traits and correlations that we are highlighting. Although we completely agree that it would be desirable to more precisely define the meaning of correlation, our overall analysis of student performance was not based on linear class rank but a threshold (passing or failing a course) and therefore given these measurements, a quantitative linear correlation of Pearson units with quantitative performance could not be made.

Thirdly, it might be argued that we should justify choosing a minimum effect size of interest [40] given the abundant correlations that were found in the dataset, ie., what is the theoretically significant minimum effect size (e.g., the lowest “significant” Pearson value) that is large enough to warrant interpretation, beyond just the alpha = 0.05). One way to do this would be to outline the average correlations between other student metrics and medical school performance (eg., what is the correlation between intelligence and medical school performance scores?) so that one could gauge the relative importance of personality trait measures. Hypothetically, we could use Ordinary Least Squares (OLS) regression analysis to compare the additional variance in medical school performance explained by the addition of personality variables to traditional metrics / other sources of signal. But again the relatively small number of cases in this first preliminary study does not support any strong conclusions regarding a theoretical significant minimum effect size based on the Pearson beyond just the alpha = 0.05 and further limits choosing a minimum effect size of interest despite the abundant correlations that were found in the dataset. Because of the small size of our study we therefore could not use OLS regression analysis. Because of potential Type I errors, we rather prefered to focus on Pearson values significantly higher than the alpha = 0.05 threshold and that is exactly what we did.

Fourthly, we used ANOVA because in this preliminary study, the data obtained was single measurement data obtained at one time point as opposed to repeated measurements over time. Although multi-level models (MLMs), also known as linear mixed models, hierarchical linear models or mixed-effect models, have become increasingly popular for analyzing data with repeated measurements, our present study was not ripe for this approach. As we collect more longitudinal data of student academic performance over time, we will use analysis with MLMs.

Finally, we also ran a detailed Statistical Product and Service Solution (SPSS) analysis of the data (Additional file 5) which displayed details of the correlations and intercorrelations showing sample sizes for each correlation. Our data show that our personality traits usually demonstrated weak to moderate correlations to performance outcomes in the 0.10 to 0.30 range. However certain selective traits, eg., “Openness to Experience” and repeating multiple courses did show very strong negative correlations in the Pearson (-0.7 – -0.9 range) but with a *p* value of only 0.028. A correlation of 0.90 should have a very small *p*-value unless the sample size was small. This was indeed the case as this specific correlation consisted of only 9 subjects. However our overall study is not underpowered. Firstly, the study is only a preliminary study. Secondly in any class only a small number of students would be required to repeat a course and an even smaller number required to repeat multiple courses. If a formal class ranking could be used to correlate with personality measurements, then a larger number of students could be factored into these correlative studies. But a formal class ranking was not available for this preliminary study.

## Discussion

Allopathic medical schools continue to receive many more applications than class openings and therefore have an opportunity to select the “right” and “best” applicants. However the recently increasing rates of physician burnout, professional misconduct and physician suicide all raise questions as to whether we are selecting the right applicants. It is certainly possible and even plausible that non-cognitive assessments of such things as personality traits could provide potential input in the selection of candidates to decrease these negative outcomes of long term practice. Historically applicants in the US have been selected on the basis of fairly standard premedical metrics which include GPA, selected science and math GPA and MCAT scores. These metrics produce a fairly homogeneous pool of selected applicants. Yet medical school applicants are heterogeneous in terms of interests, motivations, career goals and personality traits. Personality represents a component of the human condition which has not been adequately explored in the medical school admission process nor adequately used to predict future career success or failure in medicine.

Certainly it could be argued that students who aspire to a career in family medicine to treat the underserved more likely possess different personality traits than aspiring physician-scientists who are willing to forgo the practice of the art of medicine in favor of its science. Yet probably both categories of students exhibit a similar range of traditional premed metrics like GPA and MCAT scores that serve as the gateway to their admission.

Although there have been a number of studies in the US that have examined personality traits of medical students, there have been few studies that have examined these traits as predictors of medical school performance [35–37]. And certainly there have been no studies that have examined personality factors as predictors of ultimate career success or failure. Furthermore, we are not aware of any allopathic medical school in the United States that formally uses scored personality assessments such as those of the NEO-PI-R test as a criterion in determining admission.

The US therefore lags far behind the United Kingdom and Europe in the study and use of non-cognitive tests in medical school admissions in predicting subsequent performance. It is fair to say that the US is in its infancy with regards to non-cognitive testing. The reasons for this are not entirely clear. Numerous studies in the UK, Europe and other non-US countries have investigated the role and importance of non-cognitive tests in medical school admissions and their role in predicting medical school performance [10, 41–51]. These studies used four types of non-cognitive tests including libertarian communitarian; narcissism, aloofness, confidence and empathy (NACE); self-esteem, optimism, control, self-discipline, emotional-nondefensiveness (END); and combinations thereof [10]. Performance measurements included the Educational Performance Measure (EPM) and the exit SJT. Multilevel regression analyses showed that END predicted EPM and SJT and that two facets of NACE, aloofness and empathy predicted SJT. Although these studies showed some significant correlations, they exhibited overall low effect sizes and an inconsistent picture. These personality tests consisted of a very broad range of characteristics which could be separated into so-called modifiable traits such as social and communication skills, perseverance, resilience and motivation and so-called non-modifiable traits such as neuroticism and extraversion.

These studies specifically did not use the NEO-PI-R Test which measures the so-called “Big Five”: Extraversion, Agreeableness, Conscientiousness, Neuroticism and Openness to Experience. It should again be emphasized that the NEO-PI-R Test measures non-modifiable or relatively indelible and stable aspects of personality whereas NACE is thought to measure, at least, in part modifiable traits. Measuring modifiable traits brings to any study a type of confounding which is difficult to control for. It is interesting that the one sole study conducted in Europe that did use only the “Big Five” showed that certain traits did correlate with academic performance [4].

Although the overall validity of the NEO-PI-R Test has been demonstrated in a number of studies, one can question its specific validity with respect to certain aspects.

Firstly, a rich body of research has catalogued how people’s NEO-PI scores may change over time through different ontogenetic periods of development [52]. Hence, since we are framing our argument of using these personality scores to assess psychological fit between candidates and medical school, we need to cite literature that both highlights the malleability of these traits over time but also shows consistencies in our target populations. This would enrich our contribution by making it immune to critiques that target the inherently temporal and dynamic nature of the psychometrics associated with the Big Five traits. We note that that although NEO-PI-R scores may change over time through certain ontogenetic periods of development, the majority of medical school applicants are age 22–30 and therefore would be presumed to be within closely similar ontogenetic periods.

Secondly, medical education in the US is offered to students from a wide variety of ethnic backgrounds. The extent to which the Big Five traits generalize to non-Western cultures is also therefore subject to debate and further research [53, 54]. There is literature, however, that discusses the extent to which Big Five traits can be reliably measured in non-Western participants that emphasizes that the efficacy of the tool to judge personality traits for diverse participants is adequate [14, 18, 29–31]. This is especially important in arguing that these personality assessments are not indirectly biasing admission probabilities against under-represented and marginalized communities, where the Big Five traits remain relatively under-tested. In our present study of two institutions, MUSM and CUSM, the majority of subjects were of Western heritage and hence non-Western bias would not be that confounding. Since it is anticipated that with our present policies of diversity, equity and inclusion, future classes will contain a much greater percentage of students from non-Western cultures and therefore, future studies of the NEO-PI-R will be able to directly investigate the extent to which the Big Five traits can be generalized to non-Western cultures.

Thirdly, one could question the validity of the NEO-PI-R with respect to reproducibility, subject non-compliance and cheating. There is considerable evidence of internal validity of NEO-PI-R scores with respect to test–retest and intentional test distortions although internal tests to detect cheating per se are lacking in the NEO-PI-R whereas they are preset in other personality instruments like the Minnesota Multiphasic Personality Inventory (MMPI) and the Personality Assessment Inventory (PAI) [55].

In the vast majority of the non-US studies employing the NEO-PI-R, it was made clear to the candidates that that the non-cognitive tests would not be used as a basis for admissions and so it could be argued that the candidates were also less motivated to take the test seriously. Furthermore none of these studies measured long term outcomes of medical performance.

In order to make a case that formal personality assessment has a role in the Admissions process in the United States, we first needed to show in our study that formal quantitative personality assessment correlated with medical school performance and that this correlation was observed on an unselected and therefore unbiased population. In both the MUSM and CUSM classes, this opportunity presented itself. But, it is not entirely the case that the results are on an unselected population, since presumably admission was based on multiple pieces of information. To the extent that this information, in fact, correlates with personality, we would expect indirect range restriction effects. It is therefore not the case that our sample was completely unbiased because it was biased by self-selection effects and indirect admissions decisions.

In our study we conducted a large number of blind analyses without any preconceived rationale because we did not want to bias our results. It can be argued that we approached this study largely as a fishing expedition. However this “fishing” approach was appropriate and justified given the dearth of previous studies on the utility of the NEO-PI-R in medical school admissions. Our results which not only show statistical significance but strong Pearson correlations in the setting of a relatively small sample and our demonstrations of stronger performance correlations of select NEO traits vs standard premed metrics even with the Bonferroni correction [39] also argues against a type 1 error and suggest that our preliminary studies be followed up with larger confirmatory retrospective studies and eventual validatory prospective studies.

Given that CUSM at the time of reporting this study had not even graduated a class, the true predictive value of the personality test can not yet be fully evaluated and therefore this study must be considered preliminary. In particular due to the relatively small numbers, we were only able to conduct bivariate analyses of the different personality traits and academic success. Since there are other well known predictors of academic success such as MCAT scores, that could colinearly distribute with one or more of the personality test scores, it would be important once more data is available to establish that personality scores in a multivariate model are superior or at least show that the cognitive values do not differ significantly between students with different outcomes on the personality test. Similarly although we noted that there was a difference between some of the personality values between males and females, due to the limited data which was available to us, we did not adjust for this possible confounding variable in other comparisons.

Furthermore with the growing popularity of the non-cognitive situational judgement tests such as CASPer and the SJT, it would be equally important to directly compare direct personality tests with these non-cognitive tests to determine whether personality tests have better predictive value of medical school performance. An expanded data set would allow these additional comparisons.

In any correlative or experimental study of medical education such as this one, it is important to provide the conceptual framework which serves as background. Conceptual frameworks represent ways of thinking about a problem or study [56]. Conceptual frameworks can come from theories, models or best practices but all of these can be challenged as myths, if the evidence suggests the contrary [57]. Historically it has been assumed that measurements of cognitive skills, learning, knowledge, reasoning and exam performance, largely determined by intelligence but also improved through practice, are the best predictors of not only medical school success but overall career success in medicine. However these assumptions may prove faulty as personality, a relatively indelible component of the human condition, may ultimately be more important in predicting both medical school performance as well as overall career success or failure. But the relationship of personality and intelligence is complex and there have been a number of studies examining this relationship [58–85]. Certainly intelligence influences personality although select studies have demonstrated low correlation between intelligence and the Big Five Personality Traits overall [86]. With certain personality traits, eg., Openness, intelligence certainly exerts more influence. Overall, however, intelligence influences cognitive measurements more than personality. While both are undoubtedly influenced by intelligence, intelligence certainly is not the sole determinant of either personality measurements or cognitive tests.

Furthermore it can be reasoned that if we can measure and delineate personality, we might be able to tailor individual instruction to selectively nurture individuals with certain personality traits and, in a sense, develop a form of personalized medical education. If we can achieve both, then without question, personality assessment should be used as a gateway, at least in part, to medical school admission.

## Conclusions

Our retrospective exploratory analyses of the data at MUSM and CUSM argue for the importance of measuring personality domains and facets provided by the NEO-PI-R to provide prognostic information on academic performance.

We are not yet advocating either replacing traditional premed cognitive measurements with personality measurements nor using personality measurements to supplement medical school admission assessments. We just do not know yet. That is why we did the present study and why we need future expanded studies. Studies that evaluate patient empathy or ability to relate to patients while also useful short term do not address the long term issues in the practice of medicine: physician burnout, misconduct, suicide, overall career success, career longevity and career satisfaction. Since this was a preliminary study, we had to start somewhere and we started with what performance measures were available.

Obviously, these initial and preliminary findings must be evaluated both in subsequent classes and in the present classes when more performance data, e.g. United States Medical Licensing Examination (USMLE) scores and clinical performance become available. Our retrospective analyses should be subsequently examined with both confirmatory prospective studies and future long term validation studies that examine not only medical school performance but overall career performance. These studies would fulfill the often neglected LCME mandate that medical schools in the US and Canada select applicants who possess the intelligence, integrity *and personal and emotional characteristics* necessary to become competent physicians in the practice of medicine.

## Supplementary Information

**Additional file 1.**

**Additional file 2.**

**Additional file 3.**

**Additional file 4.**

**Additional file 5.**

## Data Availability

All raw data, in de-identified format, analyzed in this study are included in this published article as its Supplementary Information (Additional files 1, 2, 3, 4 and 5).

## Abbreviations

US: United States
UK: United Kingdom
CASPer: Computer-Based Assessment for Sampling Personal Characteristics
SJT: Situational judgement test
AAMC: Association of American Medical Colleges
NEO-PI-R: Neo Personality Inventory – Revised Test
MCAT: Medical college admission test
GPA: Grade point average
FERPA: Family Educational Rights and Privacy Act
CUSM: California University of Science and Medicine
MUSM: Mercer University School of Medicine
IRB: Institutional review board
ANOVA: Analysis of variance
VR: Verbal reasoning
PS: Physical sciences
BS: Biological sciences
LCME: Liaison Committee on Medical Education
MCQs: Multiple Choice Questions
NBME: National Board of Medical Examiners
iRAT: Individual Reading Assurance Test
OSCE: Objective Structured Clinical Examination
BCPM: Biology, Chemistry, Physics, Mathematics
OLS: Ordinary Least Squares
SPSS: Statistical Product and Service Solution
NACE: Narcissism, aloofness, confidence and empathy
END: Emotional-nondefensiveness
EPM: Educational Performance Measure
MMPI: Minnesota Multiphasic Personality Inventory
PAI: Personality Assessment Inventory
USMLE: United States Medical Licensing Examination
