# Supplementary material for: A preliminary study of the probitive value of personality assessment in medical school admissions within the United States
Source: BMC Med Educ. 2022 Dec 23;22:890. doi: 10.1186/s12909-022-03901-x (PMC9783971; doi:10.1186/s12909-022-03901-x)
Supplement: Supplementary file 5 — Additional file 5. [file 12909_2022_3901_MOESM5_ESM.pdf]

```
CORRELATIONS
  /VARIABLES=neurotic extraver openness agreeabl conscien anxiety angryhos depressi selfc
ons
    impulsiv vulnerab warmth gregario assertiv activity excitsee posiemot fantasy aesthet
i feelings
    actions ideas values trust straitfo altruism complian modesty tendmind competen order
 dutifuln
    achiestr selfdisc delibera mcatver mcatbio mcatphys y1phamde y1phbmde y1hdmde y2gimde
 y2renmde
    y2idmde y1hemmde y1neumde y1bbmde y1msmde y1epieva y2carmde y2biosta y2pulmde y2endmd
e y2resdes
    ramde rbmde rasap rhdmde rhemmde rneumde rbbmde rmsmde
  /PRINT=TWOTAIL NOSIG
  /MISSING=PAIRWISE.
```

Correlations

Correlations

		(N) Neuroticism	(E) Extraversion	(O) Openness
(N) Neuroticism	Pearson Correlation	1	-.122	-.073
	Sig. (2-tailed)		.193	.435
	N	116	116	116
(E) Extraversion	Pearson Correlation	-.122	1	-.048
	Sig. (2-tailed)	.193		.607
	N	116	116	116
(O) Openness	Pearson Correlation	-.073	-.048	1
	Sig. (2-tailed)	.435	.607	
	N	116	116	116
(A) Agreeableness	Pearson Correlation	-.191*	.312**	.010
	Sig. (2-tailed)	.040	.001	.914
	N	116	116	116
(C) Conscientiousness	Pearson Correlation	-.012	.032	-.118
	Sig. (2-tailed)	.896	.729	.205
	N	116	116	116
(N1) Anxiety	Pearson Correlation	.818**	-.026	-.093
	Sig. (2-tailed)	.000	.778	.320
	N	116	116	116

Correlations

		(A) Agreeableness	(C) Conscientiousness	(N1) Anxiety
(N) Neuroticism	Pearson Correlation	-.191*	-.012	.818**
	Sig. (2-tailed)	.040	.896	.000
	N	116	116	116
(E) Extraversion	Pearson Correlation	.312**	.032	-.026
	Sig. (2-tailed)	.001	.729	.778
	N	116	116	116
(O) Openness	Pearson Correlation	.010	-.118	-.093
	Sig. (2-tailed)	.914	.205	.320
	N	116	116	116
(A) Agreeableness	Pearson Correlation	1	.241**	-.102
	Sig. (2-tailed)		.009	.277
	N	116	116	116
(C) Conscientiousness	Pearson Correlation	.241**	1	.082
	Sig. (2-tailed)	.009		.380
	N	116	116	116
(N1) Anxiety	Pearson Correlation	-.102	.082	1
	Sig. (2-tailed)	.277	.380	
	N	116	116	116

Correlations

		(N2) Angry Hostility	(N3) Depression	(N4) Self-Consciousness
(N) Neuroticism	Pearson Correlation	.635**	.799**	.730**
	Sig. (2-tailed)	.000	.000	.000
	N	116	116	116
(E) Extraversion	Pearson Correlation	-.289**	-.299**	-.316**
	Sig. (2-tailed)	.002	.001	.001
	N	116	116	116
(O) Openness	Pearson Correlation	-.015	.001	-.114
	Sig. (2-tailed)	.875	.987	.225
	N	116	116	116
(A) Agreeableness	Pearson Correlation	-.668**	-.363**	-.161
	Sig. (2-tailed)	.000	.000	.084
	N	116	116	116
(C) Conscientiousness	Pearson Correlation	-.208*	-.333**	-.297**
	Sig. (2-tailed)	.025	.000	.001
	N	116	116	116
(N1) Anxiety	Pearson Correlation	.406**	.577**	.469**
	Sig. (2-tailed)	.000	.000	.000
	N	116	116	116

Correlations

		(N5) Impulsiveness	(N6) Vulnerability	(E1) Warmth
(N) Neuroticism	Pearson Correlation	.506**	.722**	-.184*
	Sig. (2-tailed)	.000	.000	.048
	N	116	116	116
(E) Extraversion	Pearson Correlation	.124	-.200*	.721**
	Sig. (2-tailed)	.183	.032	.000
	N	116	116	116
(O) Openness	Pearson Correlation	.043	-.179	-.048
	Sig. (2-tailed)	.649	.055	.609
	N	116	116	116
(A) Agreeableness	Pearson Correlation	-.307**	-.228*	.580**
	Sig. (2-tailed)	.001	.014	.000
	N	116	116	116
(C) Conscientiousness	Pearson Correlation	-.464**	-.384**	.280**
	Sig. (2-tailed)	.000	.000	.002
	N	116	116	116
(N1) Anxiety	Pearson Correlation	.329**	.558**	-.067
	Sig. (2-tailed)	.000	.000	.474
	N	116	116	116

Correlations

		(E2) Gregariousness	(E3) Assertiveness	(E4) Activity
(N) Neuroticism	Pearson Correlation	-.202*	-.256**	-.137
	Sig. (2-tailed)	.030	.006	.143
	N	116	116	116
(E) Extraversion	Pearson Correlation	.762**	.438**	.543**
	Sig. (2-tailed)	.000	.000	.000
	N	116	116	116
(O) Openness	Pearson Correlation	-.015	.050	.051
	Sig. (2-tailed)	.873	.596	.587
	N	116	116	116
(A) Agreeableness	Pearson Correlation	.177	-.076	.116
	Sig. (2-tailed)	.057	.416	.215
	N	116	116	116
(C) Conscientiousness	Pearson Correlation	.067	.326**	.426**
	Sig. (2-tailed)	.473	.000	.000
	N	116	116	116
(N1) Anxiety	Pearson Correlation	-.045	-.141	-.014
	Sig. (2-tailed)	.634	.132	.879
	N	116	116	116

Correlations

		(E5) Excitement-Seeking	(E6) Positive Emotions	(O1) Fantasy
(N) Neuroticism	Pearson Correlation	-.064	-.140	.141
	Sig. (2-tailed)	.495	.134	.132
	N	116	116	116
(E) Extraversion	Pearson Correlation	.453**	.736**	.109
	Sig. (2-tailed)	.000	.000	.245
	N	116	116	116
(O) Openness	Pearson Correlation	.119	.254**	.583**
	Sig. (2-tailed)	.204	.006	.000
	N	116	116	116
(A) Agreeableness	Pearson Correlation	-.205*	.415**	-.257**
	Sig. (2-tailed)	.027	.000	.005
	N	116	116	116
(C) Conscientiousness	Pearson Correlation	-.134	.067	-.357**
	Sig. (2-tailed)	.153	.472	.000
	N	116	116	116
(N1) Anxiety	Pearson Correlation	-.111	-.090	.075
	Sig. (2-tailed)	.237	.339	.426
	N	116	116	116

Correlations

		(O2) Aesthetics	(O3) Feelings	(O4) Actions
(N) Neuroticism	Pearson Correlation	.038	.403**	-.382**
	Sig. (2-tailed)	.687	.000	.000
	N	116	116	116
(E) Extraversion	Pearson Correlation	.093	.375**	.138
	Sig. (2-tailed)	.323	.000	.141
	N	116	116	116
(O) Openness	Pearson Correlation	.762**	.429**	.524**
	Sig. (2-tailed)	.000	.000	.000
	N	116	116	116
(A) Agreeableness	Pearson Correlation	.112	.063	.081
	Sig. (2-tailed)	.231	.499	.385
	N	116	116	116
(C) Conscientiousness	Pearson Correlation	.019	-.016	-.183*
	Sig. (2-tailed)	.836	.867	.049
	N	116	116	116
(N1) Anxiety	Pearson Correlation	-.023	.290**	-.340**
	Sig. (2-tailed)	.809	.002	.000
	N	116	116	116

Correlations

		(O5) Ideas	(O6) Values	(A1) Trust	(A2) Straightforwardness
(N) Neuroticism	Pearson Correlation	-.119	-.144	-.416**	-.163
	Sig. (2-tailed)	.204	.124	.000	.080
	N	116	116	116	116
(E) Extraversion	Pearson Correlation	.008	-.075	.349**	.169
	Sig. (2-tailed)	.935	.424	.000	.070
	N	116	116	116	116
(O) Openness	Pearson Correlation	.787**	.502**	.135	-.176
	Sig. (2-tailed)	.000	.000	.149	.058
	N	116	116	116	116
(A) Agreeableness	Pearson Correlation	.092	.051	.684**	.789**
	Sig. (2-tailed)	.327	.586	.000	.000
	N	116	116	116	116
(C) Conscientiousness	Pearson Correlation	.166	-.164	.143	.438**
	Sig. (2-tailed)	.075	.079	.126	.000
	N	116	116	116	116
(N1) Anxiety	Pearson Correlation	-.085	-.053	-.244**	-.071
	Sig. (2-tailed)	.367	.574	.008	.447
	N	116	116	116	116

Correlations

		(A3) Altruism	(A4) Compliance	(A5) Modesty
(N) Neuroticism	Pearson Correlation	-.283**	-.247**	.131
	Sig. (2-tailed)	.002	.007	.160
	N	116	116	116
(E) Extraversion	Pearson Correlation	.587**	.134	.014
	Sig. (2-tailed)	.000	.151	.881
	N	116	116	116
(O) Openness	Pearson Correlation	.032	.007	-.118
	Sig. (2-tailed)	.732	.944	.208
	N	116	116	116
(A) Agreeableness	Pearson Correlation	.751**	.806**	.481**
	Sig. (2-tailed)	.000	.000	.000
	N	116	116	116
(C) Conscientiousness	Pearson Correlation	.367**	.233*	-.022
	Sig. (2-tailed)	.000	.012	.813
	N	116	116	116
(N1) Anxiety	Pearson Correlation	-.158	-.126	.005
	Sig. (2-tailed)	.090	.177	.960
	N	116	116	116

Correlations

		(A6) Tender-Mindedness	(C1) Competence	(C2) Order
(N) Neuroticism	Pearson Correlation	-.123	-.409**	.124
	Sig. (2-tailed)	.188	.000	.186
	N	116	116	116
(E) Extraversion	Pearson Correlation	.507**	.248**	.086
	Sig. (2-tailed)	.000	.007	.360
	N	116	116	116
(O) Openness	Pearson Correlation	.132	.033	-.240**
	Sig. (2-tailed)	.159	.722	.009
	N	116	116	116
(A) Agreeableness	Pearson Correlation	.670**	.287**	.181
	Sig. (2-tailed)	.000	.002	.051
	N	116	116	116
(C) Conscientiousness	Pearson Correlation	.222*	.705**	.737**
	Sig. (2-tailed)	.017	.000	.000
	N	116	116	116
(N1) Anxiety	Pearson Correlation	-.080	-.286**	.213*
	Sig. (2-tailed)	.392	.002	.022
	N	116	116	116

Correlations

		(C3) Dutifulness	(C4) Achievement Striving	(C5) Self-Discipline
(N) Neuroticism	Pearson Correlation	-.388**	.013	-.436**
	Sig. (2-tailed)	.000	.887	.000
	N	116	116	116
(E) Extraversion	Pearson Correlation	.109	.128	.163
	Sig. (2-tailed)	.246	.172	.080
	N	116	116	116
(O) Openness	Pearson Correlation	-.093	.024	.002
	Sig. (2-tailed)	.318	.794	.981
	N	116	116	116
(A) Agreeableness	Pearson Correlation	.454**	.110	.308**
	Sig. (2-tailed)	.000	.240	.001
	N	116	116	116
(C) Conscientiousness	Pearson Correlation	.748**	.807**	.769**
	Sig. (2-tailed)	.000	.000	.000
	N	116	116	116
(N1) Anxiety	Pearson Correlation	-.264**	.086	-.255**
	Sig. (2-tailed)	.004	.357	.006
	N	116	116	116

Correlations

		(C6) Deliberation	MCAT Verbal	MCAT Biological Sciences
(N) Neuroticism	Pearson Correlation	-.138	.013	-.008
	Sig. (2-tailed)	.140	.887	.929
	N	116	116	116
(E) Extraversion	Pearson Correlation	-.150	-.223*	-.236*
	Sig. (2-tailed)	.109	.016	.011
	N	116	116	116
(O) Openness	Pearson Correlation	-.149	.160	.235*
	Sig. (2-tailed)	.110	.086	.011
	N	116	116	116
(A) Agreeableness	Pearson Correlation	.292**	-.143	.024
	Sig. (2-tailed)	.001	.127	.796
	N	116	116	116
(C) Conscientiousness	Pearson Correlation	.652**	-.229*	-.058
	Sig. (2-tailed)	.000	.014	.533
	N	116	116	116
(N1) Anxiety	Pearson Correlation	-.079	-.064	-.084
	Sig. (2-tailed)	.400	.496	.368
	N	116	116	116

Correlations

		MCAT Physical Sciences	Yr 1 Phase A MDE	Yr 1 Phase B MDE
(N) Neuroticism	Pearson Correlation	.005	-.077	-.144
	Sig. (2-tailed)	.958	.411	.124
	N	116	115	115
(E) Extraversion	Pearson Correlation	-.069	-.193*	-.154
	Sig. (2-tailed)	.464	.038	.100
	N	116	115	115
(O) Openness	Pearson Correlation	.182	-.038	-.054
	Sig. (2-tailed)	.051	.685	.570
	N	116	115	115
(A) Agreeableness	Pearson Correlation	-.037	.026	.042
	Sig. (2-tailed)	.695	.784	.655
	N	116	115	115
(C) Conscientiousness	Pearson Correlation	-.207*	-.019	.030
	Sig. (2-tailed)	.026	.837	.747
	N	116	115	115
(N1) Anxiety	Pearson Correlation	-.053	-.088	-.143
	Sig. (2-tailed)	.575	.349	.128
	N	116	115	115

Correlations

		Yr 1 Host Defense MDE	Yr 2 GI MDE	Yr 2 Renal MDE
(N) Neuroticism	Pearson Correlation	-.006	.005	-.077
	Sig. (2-tailed)	.947	.973	.615
	N	113	45	45
(E) Extraversion	Pearson Correlation	-.047	.189	.097
	Sig. (2-tailed)	.620	.214	.526
	N	113	45	45
(O) Openness	Pearson Correlation	-.125	-.029	-.152
	Sig. (2-tailed)	.186	.852	.319
	N	113	45	45
(A) Agreeableness	Pearson Correlation	.095	.206	.049
	Sig. (2-tailed)	.317	.175	.747
	N	113	45	45
(C) Conscientiousness	Pearson Correlation	.153	.347*	.143
	Sig. (2-tailed)	.106	.020	.348
	N	113	45	45
(N1) Anxiety	Pearson Correlation	.000	-.033	-.096
	Sig. (2-tailed)	1.000	.829	.531
	N	113	45	45

Correlations

		Yr 2 Infectious Diseases MDE	Yr 1 Hematology MDE	Yr 1 Neurology MDE
(N) Neuroticism	Pearson Correlation	.013	-.001	-.165
	Sig. (2-tailed)	.935	.991	.279
	N	45	109	45
(E) Extraversion	Pearson Correlation	.057	-.007	.077
	Sig. (2-tailed)	.711	.944	.616
	N	45	109	45
(O) Openness	Pearson Correlation	-.246	-.029	-.122
	Sig. (2-tailed)	.103	.767	.423
	N	45	109	45
(A) Agreeableness	Pearson Correlation	.090	.031	.091
	Sig. (2-tailed)	.555	.753	.551
	N	45	109	45
(C) Conscientiousness	Pearson Correlation	.313*	.169	.382**
	Sig. (2-tailed)	.036	.079	.010
	N	45	109	45
(N1) Anxiety	Pearson Correlation	-.007	-.012	-.213
	Sig. (2-tailed)	.964	.904	.159
	N	45	109	45

Correlations

		Yr 1 Brain & Behavior MDE	Yr 1 Musculoskeletal MDE	Yr 1 Community Epidimiology Study Grade
(N) Neuroticism	Pearson Correlation	-.008	-.157	-.050
	Sig. (2-tailed)	.944	.301	.597
	N	77	45	115
(E) Extraversion	Pearson Correlation	-.085	.077	-.194*
	Sig. (2-tailed)	.462	.617	.037
	N	77	45	115
(O) Openness	Pearson Correlation	-.024	-.034	-.012
	Sig. (2-tailed)	.839	.823	.895
	N	77	45	115
(A) Agreeableness	Pearson Correlation	.050	.155	-.147
	Sig. (2-tailed)	.665	.310	.116
	N	77	45	115
(C) Conscientiousness	Pearson Correlation	.154	.243	-.069
	Sig. (2-tailed)	.182	.108	.463
	N	77	45	115
(N1) Anxiety	Pearson Correlation	-.088	-.202	-.049
	Sig. (2-tailed)	.447	.183	.606
	N	77	45	115

Correlations

		Yr 2 Cardiology MDE	Yr 2 Biostatistics	Yr 2 Pulmonology MDE
(N) Neuroticism	Pearson Correlation	-.232	.058	-.190
	Sig. (2-tailed)	.125	.706	.211
	N	45	45	45
(E) Extraversion	Pearson Correlation	.023	-.014	.012
	Sig. (2-tailed)	.878	.929	.938
	N	45	45	45
(O) Openness	Pearson Correlation	-.080	.128	.079
	Sig. (2-tailed)	.599	.403	.604
	N	45	45	45
(A) Agreeableness	Pearson Correlation	.080	-.031	.081
	Sig. (2-tailed)	.601	.840	.596
	N	45	45	45
(C) Conscientiousness	Pearson Correlation	.282	.032	.158
	Sig. (2-tailed)	.060	.836	.299
	N	45	45	45
(N1) Anxiety	Pearson Correlation	-.234	.110	-.222
	Sig. (2-tailed)	.122	.473	.143
	N	45	45	45

Correlations

		Yr 2 Endocrinology MDE	Yr 2 Research Design	Repeat Phase A MDE
(N) Neuroticism	Pearson Correlation	-.023	.062	.107
	Sig. (2-tailed)	.880	.684	.784
	N	46	45	9
(E) Extraversion	Pearson Correlation	.041	.065	.187
	Sig. (2-tailed)	.788	.670	.630
	N	46	45	9
(O) Openness	Pearson Correlation	-.178	.054	-.572
	Sig. (2-tailed)	.236	.724	.108
	N	46	45	9
(A) Agreeableness	Pearson Correlation	.047	-.031	-.063
	Sig. (2-tailed)	.757	.841	.872
	N	46	45	9
(C) Conscientiousness	Pearson Correlation	.112	.063	-.313
	Sig. (2-tailed)	.460	.679	.412
	N	46	45	9
(N1) Anxiety	Pearson Correlation	-.087	.023	.359
	Sig. (2-tailed)	.567	.880	.342
	N	46	45	9

Correlations

		Repeat Phase B MDE	Repeat Phase A SAP	Repeat Host Defense MDE
(N) Neuroticism	Pearson Correlation	-.457	-.281	-.115
	Sig. (2-tailed)	.217	.463	.768
	N	9	9	9
(E) Extraversion	Pearson Correlation	.541	.247	.216
	Sig. (2-tailed)	.133	.522	.577
	N	9	9	9
(O) Openness	Pearson Correlation	.047	-.046	-.798**
	Sig. (2-tailed)	.904	.907	.010
	N	9	9	9
(A) Agreeableness	Pearson Correlation	-.193	-.096	.018
	Sig. (2-tailed)	.620	.807	.962
	N	9	9	9
(C) Conscientiousness	Pearson Correlation	-.054	.311	-.127
	Sig. (2-tailed)	.891	.415	.745
	N	9	9	9
(N1) Anxiety	Pearson Correlation	-.381	-.217	.276
	Sig. (2-tailed)	.311	.575	.473
	N	9	9	9

Correlations

		Repeat Hematology MDE	Repeat Neurology MDE	Repeat Brain & Behavior MDE
(N) Neuroticism	Pearson Correlation	.123	.047	-.456
	Sig. (2-tailed)	.753	.904	.217
	N	9	9	9
(E) Extraversion	Pearson Correlation	.177	-.058	.282
	Sig. (2-tailed)	.648	.881	.462
	N	9	9	9
(O) Openness	Pearson Correlation	.543	.717*	-.001
	Sig. (2-tailed)	.131	.030	.997
	N	9	9	9
(A) Agreeableness	Pearson Correlation	.213	-.205	.462
	Sig. (2-tailed)	.583	.597	.211
	N	9	9	9
(C) Conscientiousness	Pearson Correlation	-.437	.030	.698*
	Sig. (2-tailed)	.240	.939	.037
	N	9	9	9
(N1) Anxiety	Pearson Correlation	.029	-.365	-.294
	Sig. (2-tailed)	.940	.334	.442
	N	9	9	9

Correlations

		Repeat Musculoskeletal MDE
(N) Neuroticism	Pearson Correlation	-.381
	Sig. (2-tailed)	.311
	N	9
(E) Extraversion	Pearson Correlation	.341
	Sig. (2-tailed)	.369
	N	9
(O) Openness	Pearson Correlation	-.506
	Sig. (2-tailed)	.165
	N	9
(A) Agreeableness	Pearson Correlation	.622
	Sig. (2-tailed)	.074
	N	9
(C) Conscientiousness	Pearson Correlation	.234
	Sig. (2-tailed)	.544
	N	9
(N1) Anxiety	Pearson Correlation	-.232
	Sig. (2-tailed)	.547
	N	9

Correlations

		(N) Neuroticism	(E) Extraversion	(O) Openness
(N2) Angry Hostility	Pearson Correlation	.635**	-.289**	-.015
	Sig. (2-tailed)	.000	.002	.875
	N	116	116	116
(N3) Depression	Pearson Correlation	.799**	-.299**	.001
	Sig. (2-tailed)	.000	.001	.987
	N	116	116	116
(N4) Self-Consciousness	Pearson Correlation	.730**	-.316**	-.114
	Sig. (2-tailed)	.000	.001	.225
	N	116	116	116
(N5) Impulsiveness	Pearson Correlation	.506**	.124	.043
	Sig. (2-tailed)	.000	.183	.649
	N	116	116	116
(N6) Vulnerability	Pearson Correlation	.722**	-.200*	-.179
	Sig. (2-tailed)	.000	.032	.055
	N	116	116	116
(E1) Warmth	Pearson Correlation	-.184*	.721**	-.048
	Sig. (2-tailed)	.048	.000	.609
	N	116	116	116
(E2) Gregariousness	Pearson Correlation	-.202*	.762**	-.015
	Sig. (2-tailed)	.030	.000	.873
	N	116	116	116
(E3) Assertiveness	Pearson Correlation	-.256**	.438**	.050
	Sig. (2-tailed)	.006	.000	.596
	N	116	116	116
(E4) Activity	Pearson Correlation	-.137	.543**	.051
	Sig. (2-tailed)	.143	.000	.587
	N	116	116	116
(E5) Excitement-Seeking	Pearson Correlation	-.064	.453**	.119
	Sig. (2-tailed)	.495	.000	.204
	N	116	116	116

Correlations

		(A) Agreeableness	(C) Conscientiousness	(N1) Anxiety
(N2) Angry Hostility	Pearson Correlation	-.668**	-.208*	.406**
	Sig. (2-tailed)	.000	.025	.000
	N	116	116	116
(N3) Depression	Pearson Correlation	-.363**	-.333**	.577**
	Sig. (2-tailed)	.000	.000	.000
	N	116	116	116
(N4) Self-Consciousness	Pearson Correlation	-.161	-.297**	.469**
	Sig. (2-tailed)	.084	.001	.000
	N	116	116	116
(N5) Impulsiveness	Pearson Correlation	-.307**	-.464**	.329**
	Sig. (2-tailed)	.001	.000	.000
	N	116	116	116
(N6) Vulnerability	Pearson Correlation	-.228*	-.384**	.558**
	Sig. (2-tailed)	.014	.000	.000
	N	116	116	116
(E1) Warmth	Pearson Correlation	.580**	.280**	-.067
	Sig. (2-tailed)	.000	.002	.474
	N	116	116	116
(E2) Gregariousness	Pearson Correlation	.177	.067	-.045
	Sig. (2-tailed)	.057	.473	.634
	N	116	116	116
(E3) Assertiveness	Pearson Correlation	-.076	.326**	-.141
	Sig. (2-tailed)	.416	.000	.132
	N	116	116	116
(E4) Activity	Pearson Correlation	.116	.426**	-.014
	Sig. (2-tailed)	.215	.000	.879
	N	116	116	116
(E5) Excitement-Seeking	Pearson Correlation	-.205*	-.134	-.111
	Sig. (2-tailed)	.027	.153	.237
	N	116	116	116

Correlations

		(N2) Angry Hostility	(N3) Depression	(N4) Self-Consciousness
(N2) Angry Hostility	Pearson Correlation	1	.618**	.478**
	Sig. (2-tailed)		.000	.000
	N	116	116	116
(N3) Depression	Pearson Correlation	.618**	1	.704**
	Sig. (2-tailed)	.000		.000
	N	116	116	116
(N4) Self-Consciousness	Pearson Correlation	.478**	.704**	1
	Sig. (2-tailed)	.000	.000	
	N	116	116	116
(N5) Impulsiveness	Pearson Correlation	.402**	.467**	.499**
	Sig. (2-tailed)	.000	.000	.000
	N	116	116	116
(N6) Vulnerability	Pearson Correlation	.488**	.706**	.577**
	Sig. (2-tailed)	.000	.000	.000
	N	116	116	116
(E1) Warmth	Pearson Correlation	-.388**	-.390**	-.340**
	Sig. (2-tailed)	.000	.000	.000
	N	116	116	116
(E2) Gregariousness	Pearson Correlation	-.281**	-.323**	-.310**
	Sig. (2-tailed)	.002	.000	.001
	N	116	116	116
(E3) Assertiveness	Pearson Correlation	-.108	-.273**	-.437**
	Sig. (2-tailed)	.249	.003	.000
	N	116	116	116
(E4) Activity	Pearson Correlation	-.139	-.342**	-.362**
	Sig. (2-tailed)	.137	.000	.000
	N	116	116	116
(E5) Excitement-Seeking	Pearson Correlation	-.023	-.003	-.103
	Sig. (2-tailed)	.803	.974	.272
	N	116	116	116

Correlations

		(N5) Impulsiveness	(N6) Vulnerability	(E1) Warmth
(N2) Angry Hostility	Pearson Correlation	.402**	.488**	-.388**
	Sig. (2-tailed)	.000	.000	.000
	N	116	116	116
(N3) Depression	Pearson Correlation	.467**	.706**	-.390**
	Sig. (2-tailed)	.000	.000	.000
	N	116	116	116
(N4) Self-Consciousness	Pearson Correlation	.499**	.577**	-.340**
	Sig. (2-tailed)	.000	.000	.000
	N	116	116	116
(N5) Impulsiveness	Pearson Correlation	1	.443**	-.201*
	Sig. (2-tailed)		.000	.031
	N	116	116	116
(N6) Vulnerability	Pearson Correlation	.443**	1	-.290**
	Sig. (2-tailed)	.000		.002
	N	116	116	116
(E1) Warmth	Pearson Correlation	-.201*	-.290**	1
	Sig. (2-tailed)	.031	.002	
	N	116	116	116
(E2) Gregariousness	Pearson Correlation	-.038	-.190*	.516**
	Sig. (2-tailed)	.682	.041	.000
	N	116	116	116
(E3) Assertiveness	Pearson Correlation	-.127	-.340**	.356**
	Sig. (2-tailed)	.176	.000	.000
	N	116	116	116
(E4) Activity	Pearson Correlation	-.210*	-.410**	.480**
	Sig. (2-tailed)	.024	.000	.000
	N	116	116	116
(E5) Excitement-Seeking	Pearson Correlation	.173	-.012	.052
	Sig. (2-tailed)	.064	.900	.582
	N	116	116	116

Correlations

		(E2) Gregariousness	(E3) Assertiveness	(E4) Activity
(N2) Angry Hostility	Pearson Correlation	-.281**	-.108	-.139
	Sig. (2-tailed)	.002	.249	.137
	N	116	116	116
(N3) Depression	Pearson Correlation	-.323**	-.273**	-.342**
	Sig. (2-tailed)	.000	.003	.000
	N	116	116	116
(N4) Self-Consciousness	Pearson Correlation	-.310**	-.437**	-.362**
	Sig. (2-tailed)	.001	.000	.000
	N	116	116	116
(N5) Impulsiveness	Pearson Correlation	-.038	-.127	-.210*
	Sig. (2-tailed)	.682	.176	.024
	N	116	116	116
(N6) Vulnerability	Pearson Correlation	-.190*	-.340**	-.410**
	Sig. (2-tailed)	.041	.000	.000
	N	116	116	116
(E1) Warmth	Pearson Correlation	.516**	.356**	.480**
	Sig. (2-tailed)	.000	.000	.000
	N	116	116	116
(E2) Gregariousness	Pearson Correlation	1	.348**	.421**
	Sig. (2-tailed)		.000	.000
	N	116	116	116
(E3) Assertiveness	Pearson Correlation	.348**	1	.457**
	Sig. (2-tailed)	.000		.000
	N	116	116	116
(E4) Activity	Pearson Correlation	.421**	.457**	1
	Sig. (2-tailed)	.000	.000	
	N	116	116	116
(E5) Excitement-Seeking	Pearson Correlation	.495**	.188*	.137
	Sig. (2-tailed)	.000	.043	.141
	N	116	116	116

Correlations

		(E5) Excitement-Seeking	(E6) Positive Emotions	(O1) Fantasy
(N2) Angry Hostility	Pearson Correlation	-.023	-.325**	.233*
	Sig. (2-tailed)	.803	.000	.012
	N	116	116	116
(N3) Depression	Pearson Correlation	-.003	-.290**	.237*
	Sig. (2-tailed)	.974	.002	.011
	N	116	116	116
(N4) Self-Consciousness	Pearson Correlation	-.103	-.262**	.042
	Sig. (2-tailed)	.272	.004	.652
	N	116	116	116
(N5) Impulsiveness	Pearson Correlation	.173	-.027	.313**
	Sig. (2-tailed)	.064	.775	.001
	N	116	116	116
(N6) Vulnerability	Pearson Correlation	-.012	-.306**	.116
	Sig. (2-tailed)	.900	.001	.215
	N	116	116	116
(E1) Warmth	Pearson Correlation	.052	.555**	-.055
	Sig. (2-tailed)	.582	.000	.559
	N	116	116	116
(E2) Gregariousness	Pearson Correlation	.495**	.425**	.047
	Sig. (2-tailed)	.000	.000	.619
	N	116	116	116
(E3) Assertiveness	Pearson Correlation	.188*	.232*	.022
	Sig. (2-tailed)	.043	.012	.812
	N	116	116	116
(E4) Activity	Pearson Correlation	.137	.355**	-.061
	Sig. (2-tailed)	.141	.000	.515
	N	116	116	116
(E5) Excitement-Seeking	Pearson Correlation	1	.192*	.214*
	Sig. (2-tailed)		.039	.021
	N	116	116	116

Correlations

		(O2) Aesthetics	(O3) Feelings	(O4) Actions
(N2) Angry Hostility	Pearson Correlation	-.096	.116	-.187*
	Sig. (2-tailed)	.305	.215	.044
	N	116	116	116
(N3) Depression	Pearson Correlation	-.019	.218*	-.241**
	Sig. (2-tailed)	.836	.019	.009
	N	116	116	116
(N4) Self-Consciousness	Pearson Correlation	-.037	.049	-.267**
	Sig. (2-tailed)	.695	.599	.004
	N	116	116	116
(N5) Impulsiveness	Pearson Correlation	.002	.235*	-.108
	Sig. (2-tailed)	.984	.011	.247
	N	116	116	116
(N6) Vulnerability	Pearson Correlation	-.097	.169	-.259**
	Sig. (2-tailed)	.300	.069	.005
	N	116	116	116
(E1) Warmth	Pearson Correlation	.088	.163	.048
	Sig. (2-tailed)	.348	.080	.611
	N	116	116	116
(E2) Gregariousness	Pearson Correlation	.168	.236*	.186*
	Sig. (2-tailed)	.072	.011	.046
	N	116	116	116
(E3) Assertiveness	Pearson Correlation	.046	.061	.058
	Sig. (2-tailed)	.625	.514	.534
	N	116	116	116
(E4) Activity	Pearson Correlation	.126	.157	.153
	Sig. (2-tailed)	.178	.093	.102
	N	116	116	116
(E5) Excitement-Seeking	Pearson Correlation	.168	.202*	.224*
	Sig. (2-tailed)	.071	.029	.016
	N	116	116	116

Correlations

		(O5) Ideas	(O6) Values	(A1) Trust	(A2) Straightforwardness
(N2) Angry Hostility	Pearson Correlation	-.082	-.078	-.594**	-.519**
	Sig. (2-tailed)	.380	.404	.000	.000
	N	116	116	116	116
(N3) Depression	Pearson Correlation	-.140	-.071	-.510**	-.401**
	Sig. (2-tailed)	.133	.447	.000	.000
	N	116	116	116	116
(N4) Self-Consciousness	Pearson Correlation	-.207*	-.066	-.337**	-.241**
	Sig. (2-tailed)	.026	.483	.000	.009
	N	116	116	116	116
(N5) Impulsiveness	Pearson Correlation	-.011	.028	-.244**	-.383**
	Sig. (2-tailed)	.907	.766	.008	.000
	N	116	116	116	116
(N6) Vulnerability	Pearson Correlation	-.343**	-.099	-.327**	-.223*
	Sig. (2-tailed)	.000	.292	.000	.016
	N	116	116	116	116
(E1) Warmth	Pearson Correlation	.085	-.080	.460**	.403**
	Sig. (2-tailed)	.366	.395	.000	.000
	N	116	116	116	116
(E2) Gregariousness	Pearson Correlation	.031	-.028	.217*	.136
	Sig. (2-tailed)	.744	.764	.019	.145
	N	116	116	116	116
(E3) Assertiveness	Pearson Correlation	.154	-.005	.062	-.034
	Sig. (2-tailed)	.099	.960	.506	.720
	N	116	116	116	116
(E4) Activity	Pearson Correlation	.140	-.058	.258**	.125
	Sig. (2-tailed)	.133	.537	.005	.183
	N	116	116	116	116
(E5) Excitement-Seeking	Pearson Correlation	.177	-.049	-.113	-.199*
	Sig. (2-tailed)	.057	.599	.227	.033
	N	116	116	116	116

Correlations

		(A3) Altruism	(A4) Compliance	(A5) Modesty
(N2) Angry Hostility	Pearson Correlation	-.559**	-.612**	-.176
	Sig. (2-tailed)	.000	.000	.059
	N	116	116	116
(N3) Depression	Pearson Correlation	-.516**	-.302**	.076
	Sig. (2-tailed)	.000	.001	.419
	N	116	116	116
(N4) Self-Consciousness	Pearson Correlation	-.393**	-.131	.177
	Sig. (2-tailed)	.000	.162	.057
	N	116	116	116
(N5) Impulsiveness	Pearson Correlation	-.252**	-.322**	.002
	Sig. (2-tailed)	.006	.000	.983
	N	116	116	116
(N6) Vulnerability	Pearson Correlation	-.431**	-.255**	.060
	Sig. (2-tailed)	.000	.006	.520
	N	116	116	116
(E1) Warmth	Pearson Correlation	.707**	.397**	.164
	Sig. (2-tailed)	.000	.000	.078
	N	116	116	116
(E2) Gregariousness	Pearson Correlation	.402**	.061	-.071
	Sig. (2-tailed)	.000	.515	.449
	N	116	116	116
(E3) Assertiveness	Pearson Correlation	.192*	.021	-.136
	Sig. (2-tailed)	.039	.823	.146
	N	116	116	116
(E4) Activity	Pearson Correlation	.352**	.048	-.010
	Sig. (2-tailed)	.000	.608	.918
	N	116	116	116
(E5) Excitement-Seeking	Pearson Correlation	.033	-.158	-.047
	Sig. (2-tailed)	.721	.090	.615
	N	116	116	116

Correlations

		(A6) Tender-Mindedness	(C1) Competence	(C2) Order
(N2) Angry Hostility	Pearson Correlation	-.402**	-.451**	-.124
	Sig. (2-tailed)	.000	.000	.185
	N	116	116	116
(N3) Depression	Pearson Correlation	-.281**	-.554**	-.197*
	Sig. (2-tailed)	.002	.000	.034
	N	116	116	116
(N4) Self-Consciousness	Pearson Correlation	-.251**	-.517**	-.171
	Sig. (2-tailed)	.007	.000	.066
	N	116	116	116
(N5) Impulsiveness	Pearson Correlation	-.157	-.380**	-.277**
	Sig. (2-tailed)	.092	.000	.003
	N	116	116	116
(N6) Vulnerability	Pearson Correlation	-.237*	-.640**	-.098
	Sig. (2-tailed)	.010	.000	.297
	N	116	116	116
(E1) Warmth	Pearson Correlation	.479**	.369**	.199*
	Sig. (2-tailed)	.000	.000	.032
	N	116	116	116
(E2) Gregariousness	Pearson Correlation	.291**	.229*	.086
	Sig. (2-tailed)	.002	.013	.359
	N	116	116	116
(E3) Assertiveness	Pearson Correlation	.255**	.323**	.195*
	Sig. (2-tailed)	.006	.000	.036
	N	116	116	116
(E4) Activity	Pearson Correlation	.331**	.391**	.187*
	Sig. (2-tailed)	.000	.000	.044
	N	116	116	116
(E5) Excitement-Seeking	Pearson Correlation	.006	.044	-.086
	Sig. (2-tailed)	.945	.639	.359
	N	116	116	116

Correlations

		(C3) Dutifulness	(C4) Achievement Striving	(C5) Self-Discipline
(N2) Angry Hostility	Pearson Correlation	-.503**	-.155	-.528**
	Sig. (2-tailed)	.000	.097	.000
	N	116	116	116
(N3) Depression	Pearson Correlation	-.568**	-.256**	-.581**
	Sig. (2-tailed)	.000	.006	.000
	N	116	116	116
(N4) Self-Consciousness	Pearson Correlation	-.480**	-.291**	-.536**
	Sig. (2-tailed)	.000	.002	.000
	N	116	116	116
(N5) Impulsiveness	Pearson Correlation	-.525**	-.289**	-.578**
	Sig. (2-tailed)	.000	.002	.000
	N	116	116	116
(N6) Vulnerability	Pearson Correlation	-.550**	-.286**	-.565**
	Sig. (2-tailed)	.000	.002	.000
	N	116	116	116
(E1) Warmth	Pearson Correlation	.364**	.242**	.322**
	Sig. (2-tailed)	.000	.009	.000
	N	116	116	116
(E2) Gregariousness	Pearson Correlation	.130	.132	.223*
	Sig. (2-tailed)	.165	.158	.016
	N	116	116	116
(E3) Assertiveness	Pearson Correlation	.277**	.365**	.366**
	Sig. (2-tailed)	.003	.000	.000
	N	116	116	116
(E4) Activity	Pearson Correlation	.287**	.460**	.400**
	Sig. (2-tailed)	.002	.000	.000
	N	116	116	116
(E5) Excitement-Seeking	Pearson Correlation	-.091	-.010	-.028
	Sig. (2-tailed)	.331	.918	.769
	N	116	116	116

Correlations

		(C6) Deliberation	MCAT Verbal	MCAT Biological Sciences
(N2) Angry Hostility	Pearson Correlation	-.192*	.107	.004
	Sig. (2-tailed)	.039	.254	.963
	N	116	116	116
(N3) Depression	Pearson Correlation	-.298**	.134	.061
	Sig. (2-tailed)	.001	.152	.517
	N	116	116	116
(N4) Self-Consciousness	Pearson Correlation	-.177	.130	.107
	Sig. (2-tailed)	.058	.163	.252
	N	116	116	116
(N5) Impulsiveness	Pearson Correlation	-.398**	.055	.004
	Sig. (2-tailed)	.000	.560	.970
	N	116	116	116
(N6) Vulnerability	Pearson Correlation	-.322**	.015	.015
	Sig. (2-tailed)	.000	.873	.872
	N	116	116	116
(E1) Warmth	Pearson Correlation	.197*	-.161	-.159
	Sig. (2-tailed)	.034	.084	.088
	N	116	116	116
(E2) Gregariousness	Pearson Correlation	.010	-.234*	-.177
	Sig. (2-tailed)	.915	.011	.058
	N	116	116	116
(E3) Assertiveness	Pearson Correlation	.111	-.219*	-.322**
	Sig. (2-tailed)	.235	.018	.000
	N	116	116	116
(E4) Activity	Pearson Correlation	.176	-.062	-.216*
	Sig. (2-tailed)	.058	.508	.020
	N	116	116	116
(E5) Excitement-Seeking	Pearson Correlation	-.122	-.090	-.027
	Sig. (2-tailed)	.193	.334	.772
	N	116	116	116

Correlations

		MCAT Physical Sciences	Yr 1 Phase A MDE	Yr 1 Phase B MDE
(N2) Angry Hostility	Pearson Correlation	.104	-.076	-.102
	Sig. (2-tailed)	.268	.421	.280
	N	116	115	115
(N3) Depression	Pearson Correlation	.077	-.049	-.126
	Sig. (2-tailed)	.413	.603	.181
	N	116	115	115
(N4) Self-Consciousness	Pearson Correlation	.099	.098	-.006
	Sig. (2-tailed)	.290	.297	.949
	N	116	115	115
(N5) Impulsiveness	Pearson Correlation	.082	.066	-.045
	Sig. (2-tailed)	.384	.481	.631
	N	116	115	115
(N6) Vulnerability	Pearson Correlation	.044	-.057	-.105
	Sig. (2-tailed)	.642	.547	.265
	N	116	115	115
(E1) Warmth	Pearson Correlation	.038	-.155	-.106
	Sig. (2-tailed)	.685	.097	.259
	N	116	115	115
(E2) Gregariousness	Pearson Correlation	-.100	-.149	-.061
	Sig. (2-tailed)	.286	.112	.518
	N	116	115	115
(E3) Assertiveness	Pearson Correlation	-.177	-.268**	-.224*
	Sig. (2-tailed)	.058	.004	.016
	N	116	115	115
(E4) Activity	Pearson Correlation	-.107	-.230*	-.169
	Sig. (2-tailed)	.254	.013	.072
	N	116	115	115
(E5) Excitement-Seeking	Pearson Correlation	.041	.041	.001
	Sig. (2-tailed)	.659	.667	.990
	N	116	115	115

Correlations

		Yr 1 Host Defense MDE	Yr 2 GI MDE	Yr 2 Renal MDE
(N2) Angry Hostility	Pearson Correlation	-.102	-.152	-.106
	Sig. (2-tailed)	.282	.319	.486
	N	113	45	45
(N3) Depression	Pearson Correlation	-.070	-.205	-.097
	Sig. (2-tailed)	.458	.178	.526
	N	113	45	45
(N4) Self-Consciousness	Pearson Correlation	.084	-.008	.017
	Sig. (2-tailed)	.378	.959	.910
	N	113	45	45
(N5) Impulsiveness	Pearson Correlation	.068	-.151	.036
	Sig. (2-tailed)	.476	.323	.815
	N	113	45	45
(N6) Vulnerability	Pearson Correlation	-.110	-.118	-.120
	Sig. (2-tailed)	.244	.441	.431
	N	113	45	45
(E1) Warmth	Pearson Correlation	-.053	.155	.086
	Sig. (2-tailed)	.576	.309	.576
	N	113	45	45
(E2) Gregariousness	Pearson Correlation	.030	.158	-.054
	Sig. (2-tailed)	.753	.300	.722
	N	113	45	45
(E3) Assertiveness	Pearson Correlation	-.144	-.058	-.027
	Sig. (2-tailed)	.128	.707	.858
	N	113	45	45
(E4) Activity	Pearson Correlation	-.099	.189	.082
	Sig. (2-tailed)	.298	.213	.594
	N	113	45	45
(E5) Excitement-Seeking	Pearson Correlation	.101	.052	.042
	Sig. (2-tailed)	.288	.735	.783
	N	113	45	45

Correlations

		Yr 2 Infectious Diseases MDE	Yr 1 Hematology MDE	Yr 1 Neurology MDE
(N2) Angry Hostility	Pearson Correlation	-.075	-.093	-.131
	Sig. (2-tailed)	.624	.337	.392
	N	45	109	45
(N3) Depression	Pearson Correlation	-.159	.010	-.293
	Sig. (2-tailed)	.297	.918	.050
	N	45	109	45
(N4) Self-Consciousness	Pearson Correlation	.077	.103	-.140
	Sig. (2-tailed)	.616	.287	.361
	N	45	109	45
(N5) Impulsiveness	Pearson Correlation	-.041	-.011	-.160
	Sig. (2-tailed)	.788	.908	.293
	N	45	109	45
(N6) Vulnerability	Pearson Correlation	-.035	-.163	-.235
	Sig. (2-tailed)	.818	.091	.120
	N	45	109	45
(E1) Warmth	Pearson Correlation	.019	-.001	.116
	Sig. (2-tailed)	.904	.989	.448
	N	45	109	45
(E2) Gregariousness	Pearson Correlation	.060	-.042	-.038
	Sig. (2-tailed)	.695	.663	.805
	N	45	109	45
(E3) Assertiveness	Pearson Correlation	-.112	.003	.242
	Sig. (2-tailed)	.464	.977	.110
	N	45	109	45
(E4) Activity	Pearson Correlation	.005	.085	.148
	Sig. (2-tailed)	.976	.379	.331
	N	45	109	45
(E5) Excitement-Seeking	Pearson Correlation	.045	.007	-.055
	Sig. (2-tailed)	.770	.946	.721
	N	45	109	45

Correlations

		Yr 1 Brain & Behavior MDE	Yr 1 Musculoskeletal MDE	Yr 1 Community Epidimiology Study Grade
(N2) Angry Hostility	Pearson Correlation	.009	-.211	.022
	Sig. (2-tailed)	.937	.163	.819
	N	77	45	115
(N3) Depression	Pearson Correlation	-.049	-.244	-.022
	Sig. (2-tailed)	.670	.107	.818
	N	77	45	115
(N4) Self-Consciousness	Pearson Correlation	.139	-.078	.081
	Sig. (2-tailed)	.227	.608	.387
	N	77	45	115
(N5) Impulsiveness	Pearson Correlation	.091	-.122	.070
	Sig. (2-tailed)	.432	.424	.457
	N	77	45	115
(N6) Vulnerability	Pearson Correlation	-.174	-.193	-.021
	Sig. (2-tailed)	.131	.203	.821
	N	77	45	115
(E1) Warmth	Pearson Correlation	-.025	.129	-.216*
	Sig. (2-tailed)	.827	.398	.021
	N	77	45	115
(E2) Gregariousness	Pearson Correlation	-.136	-.068	-.201*
	Sig. (2-tailed)	.238	.658	.031
	N	77	45	115
(E3) Assertiveness	Pearson Correlation	-.072	-.028	-.136
	Sig. (2-tailed)	.531	.855	.147
	N	77	45	115
(E4) Activity	Pearson Correlation	.017	.065	-.128
	Sig. (2-tailed)	.884	.672	.173
	N	77	45	115
(E5) Excitement-Seeking	Pearson Correlation	-.078	-.012	-.041
	Sig. (2-tailed)	.502	.940	.664
	N	77	45	115

Correlations

		Yr 2 Cardiology MDE	Yr 2 Biostatistics	Yr 2 Pulmonology MDE
(N2) Angry Hostility	Pearson Correlation	-.234	-.006	-.186
	Sig. (2-tailed)	.122	.967	.221
	N	45	45	45
(N3) Depression	Pearson Correlation	-.277	.051	-.205
	Sig. (2-tailed)	.066	.737	.178
	N	45	45	45
(N4) Self-Consciousness	Pearson Correlation	-.157	-.017	-.023
	Sig. (2-tailed)	.303	.910	.881
	N	45	45	45
(N5) Impulsiveness	Pearson Correlation	-.142	.083	-.044
	Sig. (2-tailed)	.353	.587	.776
	N	45	45	45
(N6) Vulnerability	Pearson Correlation	-.326*	.000	-.286
	Sig. (2-tailed)	.029	.999	.057
	N	45	45	45
(E1) Warmth	Pearson Correlation	.077	-.022	.036
	Sig. (2-tailed)	.617	.888	.814
	N	45	45	45
(E2) Gregariousness	Pearson Correlation	-.085	.002	-.108
	Sig. (2-tailed)	.581	.992	.480
	N	45	45	45
(E3) Assertiveness	Pearson Correlation	.065	.040	-.036
	Sig. (2-tailed)	.672	.792	.815
	N	45	45	45
(E4) Activity	Pearson Correlation	.139	.059	.112
	Sig. (2-tailed)	.361	.699	.465
	N	45	45	45
(E5) Excitement-Seeking	Pearson Correlation	-.081	-.023	-.047
	Sig. (2-tailed)	.596	.878	.757
	N	45	45	45

Correlations

		Yr 2 Endocrinology MDE	Yr 2 Research Design	Repeat Phase A MDE
(N2) Angry Hostility	Pearson Correlation	.003	.093	.151
	Sig. (2-tailed)	.982	.542	.698
	N	46	45	9
(N3) Depression	Pearson Correlation	.003	.060	.222
	Sig. (2-tailed)	.984	.694	.566
	N	46	45	9
(N4) Self-Consciousness	Pearson Correlation	.095	.030	.013
	Sig. (2-tailed)	.529	.843	.973
	N	46	45	9
(N5) Impulsiveness	Pearson Correlation	-.009	.061	.116
	Sig. (2-tailed)	.954	.690	.766
	N	46	45	9
(N6) Vulnerability	Pearson Correlation	-.091	-.149	.081
	Sig. (2-tailed)	.546	.329	.836
	N	46	45	9
(E1) Warmth	Pearson Correlation	.091	.063	-.038
	Sig. (2-tailed)	.547	.679	.922
	N	46	45	9
(E2) Gregariousness	Pearson Correlation	-.016	.007	-.014
	Sig. (2-tailed)	.913	.963	.971
	N	46	45	9
(E3) Assertiveness	Pearson Correlation	-.046	.109	-.396
	Sig. (2-tailed)	.759	.478	.291
	N	46	45	9
(E4) Activity	Pearson Correlation	.172	.144	.092
	Sig. (2-tailed)	.253	.345	.814
	N	46	45	9
(E5) Excitement-Seeking	Pearson Correlation	-.120	.044	-.243
	Sig. (2-tailed)	.425	.775	.528
	N	46	45	9

Correlations

		Repeat Phase B MDE	Repeat Phase A SAP	Repeat Host Defense MDE
(N2) Angry Hostility	Pearson Correlation	-.282	-.191	-.067
	Sig. (2-tailed)	.462	.622	.865
	N	9	9	9
(N3) Depression	Pearson Correlation	-.542	-.339	.053
	Sig. (2-tailed)	.132	.371	.893
	N	9	9	9
(N4) Self-Consciousness	Pearson Correlation	-.484	-.374	-.217
	Sig. (2-tailed)	.187	.321	.575
	N	9	9	9
(N5) Impulsiveness	Pearson Correlation	.304	-.353	.110
	Sig. (2-tailed)	.426	.352	.779
	N	9	9	9
(N6) Vulnerability	Pearson Correlation	-.113	-.575	-.013
	Sig. (2-tailed)	.773	.105	.973
	N	9	9	9
(E1) Warmth	Pearson Correlation	.021	.146	.092
	Sig. (2-tailed)	.957	.708	.814
	N	9	9	9
(E2) Gregariousness	Pearson Correlation	.532	.024	.207
	Sig. (2-tailed)	.141	.951	.592
	N	9	9	9
(E3) Assertiveness	Pearson Correlation	.293	.265	-.200
	Sig. (2-tailed)	.443	.491	.605
	N	9	9	9
(E4) Activity	Pearson Correlation	.458	.603	-.082
	Sig. (2-tailed)	.215	.086	.834
	N	9	9	9
(E5) Excitement-Seeking	Pearson Correlation	.894**	.094	.076
	Sig. (2-tailed)	.001	.811	.845
	N	9	9	9

Correlations

		Repeat Hematology MDE	Repeat Neurology MDE	Repeat Brain & Behavior MDE
(N2) Angry Hostility	Pearson Correlation	.098	.112	-.666
	Sig. (2-tailed)	.801	.774	.050
	N	9	9	9
(N3) Depression	Pearson Correlation	-.091	-.010	-.620
	Sig. (2-tailed)	.817	.979	.075
	N	9	9	9
(N4) Self-Consciousness	Pearson Correlation	.219	.181	-.574
	Sig. (2-tailed)	.572	.642	.106
	N	9	9	9
(N5) Impulsiveness	Pearson Correlation	.480	-.061	-.479
	Sig. (2-tailed)	.191	.877	.192
	N	9	9	9
(N6) Vulnerability	Pearson Correlation	.289	-.013	-.564
	Sig. (2-tailed)	.451	.974	.113
	N	9	9	9
(E1) Warmth	Pearson Correlation	-.024	-.192	.638
	Sig. (2-tailed)	.951	.621	.065
	N	9	9	9
(E2) Gregariousness	Pearson Correlation	.073	-.071	.263
	Sig. (2-tailed)	.853	.856	.494
	N	9	9	9
(E3) Assertiveness	Pearson Correlation	-.418	.277	.443
	Sig. (2-tailed)	.263	.470	.233
	N	9	9	9
(E4) Activity	Pearson Correlation	.238	.282	.223
	Sig. (2-tailed)	.538	.463	.565
	N	9	9	9
(E5) Excitement-Seeking	Pearson Correlation	.069	-.034	.393
	Sig. (2-tailed)	.860	.931	.296
	N	9	9	9

Correlations

		Repeat Musculoskeletal MDE
(N2) Angry Hostility	Pearson Correlation	-.481
	Sig. (2-tailed)	.190
	N	9
(N3) Depression	Pearson Correlation	-.162
	Sig. (2-tailed)	.677
	N	9
(N4) Self-Consciousness	Pearson Correlation	-.318
	Sig. (2-tailed)	.404
	N	9
(N5) Impulsiveness	Pearson Correlation	-.501
	Sig. (2-tailed)	.169
	N	9
(N6) Vulnerability	Pearson Correlation	-.412
	Sig. (2-tailed)	.271
	N	9
(E1) Warmth	Pearson Correlation	.623
	Sig. (2-tailed)	.073
	N	9
(E2) Gregariousness	Pearson Correlation	.438
	Sig. (2-tailed)	.238
	N	9
(E3) Assertiveness	Pearson Correlation	.097
	Sig. (2-tailed)	.805
	N	9
(E4) Activity	Pearson Correlation	.130
	Sig. (2-tailed)	.738
	N	9
(E5) Excitement-Seeking	Pearson Correlation	-.266
	Sig. (2-tailed)	.490
	N	9

Correlations

		(N) Neuroticism	(E) Extraversion	(O) Openness
(E6) Positive Emotions	Pearson Correlation	-.140	.736**	.254**
	Sig. (2-tailed)	.134	.000	.006
	N	116	116	116
(O1) Fantasy	Pearson Correlation	.141	.109	.583**
	Sig. (2-tailed)	.132	.245	.000
	N	116	116	116
(O2) Aesthetics	Pearson Correlation	.038	.093	.762**
	Sig. (2-tailed)	.687	.323	.000
	N	116	116	116
(O3) Feelings	Pearson Correlation	.403**	.375**	.429**
	Sig. (2-tailed)	.000	.000	.000
	N	116	116	116
(O4) Actions	Pearson Correlation	-.382**	.138	.524**
	Sig. (2-tailed)	.000	.141	.000
	N	116	116	116
(O5) Ideas	Pearson Correlation	-.119	.008	.787**
	Sig. (2-tailed)	.204	.935	.000
	N	116	116	116
(O6) Values	Pearson Correlation	-.144	-.075	.502**
	Sig. (2-tailed)	.124	.424	.000
	N	116	116	116
(A1) Trust	Pearson Correlation	-.416**	.349**	.135
	Sig. (2-tailed)	.000	.000	.149
	N	116	116	116
(A2) Straightforwardness	Pearson Correlation	-.163	.169	-.176
	Sig. (2-tailed)	.080	.070	.058
	N	116	116	116
(A3) Altruism	Pearson Correlation	-.283**	.587**	.032
	Sig. (2-tailed)	.002	.000	.732
	N	116	116	116

Correlations

		(A) Agreeableness	(C) Conscientiousness	(N1) Anxiety
(E6) Positive Emotions	Pearson Correlation	.415**	.067	-.090
	Sig. (2-tailed)	.000	.472	.339
	N	116	116	116
(O1) Fantasy	Pearson Correlation	-.257**	-.357**	.075
	Sig. (2-tailed)	.005	.000	.426
	N	116	116	116
(O2) Aesthetics	Pearson Correlation	.112	.019	-.023
	Sig. (2-tailed)	.231	.836	.809
	N	116	116	116
(O3) Feelings	Pearson Correlation	.063	-.016	.290**
	Sig. (2-tailed)	.499	.867	.002
	N	116	116	116
(O4) Actions	Pearson Correlation	.081	-.183*	-.340**
	Sig. (2-tailed)	.385	.049	.000
	N	116	116	116
(O5) Ideas	Pearson Correlation	.092	.166	-.085
	Sig. (2-tailed)	.327	.075	.367
	N	116	116	116
(O6) Values	Pearson Correlation	.051	-.164	-.053
	Sig. (2-tailed)	.586	.079	.574
	N	116	116	116
(A1) Trust	Pearson Correlation	.684**	.143	-.244**
	Sig. (2-tailed)	.000	.126	.008
	N	116	116	116
(A2) Straightforwardness	Pearson Correlation	.789**	.438**	-.071
	Sig. (2-tailed)	.000	.000	.447
	N	116	116	116
(A3) Altruism	Pearson Correlation	.751**	.367**	-.158
	Sig. (2-tailed)	.000	.000	.090
	N	116	116	116

Correlations

		(N2) Angry Hostility	(N3) Depression	(N4) Self-Consciousness
(E6) Positive Emotions	Pearson Correlation	-.325**	-.290**	-.262**
	Sig. (2-tailed)	.000	.002	.004
	N	116	116	116
(O1) Fantasy	Pearson Correlation	.233*	.237*	.042
	Sig. (2-tailed)	.012	.011	.652
	N	116	116	116
(O2) Aesthetics	Pearson Correlation	-.096	-.019	-.037
	Sig. (2-tailed)	.305	.836	.695
	N	116	116	116
(O3) Feelings	Pearson Correlation	.116	.218*	.049
	Sig. (2-tailed)	.215	.019	.599
	N	116	116	116
(O4) Actions	Pearson Correlation	-.187*	-.241**	-.267**
	Sig. (2-tailed)	.044	.009	.004
	N	116	116	116
(O5) Ideas	Pearson Correlation	-.082	-.140	-.207*
	Sig. (2-tailed)	.380	.133	.026
	N	116	116	116
(O6) Values	Pearson Correlation	-.078	-.071	-.066
	Sig. (2-tailed)	.404	.447	.483
	N	116	116	116
(A1) Trust	Pearson Correlation	-.594**	-.510**	-.337**
	Sig. (2-tailed)	.000	.000	.000
	N	116	116	116
(A2) Straightforwardness	Pearson Correlation	-.519**	-.401**	-.241**
	Sig. (2-tailed)	.000	.000	.009
	N	116	116	116
(A3) Altruism	Pearson Correlation	-.559**	-.516**	-.393**
	Sig. (2-tailed)	.000	.000	.000
	N	116	116	116

Correlations

		(N5) Impulsiveness	(N6) Vulnerability	(E1) Warmth
(E6) Positive Emotions	Pearson Correlation	-.027	-.306**	.555**
	Sig. (2-tailed)	.775	.001	.000
	N	116	116	116
(O1) Fantasy	Pearson Correlation	.313**	.116	-.055
	Sig. (2-tailed)	.001	.215	.559
	N	116	116	116
(O2) Aesthetics	Pearson Correlation	.002	-.097	.088
	Sig. (2-tailed)	.984	.300	.348
	N	116	116	116
(O3) Feelings	Pearson Correlation	.235*	.169	.163
	Sig. (2-tailed)	.011	.069	.080
	N	116	116	116
(O4) Actions	Pearson Correlation	-.108	-.259**	.048
	Sig. (2-tailed)	.247	.005	.611
	N	116	116	116
(O5) Ideas	Pearson Correlation	-.011	-.343**	.085
	Sig. (2-tailed)	.907	.000	.366
	N	116	116	116
(O6) Values	Pearson Correlation	.028	-.099	-.080
	Sig. (2-tailed)	.766	.292	.395
	N	116	116	116
(A1) Trust	Pearson Correlation	-.244**	-.327**	.460**
	Sig. (2-tailed)	.008	.000	.000
	N	116	116	116
(A2) Straightforwardness	Pearson Correlation	-.383**	-.223*	.403**
	Sig. (2-tailed)	.000	.016	.000
	N	116	116	116
(A3) Altruism	Pearson Correlation	-.252**	-.431**	.707**
	Sig. (2-tailed)	.006	.000	.000
	N	116	116	116

Correlations

		(E2) Gregariousness	(E3) Assertiveness	(E4) Activity
(E6) Positive Emotions	Pearson Correlation	.425**	.232*	.355**
	Sig. (2-tailed)	.000	.012	.000
	N	116	116	116
(O1) Fantasy	Pearson Correlation	.047	.022	-.061
	Sig. (2-tailed)	.619	.812	.515
	N	116	116	116
(O2) Aesthetics	Pearson Correlation	.168	.046	.126
	Sig. (2-tailed)	.072	.625	.178
	N	116	116	116
(O3) Feelings	Pearson Correlation	.236*	.061	.157
	Sig. (2-tailed)	.011	.514	.093
	N	116	116	116
(O4) Actions	Pearson Correlation	.186*	.058	.153
	Sig. (2-tailed)	.046	.534	.102
	N	116	116	116
(O5) Ideas	Pearson Correlation	.031	.154	.140
	Sig. (2-tailed)	.744	.099	.133
	N	116	116	116
(O6) Values	Pearson Correlation	-.028	-.005	-.058
	Sig. (2-tailed)	.764	.960	.537
	N	116	116	116
(A1) Trust	Pearson Correlation	.217*	.062	.258**
	Sig. (2-tailed)	.019	.506	.005
	N	116	116	116
(A2) Straightforwardness	Pearson Correlation	.136	-.034	.125
	Sig. (2-tailed)	.145	.720	.183
	N	116	116	116
(A3) Altruism	Pearson Correlation	.402**	.192*	.352**
	Sig. (2-tailed)	.000	.039	.000
	N	116	116	116

Correlations

		(E5) Excitement-Seeking	(E6) Positive Emotions	(O1) Fantasy
(E6) Positive Emotions	Pearson Correlation	.192*	1	.246**
	Sig. (2-tailed)	.039		.008
	N	116	116	116
(O1) Fantasy	Pearson Correlation	.214*	.246**	1
	Sig. (2-tailed)	.021	.008	
	N	116	116	116
(O2) Aesthetics	Pearson Correlation	.168	.295**	.312**
	Sig. (2-tailed)	.071	.001	.001
	N	116	116	116
(O3) Feelings	Pearson Correlation	.202*	.437**	.366**
	Sig. (2-tailed)	.029	.000	.000
	N	116	116	116
(O4) Actions	Pearson Correlation	.224*	.228*	.196*
	Sig. (2-tailed)	.016	.014	.035
	N	116	116	116
(O5) Ideas	Pearson Correlation	.177	.225*	.357**
	Sig. (2-tailed)	.057	.015	.000
	N	116	116	116
(O6) Values	Pearson Correlation	-.049	.059	.074
	Sig. (2-tailed)	.599	.526	.432
	N	116	116	116
(A1) Trust	Pearson Correlation	-.113	.415**	-.090
	Sig. (2-tailed)	.227	.000	.336
	N	116	116	116
(A2) Straightforwardness	Pearson Correlation	-.199*	.230*	-.389**
	Sig. (2-tailed)	.033	.013	.000
	N	116	116	116
(A3) Altruism	Pearson Correlation	.033	.506**	-.146
	Sig. (2-tailed)	.721	.000	.117
	N	116	116	116

Correlations

		(O2) Aesthetics	(O3) Feelings	(O4) Actions
(E6) Positive Emotions	Pearson Correlation	.295**	.437**	.228*
	Sig. (2-tailed)	.001	.000	.014
	N	116	116	116
(O1) Fantasy	Pearson Correlation	.312**	.366**	.196*
	Sig. (2-tailed)	.001	.000	.035
	N	116	116	116
(O2) Aesthetics	Pearson Correlation	1	.426**	.280**
	Sig. (2-tailed)		.000	.002
	N	116	116	116
(O3) Feelings	Pearson Correlation	.426**	1	.010
	Sig. (2-tailed)	.000		.918
	N	116	116	116
(O4) Actions	Pearson Correlation	.280**	.010	1
	Sig. (2-tailed)	.002	.918	
	N	116	116	116
(O5) Ideas	Pearson Correlation	.499**	.277**	.354**
	Sig. (2-tailed)	.000	.003	.000
	N	116	116	116
(O6) Values	Pearson Correlation	.330**	.101	.179
	Sig. (2-tailed)	.000	.279	.055
	N	116	116	116
(A1) Trust	Pearson Correlation	.121	.028	.115
	Sig. (2-tailed)	.196	.762	.218
	N	116	116	116
(A2) Straightforwardness	Pearson Correlation	-.069	-.033	-.019
	Sig. (2-tailed)	.465	.721	.842
	N	116	116	116
(A3) Altruism	Pearson Correlation	.150	.133	.075
	Sig. (2-tailed)	.109	.153	.426
	N	116	116	116

Correlations

		(O5) Ideas	(O6) Values	(A1) Trust	(A2) Straightforwardness
(E6) Positive Emotions	Pearson Correlation	.225*	.059	.415**	.230*
	Sig. (2-tailed)	.015	.526	.000	.013
	N	116	116	116	116
(O1) Fantasy	Pearson Correlation	.357**	.074	-.090	-.389**
	Sig. (2-tailed)	.000	.432	.336	.000
	N	116	116	116	116
(O2) Aesthetics	Pearson Correlation	.499**	.330**	.121	-.069
	Sig. (2-tailed)	.000	.000	.196	.465
	N	116	116	116	116
(O3) Feelings	Pearson Correlation	.277**	.101	.028	-.033
	Sig. (2-tailed)	.003	.279	.762	.721
	N	116	116	116	116
(O4) Actions	Pearson Correlation	.354**	.179	.115	-.019
	Sig. (2-tailed)	.000	.055	.218	.842
	N	116	116	116	116
(O5) Ideas	Pearson Correlation	1	.250**	.174	-.005
	Sig. (2-tailed)		.007	.062	.958
	N	116	116	116	116
(O6) Values	Pearson Correlation	.250**	1	.164	-.076
	Sig. (2-tailed)	.007		.078	.419
	N	116	116	116	116
(A1) Trust	Pearson Correlation	.174	.164	1	.491**
	Sig. (2-tailed)	.062	.078		.000
	N	116	116	116	116
(A2) Straightforwardness	Pearson Correlation	-.005	-.076	.491**	1
	Sig. (2-tailed)	.958	.419	.000	
	N	116	116	116	116
(A3) Altruism	Pearson Correlation	.215*	.032	.539**	.578**
	Sig. (2-tailed)	.021	.730	.000	.000
	N	116	116	116	116

Correlations

		(A3) Altruism	(A4) Compliance	(A5) Modesty
(E6) Positive Emotions	Pearson Correlation	.506**	.242**	.034
	Sig. (2-tailed)	.000	.009	.716
	N	116	116	116
(O1) Fantasy	Pearson Correlation	-.146	-.257**	-.141
	Sig. (2-tailed)	.117	.005	.132
	N	116	116	116
(O2) Aesthetics	Pearson Correlation	.150	.094	-.156
	Sig. (2-tailed)	.109	.315	.094
	N	116	116	116
(O3) Feelings	Pearson Correlation	.133	-.112	.046
	Sig. (2-tailed)	.153	.230	.626
	N	116	116	116
(O4) Actions	Pearson Correlation	.075	.038	.010
	Sig. (2-tailed)	.426	.686	.912
	N	116	116	116
(O5) Ideas	Pearson Correlation	.215*	.100	-.029
	Sig. (2-tailed)	.021	.283	.758
	N	116	116	116
(O6) Values	Pearson Correlation	.032	.069	-.069
	Sig. (2-tailed)	.730	.464	.461
	N	116	116	116
(A1) Trust	Pearson Correlation	.539**	.528**	.098
	Sig. (2-tailed)	.000	.000	.293
	N	116	116	116
(A2) Straightforwardness	Pearson Correlation	.578**	.597**	.327**
	Sig. (2-tailed)	.000	.000	.000
	N	116	116	116
(A3) Altruism	Pearson Correlation	1	.518**	.160
	Sig. (2-tailed)		.000	.086
	N	116	116	116

Correlations

		(A6) Tender-Mindedness	(C1) Competence	(C2) Order
(E6) Positive Emotions	Pearson Correlation	.490**	.267**	.067
	Sig. (2-tailed)	.000	.004	.475
	N	116	116	116
(O1) Fantasy	Pearson Correlation	-.116	-.112	-.294**
	Sig. (2-tailed)	.214	.232	.001
	N	116	116	116
(O2) Aesthetics	Pearson Correlation	.148	.058	-.039
	Sig. (2-tailed)	.113	.538	.677
	N	116	116	116
(O3) Feelings	Pearson Correlation	.202*	-.018	.013
	Sig. (2-tailed)	.029	.850	.890
	N	116	116	116
(O4) Actions	Pearson Correlation	.252**	.060	-.229*
	Sig. (2-tailed)	.006	.523	.014
	N	116	116	116
(O5) Ideas	Pearson Correlation	.161	.227*	-.042
	Sig. (2-tailed)	.084	.014	.653
	N	116	116	116
(O6) Values	Pearson Correlation	.105	-.112	-.073
	Sig. (2-tailed)	.263	.230	.439
	N	116	116	116
(A1) Trust	Pearson Correlation	.460**	.315**	.148
	Sig. (2-tailed)	.000	.001	.112
	N	116	116	116
(A2) Straightforwardness	Pearson Correlation	.483**	.352**	.358**
	Sig. (2-tailed)	.000	.000	.000
	N	116	116	116
(A3) Altruism	Pearson Correlation	.611**	.521**	.217*
	Sig. (2-tailed)	.000	.000	.019
	N	116	116	116

Correlations

		(C3) Dutifulness	(C4) Achievement Striving	(C5) Self-Discipline
(E6) Positive Emotions	Pearson Correlation	.204*	.076	.230*
	Sig. (2-tailed)	.028	.418	.013
	N	116	116	116
(O1) Fantasy	Pearson Correlation	-.358**	-.222*	-.253**
	Sig. (2-tailed)	.000	.017	.006
	N	116	116	116
(O2) Aesthetics	Pearson Correlation	-.071	.081	.078
	Sig. (2-tailed)	.447	.388	.403
	N	116	116	116
(O3) Feelings	Pearson Correlation	-.169	.048	-.137
	Sig. (2-tailed)	.069	.611	.142
	N	116	116	116
(O4) Actions	Pearson Correlation	-.008	-.047	.080
	Sig. (2-tailed)	.930	.618	.393
	N	116	116	116
(O5) Ideas	Pearson Correlation	.185*	.224*	.199*
	Sig. (2-tailed)	.047	.016	.032
	N	116	116	116
(O6) Values	Pearson Correlation	-.079	-.038	-.005
	Sig. (2-tailed)	.398	.683	.954
	N	116	116	116
(A1) Trust	Pearson Correlation	.324**	.105	.363**
	Sig. (2-tailed)	.000	.261	.000
	N	116	116	116
(A2) Straightforwardness	Pearson Correlation	.518**	.289**	.406**
	Sig. (2-tailed)	.000	.002	.000
	N	116	116	116
(A3) Altruism	Pearson Correlation	.519**	.253**	.388**
	Sig. (2-tailed)	.000	.006	.000
	N	116	116	116

Correlations

		(C6) Deliberation	MCAT Verbal	MCAT Biological Sciences
(E6) Positive Emotions	Pearson Correlation	-.119	-.183*	-.003
	Sig. (2-tailed)	.202	.049	.975
	N	116	116	116
(O1) Fantasy	Pearson Correlation	-.279**	.279**	.023
	Sig. (2-tailed)	.002	.002	.810
	N	116	116	116
(O2) Aesthetics	Pearson Correlation	-.126	.026	.195*
	Sig. (2-tailed)	.177	.785	.036
	N	116	116	116
(O3) Feelings	Pearson Correlation	-.218*	.093	.036
	Sig. (2-tailed)	.019	.322	.701
	N	116	116	116
(O4) Actions	Pearson Correlation	-.133	-.024	.146
	Sig. (2-tailed)	.154	.798	.118
	N	116	116	116
(O5) Ideas	Pearson Correlation	.073	.034	.141
	Sig. (2-tailed)	.436	.720	.130
	N	116	116	116
(O6) Values	Pearson Correlation	-.174	.070	.116
	Sig. (2-tailed)	.062	.455	.216
	N	116	116	116
(A1) Trust	Pearson Correlation	.166	.031	-.012
	Sig. (2-tailed)	.075	.739	.901
	N	116	116	116
(A2) Straightforwardness	Pearson Correlation	.355**	-.189*	-.033
	Sig. (2-tailed)	.000	.042	.727
	N	116	116	116
(A3) Altruism	Pearson Correlation	.340**	-.269**	-.122
	Sig. (2-tailed)	.000	.003	.192
	N	116	116	116

Correlations

		MCAT Physical Sciences	Yr 1 Phase A MDE	Yr 1 Phase B MDE
(E6) Positive Emotions	Pearson Correlation	.005	-.087	-.006
	Sig. (2-tailed)	.961	.356	.949
	N	116	115	115
(O1) Fantasy	Pearson Correlation	.140	-.089	-.078
	Sig. (2-tailed)	.134	.345	.407
	N	116	115	115
(O2) Aesthetics	Pearson Correlation	.068	-.106	-.065
	Sig. (2-tailed)	.470	.259	.489
	N	116	115	115
(O3) Feelings	Pearson Correlation	.004	-.201*	-.182
	Sig. (2-tailed)	.962	.032	.051
	N	116	115	115
(O4) Actions	Pearson Correlation	.155	.002	.003
	Sig. (2-tailed)	.097	.982	.977
	N	116	115	115
(O5) Ideas	Pearson Correlation	.149	.018	-.026
	Sig. (2-tailed)	.111	.849	.781
	N	116	115	115
(O6) Values	Pearson Correlation	.077	.058	.011
	Sig. (2-tailed)	.410	.540	.906
	N	116	115	115
(A1) Trust	Pearson Correlation	-.065	-.070	.039
	Sig. (2-tailed)	.486	.456	.680
	N	116	115	115
(A2) Straightforwardness	Pearson Correlation	-.126	.030	.048
	Sig. (2-tailed)	.179	.754	.611
	N	116	115	115
(A3) Altruism	Pearson Correlation	-.017	-.065	-.101
	Sig. (2-tailed)	.856	.490	.284
	N	116	115	115

Correlations

		Yr 1 Host Defense MDE	Yr 2 GI MDE	Yr 2 Renal MDE
(E6) Positive Emotions	Pearson Correlation	-.013	.426**	.239
	Sig. (2-tailed)	.891	.004	.114
	N	113	45	45
(O1) Fantasy	Pearson Correlation	-.104	-.045	-.157
	Sig. (2-tailed)	.274	.767	.302
	N	113	45	45
(O2) Aesthetics	Pearson Correlation	-.054	.094	-.146
	Sig. (2-tailed)	.570	.539	.338
	N	113	45	45
(O3) Feelings	Pearson Correlation	-.153	-.035	-.177
	Sig. (2-tailed)	.105	.820	.245
	N	113	45	45
(O4) Actions	Pearson Correlation	-.039	.037	.009
	Sig. (2-tailed)	.684	.811	.955
	N	113	45	45
(O5) Ideas	Pearson Correlation	-.036	.099	-.070
	Sig. (2-tailed)	.703	.517	.650
	N	113	45	45
(O6) Values	Pearson Correlation	-.102	-.145	-.076
	Sig. (2-tailed)	.283	.340	.620
	N	113	45	45
(A1) Trust	Pearson Correlation	-.038	.069	-.023
	Sig. (2-tailed)	.686	.653	.879
	N	113	45	45
(A2) Straightforwardness	Pearson Correlation	.057	.133	.025
	Sig. (2-tailed)	.551	.384	.868
	N	113	45	45
(A3) Altruism	Pearson Correlation	.036	.169	-.001
	Sig. (2-tailed)	.703	.268	.994
	N	113	45	45

Correlations

		Yr 2 Infectious Diseases MDE	Yr 1 Hematology MDE	Yr 1 Neurology MDE
(E6) Positive Emotions	Pearson Correlation	.139	.024	.233
	Sig. (2-tailed)	.363	.803	.123
	N	45	109	45
(O1) Fantasy	Pearson Correlation	-.355*	-.034	-.325*
	Sig. (2-tailed)	.017	.725	.029
	N	45	109	45
(O2) Aesthetics	Pearson Correlation	-.046	.076	-.066
	Sig. (2-tailed)	.763	.430	.666
	N	45	109	45
(O3) Feelings	Pearson Correlation	-.201	-.161	-.143
	Sig. (2-tailed)	.186	.094	.349
	N	45	109	45
(O4) Actions	Pearson Correlation	-.140	-.035	-.083
	Sig. (2-tailed)	.360	.719	.587
	N	45	109	45
(O5) Ideas	Pearson Correlation	-.109	.006	.017
	Sig. (2-tailed)	.477	.951	.912
	N	45	109	45
(O6) Values	Pearson Correlation	-.115	-.057	.074
	Sig. (2-tailed)	.451	.557	.631
	N	45	109	45
(A1) Trust	Pearson Correlation	.022	-.081	-.021
	Sig. (2-tailed)	.886	.405	.892
	N	45	109	45
(A2) Straightforwardness	Pearson Correlation	.065	-.003	.118
	Sig. (2-tailed)	.670	.973	.439
	N	45	109	45
(A3) Altruism	Pearson Correlation	.132	.109	.139
	Sig. (2-tailed)	.386	.261	.363
	N	45	109	45

Correlations

		Yr 1 Brain & Behavior MDE	Yr 1 Musculoskeletal MDE	Yr 1 Community Epidimiology Study Grade
(E6) Positive Emotions	Pearson Correlation	.093	.284	-.097
	Sig. (2-tailed)	.420	.058	.302
	N	77	45	115
(O1) Fantasy	Pearson Correlation	-.048	-.190	-.057
	Sig. (2-tailed)	.676	.212	.548
	N	77	45	115
(O2) Aesthetics	Pearson Correlation	.027	.016	.000
	Sig. (2-tailed)	.816	.915	.998
	N	77	45	115
(O3) Feelings	Pearson Correlation	-.204	-.102	-.091
	Sig. (2-tailed)	.075	.507	.332
	N	77	45	115
(O4) Actions	Pearson Correlation	-.077	-.152	.022
	Sig. (2-tailed)	.506	.318	.819
	N	77	45	115
(O5) Ideas	Pearson Correlation	-.011	.145	-.087
	Sig. (2-tailed)	.927	.341	.353
	N	77	45	115
(O6) Values	Pearson Correlation	.083	.093	.067
	Sig. (2-tailed)	.474	.545	.474
	N	77	45	115
(A1) Trust	Pearson Correlation	.008	.117	-.109
	Sig. (2-tailed)	.944	.445	.247
	N	77	45	115
(A2) Straightforwardness	Pearson Correlation	.076	.109	-.155
	Sig. (2-tailed)	.511	.478	.098
	N	77	45	115
(A3) Altruism	Pearson Correlation	-.014	.178	-.196*
	Sig. (2-tailed)	.906	.243	.036
	N	77	45	115

Correlations

		Yr 2 Cardiology MDE	Yr 2 Biostatistics	Yr 2 Pulmonology MDE
(E6) Positive Emotions	Pearson Correlation	.135	.050	.261
	Sig. (2-tailed)	.377	.743	.083
	N	45	45	45
(O1) Fantasy	Pearson Correlation	-.183	-.161	-.127
	Sig. (2-tailed)	.229	.291	.404
	N	45	45	45
(O2) Aesthetics	Pearson Correlation	-.087	.119	.094
	Sig. (2-tailed)	.568	.438	.541
	N	45	45	45
(O3) Feelings	Pearson Correlation	-.181	.151	-.123
	Sig. (2-tailed)	.235	.322	.420
	N	45	45	45
(O4) Actions	Pearson Correlation	-.171	.145	.084
	Sig. (2-tailed)	.262	.343	.584
	N	45	45	45
(O5) Ideas	Pearson Correlation	.064	.091	.110
	Sig. (2-tailed)	.675	.553	.472
	N	45	45	45
(O6) Values	Pearson Correlation	.120	.254	.128
	Sig. (2-tailed)	.432	.092	.401
	N	45	45	45
(A1) Trust	Pearson Correlation	.044	-.199	.059
	Sig. (2-tailed)	.773	.189	.699
	N	45	45	45
(A2) Straightforwardness	Pearson Correlation	.059	-.064	.034
	Sig. (2-tailed)	.700	.677	.826
	N	45	45	45
(A3) Altruism	Pearson Correlation	.100	-.044	.042
	Sig. (2-tailed)	.514	.776	.785
	N	45	45	45

Correlations

		Yr 2 Endocrinology MDE	Yr 2 Research Design	Repeat Phase A MDE
(E6) Positive Emotions	Pearson Correlation	.167	.070	.059
	Sig. (2-tailed)	.267	.648	.881
	N	46	45	9
(O1) Fantasy	Pearson Correlation	-.168	-.165	-.039
	Sig. (2-tailed)	.264	.277	.920
	N	46	45	9
(O2) Aesthetics	Pearson Correlation	-.073	-.022	-.553
	Sig. (2-tailed)	.628	.884	.123
	N	46	45	9
(O3) Feelings	Pearson Correlation	-.263	.239	-.432
	Sig. (2-tailed)	.077	.113	.246
	N	46	45	9
(O4) Actions	Pearson Correlation	-.034	.076	-.051
	Sig. (2-tailed)	.822	.621	.896
	N	46	45	9
(O5) Ideas	Pearson Correlation	-.157	.064	-.757*
	Sig. (2-tailed)	.297	.677	.018
	N	46	45	9
(O6) Values	Pearson Correlation	-.121	.211	-.722*
	Sig. (2-tailed)	.422	.164	.028
	N	46	45	9
(A1) Trust	Pearson Correlation	-.019	-.191	-.248
	Sig. (2-tailed)	.902	.209	.520
	N	46	45	9
(A2) Straightforwardness	Pearson Correlation	.050	-.044	-.159
	Sig. (2-tailed)	.742	.775	.683
	N	46	45	9
(A3) Altruism	Pearson Correlation	-.031	-.049	-.055
	Sig. (2-tailed)	.838	.751	.888
	N	46	45	9

Correlations

		Repeat Phase B MDE	Repeat Phase A SAP	Repeat Host Defense MDE
(E6) Positive Emotions	Pearson Correlation	-.230	.145	-.386
	Sig. (2-tailed)	.552	.710	.305
	N	9	9	9
(O1) Fantasy	Pearson Correlation	-.210	-.050	-.416
	Sig. (2-tailed)	.587	.899	.265
	N	9	9	9
(O2) Aesthetics	Pearson Correlation	.265	-.010	-.710*
	Sig. (2-tailed)	.490	.980	.032
	N	9	9	9
(O3) Feelings	Pearson Correlation	-.165	-.220	-.686*
	Sig. (2-tailed)	.670	.570	.041
	N	9	9	9
(O4) Actions	Pearson Correlation	.274	.527	-.384
	Sig. (2-tailed)	.476	.145	.307
	N	9	9	9
(O5) Ideas	Pearson Correlation	.398	-.097	-.533
	Sig. (2-tailed)	.289	.803	.140
	N	9	9	9
(O6) Values	Pearson Correlation	.303	-.281	-.544
	Sig. (2-tailed)	.428	.464	.130
	N	9	9	9
(A1) Trust	Pearson Correlation	.473	-.207	.113
	Sig. (2-tailed)	.198	.592	.773
	N	9	9	9
(A2) Straightforwardness	Pearson Correlation	.233	.188	-.100
	Sig. (2-tailed)	.546	.628	.798
	N	9	9	9
(A3) Altruism	Pearson Correlation	.058	-.016	.176
	Sig. (2-tailed)	.882	.968	.651
	N	9	9	9

Correlations

		Repeat Hematology MDE	Repeat Neurology MDE	Repeat Brain & Behavior MDE
(E6) Positive Emotions	Pearson Correlation	.467	.295	.299
	Sig. (2-tailed)	.205	.441	.434
	N	9	9	9
(O1) Fantasy	Pearson Correlation	.418	.331	-.116
	Sig. (2-tailed)	.263	.385	.766
	N	9	9	9
(O2) Aesthetics	Pearson Correlation	.412	.682*	.176
	Sig. (2-tailed)	.271	.043	.652
	N	9	9	9
(O3) Feelings	Pearson Correlation	.218	.637	-.291
	Sig. (2-tailed)	.574	.065	.447
	N	9	9	9
(O4) Actions	Pearson Correlation	.669*	.437	.025
	Sig. (2-tailed)	.049	.240	.950
	N	9	9	9
(O5) Ideas	Pearson Correlation	.313	.458	.307
	Sig. (2-tailed)	.412	.215	.421
	N	9	9	9
(O6) Values	Pearson Correlation	.518	.559	-.290
	Sig. (2-tailed)	.153	.118	.448
	N	9	9	9
(A1) Trust	Pearson Correlation	.169	-.038	.351
	Sig. (2-tailed)	.664	.922	.355
	N	9	9	9
(A2) Straightforwardness	Pearson Correlation	.371	-.053	.651
	Sig. (2-tailed)	.325	.892	.057
	N	9	9	9
(A3) Altruism	Pearson Correlation	.138	-.250	.467
	Sig. (2-tailed)	.723	.517	.205
	N	9	9	9

Correlations

		Repeat Musculoskeletal MDE
(E6) Positive Emotions	Pearson Correlation	.161
	Sig. (2-tailed)	.679
	N	9
(O1) Fantasy	Pearson Correlation	-.486
	Sig. (2-tailed)	.184
	N	9
(O2) Aesthetics	Pearson Correlation	-.410
	Sig. (2-tailed)	.273
	N	9
(O3) Feelings	Pearson Correlation	-.542
	Sig. (2-tailed)	.132
	N	9
(O4) Actions	Pearson Correlation	-.166
	Sig. (2-tailed)	.669
	N	9
(O5) Ideas	Pearson Correlation	-.250
	Sig. (2-tailed)	.517
	N	9
(O6) Values	Pearson Correlation	-.195
	Sig. (2-tailed)	.615
	N	9
(A1) Trust	Pearson Correlation	.468
	Sig. (2-tailed)	.203
	N	9
(A2) Straightforwardness	Pearson Correlation	.320
	Sig. (2-tailed)	.401
	N	9
(A3) Altruism	Pearson Correlation	.670*
	Sig. (2-tailed)	.049
	N	9

Correlations

		(N) Neuroticism	(E) Extraversion	(O) Openness
(A4) Compliance	Pearson Correlation	-.247**	.134	.007
	Sig. (2-tailed)	.007	.151	.944
	N	116	116	116
(A5) Modesty	Pearson Correlation	.131	.014	-.118
	Sig. (2-tailed)	.160	.881	.208
	N	116	116	116
(A6) Tender-Mindedness	Pearson Correlation	-.123	.507**	.132
	Sig. (2-tailed)	.188	.000	.159
	N	116	116	116
(C1) Competence	Pearson Correlation	-.409**	.248**	.033
	Sig. (2-tailed)	.000	.007	.722
	N	116	116	116
(C2) Order	Pearson Correlation	.124	.086	-.240**
	Sig. (2-tailed)	.186	.360	.009
	N	116	116	116
(C3) Dutifulness	Pearson Correlation	-.388**	.109	-.093
	Sig. (2-tailed)	.000	.246	.318
	N	116	116	116
(C4) Achievement Striving	Pearson Correlation	.013	.128	.024
	Sig. (2-tailed)	.887	.172	.794
	N	116	116	116
(C5) Self-Discipline	Pearson Correlation	-.436**	.163	.002
	Sig. (2-tailed)	.000	.080	.981
	N	116	116	116
(C6) Deliberation	Pearson Correlation	-.138	-.150	-.149
	Sig. (2-tailed)	.140	.109	.110
	N	116	116	116
MCAT Verbal	Pearson Correlation	.013	-.223*	.160
	Sig. (2-tailed)	.887	.016	.086
	N	116	116	116

Correlations

		(A) Agreeableness	(C) Conscientiousness	(N1) Anxiety
(A4) Compliance	Pearson Correlation	.806**	.233*	-.126
	Sig. (2-tailed)	.000	.012	.177
	N	116	116	116
(A5) Modesty	Pearson Correlation	.481**	-.022	.005
	Sig. (2-tailed)	.000	.813	.960
	N	116	116	116
(A6) Tender-Mindedness	Pearson Correlation	.670**	.222*	-.080
	Sig. (2-tailed)	.000	.017	.392
	N	116	116	116
(C1) Competence	Pearson Correlation	.287**	.705**	-.286**
	Sig. (2-tailed)	.002	.000	.002
	N	116	116	116
(C2) Order	Pearson Correlation	.181	.737**	.213*
	Sig. (2-tailed)	.051	.000	.022
	N	116	116	116
(C3) Dutifulness	Pearson Correlation	.454**	.748**	-.264**
	Sig. (2-tailed)	.000	.000	.004
	N	116	116	116
(C4) Achievement Striving	Pearson Correlation	.110	.807**	.086
	Sig. (2-tailed)	.240	.000	.357
	N	116	116	116
(C5) Self-Discipline	Pearson Correlation	.308**	.769**	-.255**
	Sig. (2-tailed)	.001	.000	.006
	N	116	116	116
(C6) Deliberation	Pearson Correlation	.292**	.652**	-.079
	Sig. (2-tailed)	.001	.000	.400
	N	116	116	116
MCAT Verbal	Pearson Correlation	-.143	-.229*	-.064
	Sig. (2-tailed)	.127	.014	.496
	N	116	116	116

Correlations

		(N2) Angry Hostility	(N3) Depression	(N4) Self-Consciousness
(A4) Compliance	Pearson Correlation	-.612**	-.302**	-.131
	Sig. (2-tailed)	.000	.001	.162
	N	116	116	116
(A5) Modesty	Pearson Correlation	-.176	.076	.177
	Sig. (2-tailed)	.059	.419	.057
	N	116	116	116
(A6) Tender-Mindedness	Pearson Correlation	-.402**	-.281**	-.251**
	Sig. (2-tailed)	.000	.002	.007
	N	116	116	116
(C1) Competence	Pearson Correlation	-.451**	-.554**	-.517**
	Sig. (2-tailed)	.000	.000	.000
	N	116	116	116
(C2) Order	Pearson Correlation	-.124	-.197*	-.171
	Sig. (2-tailed)	.185	.034	.066
	N	116	116	116
(C3) Dutifulness	Pearson Correlation	-.503**	-.568**	-.480**
	Sig. (2-tailed)	.000	.000	.000
	N	116	116	116
(C4) Achievement Striving	Pearson Correlation	-.155	-.256**	-.291**
	Sig. (2-tailed)	.097	.006	.002
	N	116	116	116
(C5) Self-Discipline	Pearson Correlation	-.528**	-.581**	-.536**
	Sig. (2-tailed)	.000	.000	.000
	N	116	116	116
(C6) Deliberation	Pearson Correlation	-.192*	-.298**	-.177
	Sig. (2-tailed)	.039	.001	.058
	N	116	116	116
MCAT Verbal	Pearson Correlation	.107	.134	.130
	Sig. (2-tailed)	.254	.152	.163
	N	116	116	116

Correlations

		(N5) Impulsiveness	(N6) Vulnerability	(E1) Warmth
(A4) Compliance	Pearson Correlation	-.322**	-.255**	.397**
	Sig. (2-tailed)	.000	.006	.000
	N	116	116	116
(A5) Modesty	Pearson Correlation	.002	.060	.164
	Sig. (2-tailed)	.983	.520	.078
	N	116	116	116
(A6) Tender-Mindedness	Pearson Correlation	-.157	-.237*	.479**
	Sig. (2-tailed)	.092	.010	.000
	N	116	116	116
(C1) Competence	Pearson Correlation	-.380**	-.640**	.369**
	Sig. (2-tailed)	.000	.000	.000
	N	116	116	116
(C2) Order	Pearson Correlation	-.277**	-.098	.199*
	Sig. (2-tailed)	.003	.297	.032
	N	116	116	116
(C3) Dutifulness	Pearson Correlation	-.525**	-.550**	.364**
	Sig. (2-tailed)	.000	.000	.000
	N	116	116	116
(C4) Achievement Striving	Pearson Correlation	-.289**	-.286**	.242**
	Sig. (2-tailed)	.002	.002	.009
	N	116	116	116
(C5) Self-Discipline	Pearson Correlation	-.578**	-.565**	.322**
	Sig. (2-tailed)	.000	.000	.000
	N	116	116	116
(C6) Deliberation	Pearson Correlation	-.398**	-.322**	.197*
	Sig. (2-tailed)	.000	.000	.034
	N	116	116	116
MCAT Verbal	Pearson Correlation	.055	.015	-.161
	Sig. (2-tailed)	.560	.873	.084
	N	116	116	116

Correlations

		(E2) Gregariousness	(E3) Assertiveness	(E4) Activity
(A4) Compliance	Pearson Correlation	.061	.021	.048
	Sig. (2-tailed)	.515	.823	.608
	N	116	116	116
(A5) Modesty	Pearson Correlation	-.071	-.136	-.010
	Sig. (2-tailed)	.449	.146	.918
	N	116	116	116
(A6) Tender-Mindedness	Pearson Correlation	.291**	.255**	.331**
	Sig. (2-tailed)	.002	.006	.000
	N	116	116	116
(C1) Competence	Pearson Correlation	.229*	.323**	.391**
	Sig. (2-tailed)	.013	.000	.000
	N	116	116	116
(C2) Order	Pearson Correlation	.086	.195*	.187*
	Sig. (2-tailed)	.359	.036	.044
	N	116	116	116
(C3) Dutifulness	Pearson Correlation	.130	.277**	.287**
	Sig. (2-tailed)	.165	.003	.002
	N	116	116	116
(C4) Achievement Striving	Pearson Correlation	.132	.365**	.460**
	Sig. (2-tailed)	.158	.000	.000
	N	116	116	116
(C5) Self-Discipline	Pearson Correlation	.223*	.366**	.400**
	Sig. (2-tailed)	.016	.000	.000
	N	116	116	116
(C6) Deliberation	Pearson Correlation	.010	.111	.176
	Sig. (2-tailed)	.915	.235	.058
	N	116	116	116
MCAT Verbal	Pearson Correlation	-.234*	-.219*	-.062
	Sig. (2-tailed)	.011	.018	.508
	N	116	116	116

Correlations

		(E5) Excitement-Seeking	(E6) Positive Emotions	(O1) Fantasy
(A4) Compliance	Pearson Correlation	-.158	.242**	-.257**
	Sig. (2-tailed)	.090	.009	.005
	N	116	116	116
(A5) Modesty	Pearson Correlation	-.047	.034	-.141
	Sig. (2-tailed)	.615	.716	.132
	N	116	116	116
(A6) Tender-Mindedness	Pearson Correlation	.006	.490**	-.116
	Sig. (2-tailed)	.945	.000	.214
	N	116	116	116
(C1) Competence	Pearson Correlation	.044	.267**	-.112
	Sig. (2-tailed)	.639	.004	.232
	N	116	116	116
(C2) Order	Pearson Correlation	-.086	.067	-.294**
	Sig. (2-tailed)	.359	.475	.001
	N	116	116	116
(C3) Dutifulness	Pearson Correlation	-.091	.204*	-.358**
	Sig. (2-tailed)	.331	.028	.000
	N	116	116	116
(C4) Achievement Striving	Pearson Correlation	-.010	.076	-.222*
	Sig. (2-tailed)	.918	.418	.017
	N	116	116	116
(C5) Self-Discipline	Pearson Correlation	-.028	.230*	-.253**
	Sig. (2-tailed)	.769	.013	.006
	N	116	116	116
(C6) Deliberation	Pearson Correlation	-.122	-.119	-.279**
	Sig. (2-tailed)	.193	.202	.002
	N	116	116	116
MCAT Verbal	Pearson Correlation	-.090	-.183*	.279**
	Sig. (2-tailed)	.334	.049	.002
	N	116	116	116

Correlations

		(O2) Aesthetics	(O3) Feelings	(O4) Actions
(A4) Compliance	Pearson Correlation	.094	-.112	.038
	Sig. (2-tailed)	.315	.230	.686
	N	116	116	116
(A5) Modesty	Pearson Correlation	-.156	.046	.010
	Sig. (2-tailed)	.094	.626	.912
	N	116	116	116
(A6) Tender-Mindedness	Pearson Correlation	.148	.202*	.252**
	Sig. (2-tailed)	.113	.029	.006
	N	116	116	116
(C1) Competence	Pearson Correlation	.058	-.018	.060
	Sig. (2-tailed)	.538	.850	.523
	N	116	116	116
(C2) Order	Pearson Correlation	-.039	.013	-.229*
	Sig. (2-tailed)	.677	.890	.014
	N	116	116	116
(C3) Dutifulness	Pearson Correlation	-.071	-.169	-.008
	Sig. (2-tailed)	.447	.069	.930
	N	116	116	116
(C4) Achievement Striving	Pearson Correlation	.081	.048	-.047
	Sig. (2-tailed)	.388	.611	.618
	N	116	116	116
(C5) Self-Discipline	Pearson Correlation	.078	-.137	.080
	Sig. (2-tailed)	.403	.142	.393
	N	116	116	116
(C6) Deliberation	Pearson Correlation	-.126	-.218*	-.133
	Sig. (2-tailed)	.177	.019	.154
	N	116	116	116
MCAT Verbal	Pearson Correlation	.026	.093	-.024
	Sig. (2-tailed)	.785	.322	.798
	N	116	116	116

Correlations

		(O5) Ideas	(O6) Values	(A1) Trust	(A2) Straightforward ness
(A4) Compliance	Pearson Correlation	.100	.069	.528**	.597**
	Sig. (2-tailed)	.283	.464	.000	.000
	N	116	116	116	116
(A5) Modesty	Pearson Correlation	-.029	-.069	.098	.327**
	Sig. (2-tailed)	.758	.461	.293	.000
	N	116	116	116	116
(A6) Tender-Mindedness	Pearson Correlation	.161	.105	.460**	.483**
	Sig. (2-tailed)	.084	.263	.000	.000
	N	116	116	116	116
(C1) Competence	Pearson Correlation	.227*	-.112	.315**	.352**
	Sig. (2-tailed)	.014	.230	.001	.000
	N	116	116	116	116
(C2) Order	Pearson Correlation	-.042	-.073	.148	.358**
	Sig. (2-tailed)	.653	.439	.112	.000
	N	116	116	116	116
(C3) Dutifulness	Pearson Correlation	.185*	-.079	.324**	.518**
	Sig. (2-tailed)	.047	.398	.000	.000
	N	116	116	116	116
(C4) Achievement Striving	Pearson Correlation	.224*	-.038	.105	.289**
	Sig. (2-tailed)	.016	.683	.261	.002
	N	116	116	116	116
(C5) Self-Discipline	Pearson Correlation	.199*	-.005	.363**	.406**
	Sig. (2-tailed)	.032	.954	.000	.000
	N	116	116	116	116
(C6) Deliberation	Pearson Correlation	.073	-.174	.166	.355**
	Sig. (2-tailed)	.436	.062	.075	.000
	N	116	116	116	116
MCAT Verbal	Pearson Correlation	.034	.070	.031	-.189*
	Sig. (2-tailed)	.720	.455	.739	.042
	N	116	116	116	116

Correlations

		(A3) Altruism	(A4) Compliance	(A5) Modesty
(A4) Compliance	Pearson Correlation	.518**	1	.312**
	Sig. (2-tailed)	.000		.001
	N	116	116	116
(A5) Modesty	Pearson Correlation	.160	.312**	1
	Sig. (2-tailed)	.086	.001	
	N	116	116	116
(A6) Tender-Mindedness	Pearson Correlation	.611**	.446**	.184*
	Sig. (2-tailed)	.000	.000	.048
	N	116	116	116
(C1) Competence	Pearson Correlation	.521**	.259**	-.090
	Sig. (2-tailed)	.000	.005	.339
	N	116	116	116
(C2) Order	Pearson Correlation	.217*	.219*	-.094
	Sig. (2-tailed)	.019	.018	.316
	N	116	116	116
(C3) Dutifulness	Pearson Correlation	.519**	.385**	.115
	Sig. (2-tailed)	.000	.000	.220
	N	116	116	116
(C4) Achievement Striving	Pearson Correlation	.253**	.113	-.061
	Sig. (2-tailed)	.006	.228	.516
	N	116	116	116
(C5) Self-Discipline	Pearson Correlation	.388**	.372**	-.067
	Sig. (2-tailed)	.000	.000	.472
	N	116	116	116
(C6) Deliberation	Pearson Correlation	.340**	.224*	.141
	Sig. (2-tailed)	.000	.016	.132
	N	116	116	116
MCAT Verbal	Pearson Correlation	-.269**	-.014	.008
	Sig. (2-tailed)	.003	.883	.930
	N	116	116	116

Correlations

		(A6) Tender-Mindedness	(C1) Competence	(C2) Order
(A4) Compliance	Pearson Correlation	.446**	.259**	.219*
	Sig. (2-tailed)	.000	.005	.018
	N	116	116	116
(A5) Modesty	Pearson Correlation	.184*	-.090	-.094
	Sig. (2-tailed)	.048	.339	.316
	N	116	116	116
(A6) Tender-Mindedness	Pearson Correlation	1	.251**	.195*
	Sig. (2-tailed)		.007	.036
	N	116	116	116
(C1) Competence	Pearson Correlation	.251**	1	.417**
	Sig. (2-tailed)	.007		.000
	N	116	116	116
(C2) Order	Pearson Correlation	.195*	.417**	1
	Sig. (2-tailed)	.036	.000	
	N	116	116	116
(C3) Dutifulness	Pearson Correlation	.324**	.701**	.420**
	Sig. (2-tailed)	.000	.000	.000
	N	116	116	116
(C4) Achievement Striving	Pearson Correlation	.242**	.489**	.493**
	Sig. (2-tailed)	.009	.000	.000
	N	116	116	116
(C5) Self-Discipline	Pearson Correlation	.257**	.700**	.530**
	Sig. (2-tailed)	.005	.000	.000
	N	116	116	116
(C6) Deliberation	Pearson Correlation	.113	.507**	.323**
	Sig. (2-tailed)	.226	.000	.000
	N	116	116	116
MCAT Verbal	Pearson Correlation	-.254**	-.085	-.092
	Sig. (2-tailed)	.006	.362	.326
	N	116	116	116

Correlations

		(C3) Dutifulness	(C4) Achievement Striving	(C5) Self-Discipline
(A4) Compliance	Pearson Correlation	.385**	.113	.372**
	Sig. (2-tailed)	.000	.228	.000
	N	116	116	116
(A5) Modesty	Pearson Correlation	.115	-.061	-.067
	Sig. (2-tailed)	.220	.516	.472
	N	116	116	116
(A6) Tender-Mindedness	Pearson Correlation	.324**	.242**	.257**
	Sig. (2-tailed)	.000	.009	.005
	N	116	116	116
(C1) Competence	Pearson Correlation	.701**	.489**	.700**
	Sig. (2-tailed)	.000	.000	.000
	N	116	116	116
(C2) Order	Pearson Correlation	.420**	.493**	.530**
	Sig. (2-tailed)	.000	.000	.000
	N	116	116	116
(C3) Dutifulness	Pearson Correlation	1	.548**	.673**
	Sig. (2-tailed)		.000	.000
	N	116	116	116
(C4) Achievement Striving	Pearson Correlation	.548**	1	.662**
	Sig. (2-tailed)	.000		.000
	N	116	116	116
(C5) Self-Discipline	Pearson Correlation	.673**	.662**	1
	Sig. (2-tailed)	.000	.000	
	N	116	116	116
(C6) Deliberation	Pearson Correlation	.558**	.360**	.429**
	Sig. (2-tailed)	.000	.000	.000
	N	116	116	116
MCAT Verbal	Pearson Correlation	-.317**	-.226*	-.176
	Sig. (2-tailed)	.001	.015	.059
	N	116	116	116

Correlations

		(C6) Deliberation	MCAT Verbal	MCAT Biological Sciences
(A4) Compliance	Pearson Correlation	.224*	-.014	.082
	Sig. (2-tailed)	.016	.883	.379
	N	116	116	116
(A5) Modesty	Pearson Correlation	.141	.008	-.065
	Sig. (2-tailed)	.132	.930	.489
	N	116	116	116
(A6) Tender-Mindedness	Pearson Correlation	.113	-.254**	-.063
	Sig. (2-tailed)	.226	.006	.500
	N	116	116	116
(C1) Competence	Pearson Correlation	.507**	-.085	.032
	Sig. (2-tailed)	.000	.362	.735
	N	116	116	116
(C2) Order	Pearson Correlation	.323**	-.092	-.153
	Sig. (2-tailed)	.000	.326	.101
	N	116	116	116
(C3) Dutifulness	Pearson Correlation	.558**	-.317**	.011
	Sig. (2-tailed)	.000	.001	.906
	N	116	116	116
(C4) Achievement Striving	Pearson Correlation	.360**	-.226*	-.112
	Sig. (2-tailed)	.000	.015	.233
	N	116	116	116
(C5) Self-Discipline	Pearson Correlation	.429**	-.176	.047
	Sig. (2-tailed)	.000	.059	.616
	N	116	116	116
(C6) Deliberation	Pearson Correlation	1	-.189*	.000
	Sig. (2-tailed)		.042	.998
	N	116	116	116
MCAT Verbal	Pearson Correlation	-.189*	1	.022
	Sig. (2-tailed)	.042		.813
	N	116	116	116

Correlations

		MCAT Physical Sciences	Yr 1 Phase A MDE	Yr 1 Phase B MDE
(A4) Compliance	Pearson Correlation	-.065	.125	.119
	Sig. (2-tailed)	.487	.181	.204
	N	116	115	115
(A5) Modesty	Pearson Correlation	-.013	.029	.044
	Sig. (2-tailed)	.890	.756	.638
	N	116	115	115
(A6) Tender-Mindedness	Pearson Correlation	-.100	-.083	-.092
	Sig. (2-tailed)	.285	.379	.328
	N	116	115	115
(C1) Competence	Pearson Correlation	-.173	.066	.077
	Sig. (2-tailed)	.063	.485	.415
	N	116	115	115
(C2) Order	Pearson Correlation	-.303**	-.096	.019
	Sig. (2-tailed)	.001	.309	.841
	N	116	115	115
(C3) Dutifulness	Pearson Correlation	-.077	.075	.108
	Sig. (2-tailed)	.410	.425	.249
	N	116	115	115
(C4) Achievement Striving	Pearson Correlation	-.161	-.054	-.031
	Sig. (2-tailed)	.084	.567	.741
	N	116	115	115
(C5) Self-Discipline	Pearson Correlation	-.173	.053	.124
	Sig. (2-tailed)	.063	.577	.188
	N	116	115	115
(C6) Deliberation	Pearson Correlation	-.033	.116	.143
	Sig. (2-tailed)	.723	.216	.128
	N	116	115	115
MCAT Verbal	Pearson Correlation	.044	.067	.084
	Sig. (2-tailed)	.641	.476	.370
	N	116	115	115

Correlations

		Yr 1 Host Defense MDE	Yr 2 GI MDE	Yr 2 Renal MDE
(A4) Compliance	Pearson Correlation	.196*	.293	.196
	Sig. (2-tailed)	.038	.051	.197
	N	113	45	45
(A5) Modesty	Pearson Correlation	.120	.000	-.005
	Sig. (2-tailed)	.206	.998	.972
	N	113	45	45
(A6) Tender-Mindedness	Pearson Correlation	-.010	.248	.030
	Sig. (2-tailed)	.916	.101	.847
	N	113	45	45
(C1) Competence	Pearson Correlation	.160	.133	.182
	Sig. (2-tailed)	.091	.384	.231
	N	113	45	45
(C2) Order	Pearson Correlation	.131	.276	.055
	Sig. (2-tailed)	.165	.067	.721
	N	113	45	45
(C3) Dutifulness	Pearson Correlation	.119	.389**	.192
	Sig. (2-tailed)	.210	.008	.207
	N	113	45	45
(C4) Achievement Striving	Pearson Correlation	.031	.316*	.073
	Sig. (2-tailed)	.745	.034	.636
	N	113	45	45
(C5) Self-Discipline	Pearson Correlation	.173	.458**	.273
	Sig. (2-tailed)	.067	.002	.070
	N	113	45	45
(C6) Deliberation	Pearson Correlation	.279**	.092	.055
	Sig. (2-tailed)	.003	.546	.721
	N	113	45	45
MCAT Verbal	Pearson Correlation	-.098	-.491**	-.223
	Sig. (2-tailed)	.304	.001	.140
	N	113	45	45

Correlations

		Yr 2 Infectious Diseases MDE	Yr 1 Hematology MDE	Yr 1 Neurology MDE
(A4) Compliance	Pearson Correlation	.123	.194*	.162
	Sig. (2-tailed)	.422	.044	.289
	N	45	109	45
(A5) Modesty	Pearson Correlation	-.121	-.097	.014
	Sig. (2-tailed)	.428	.315	.926
	N	45	109	45
(A6) Tender-Mindedness	Pearson Correlation	.106	-.011	.159
	Sig. (2-tailed)	.489	.908	.296
	N	45	109	45
(C1) Competence	Pearson Correlation	.239	.205*	.246
	Sig. (2-tailed)	.114	.033	.103
	N	45	109	45
(C2) Order	Pearson Correlation	.284	.105	.208
	Sig. (2-tailed)	.059	.278	.170
	N	45	109	45
(C3) Dutifulness	Pearson Correlation	.309*	.059	.398**
	Sig. (2-tailed)	.039	.541	.007
	N	45	109	45
(C4) Achievement Striving	Pearson Correlation	.111	.119	.336*
	Sig. (2-tailed)	.468	.219	.024
	N	45	109	45
(C5) Self-Discipline	Pearson Correlation	.299*	.189*	.463**
	Sig. (2-tailed)	.046	.049	.001
	N	45	109	45
(C6) Deliberation	Pearson Correlation	.226	.095	.222
	Sig. (2-tailed)	.136	.325	.143
	N	45	109	45
MCAT Verbal	Pearson Correlation	-.401**	.054	-.369*
	Sig. (2-tailed)	.006	.577	.013
	N	45	109	45

Correlations

		Yr 1 Brain & Behavior MDE	Yr 1 Musculoskeletal MDE	Yr 1 Community Epidimiology Study Grade
(A4) Compliance	Pearson Correlation	.216	.205	-.018
	Sig. (2-tailed)	.059	.177	.851
	N	77	45	115
(A5) Modesty	Pearson Correlation	-.009	.012	-.040
	Sig. (2-tailed)	.937	.940	.668
	N	77	45	115
(A6) Tender-Mindedness	Pearson Correlation	-.065	.081	-.212*
	Sig. (2-tailed)	.574	.595	.023
	N	77	45	115
(C1) Competence	Pearson Correlation	.165	.250	.058
	Sig. (2-tailed)	.150	.097	.540
	N	77	45	115
(C2) Order	Pearson Correlation	.146	.141	-.023
	Sig. (2-tailed)	.206	.355	.810
	N	77	45	115
(C3) Dutifulness	Pearson Correlation	.109	.316*	-.094
	Sig. (2-tailed)	.347	.034	.319
	N	77	45	115
(C4) Achievement Striving	Pearson Correlation	.023	.154	-.159
	Sig. (2-tailed)	.843	.312	.091
	N	77	45	115
(C5) Self-Discipline	Pearson Correlation	.140	.388**	-.042
	Sig. (2-tailed)	.224	.008	.653
	N	77	45	115
(C6) Deliberation	Pearson Correlation	.166	.056	-.026
	Sig. (2-tailed)	.150	.715	.780
	N	77	45	115
MCAT Verbal	Pearson Correlation	-.002	-.269	.323**
	Sig. (2-tailed)	.984	.074	.000
	N	77	45	115

Correlations

		Yr 2 Cardiology MDE	Yr 2 Biostatistics	Yr 2 Pulmonology MDE
(A4) Compliance	Pearson Correlation	.205	-.022	.167
	Sig. (2-tailed)	.176	.888	.271
	N	45	45	45
(A5) Modesty	Pearson Correlation	-.010	.135	-.074
	Sig. (2-tailed)	.947	.378	.630
	N	45	45	45
(A6) Tender-Mindedness	Pearson Correlation	.101	-.026	.069
	Sig. (2-tailed)	.507	.864	.652
	N	45	45	45
(C1) Competence	Pearson Correlation	.249	-.027	.224
	Sig. (2-tailed)	.099	.859	.140
	N	45	45	45
(C2) Order	Pearson Correlation	.117	-.102	.025
	Sig. (2-tailed)	.443	.505	.870
	N	45	45	45
(C3) Dutifulness	Pearson Correlation	.343*	.039	.268
	Sig. (2-tailed)	.021	.797	.075
	N	45	45	45
(C4) Achievement Striving	Pearson Correlation	.301*	.029	.042
	Sig. (2-tailed)	.044	.850	.786
	N	45	45	45
(C5) Self-Discipline	Pearson Correlation	.466**	.128	.328*
	Sig. (2-tailed)	.001	.404	.028
	N	45	45	45
(C6) Deliberation	Pearson Correlation	.065	-.079	.036
	Sig. (2-tailed)	.671	.607	.814
	N	45	45	45
MCAT Verbal	Pearson Correlation	-.196	.060	-.099
	Sig. (2-tailed)	.196	.696	.519
	N	45	45	45

Correlations

		Yr 2 Endocrinology MDE	Yr 2 Research Design	Repeat Phase A MDE
(A4) Compliance	Pearson Correlation	.173	-.024	.138
	Sig. (2-tailed)	.250	.874	.724
	N	46	45	9
(A5) Modesty	Pearson Correlation	-.100	.228	-.184
	Sig. (2-tailed)	.511	.132	.636
	N	46	45	9
(A6) Tender-Mindedness	Pearson Correlation	.026	.056	.060
	Sig. (2-tailed)	.866	.717	.879
	N	46	45	9
(C1) Competence	Pearson Correlation	.075	.051	-.131
	Sig. (2-tailed)	.619	.740	.737
	N	46	45	9
(C2) Order	Pearson Correlation	.024	-.056	.088
	Sig. (2-tailed)	.874	.717	.823
	N	46	45	9
(C3) Dutifulness	Pearson Correlation	.103	.022	-.206
	Sig. (2-tailed)	.497	.888	.595
	N	46	45	9
(C4) Achievement Striving	Pearson Correlation	-.033	.059	-.378
	Sig. (2-tailed)	.829	.699	.316
	N	46	45	9
(C5) Self-Discipline	Pearson Correlation	.221	.081	-.404
	Sig. (2-tailed)	.140	.597	.280
	N	46	45	9
(C6) Deliberation	Pearson Correlation	.090	-.042	-.252
	Sig. (2-tailed)	.554	.786	.513
	N	46	45	9
MCAT Verbal	Pearson Correlation	-.200	.279	-.132
	Sig. (2-tailed)	.183	.064	.735
	N	46	45	9

Correlations

		Repeat Phase B MDE	Repeat Phase A SAP	Repeat Host Defense MDE
(A4) Compliance	Pearson Correlation	.138	-.042	.133
	Sig. (2-tailed)	.723	.914	.732
	N	9	9	9
(A5) Modesty	Pearson Correlation	-.538	-.253	-.217
	Sig. (2-tailed)	.135	.511	.575
	N	9	9	9
(A6) Tender-Mindedness	Pearson Correlation	-.025	.474	.061
	Sig. (2-tailed)	.950	.198	.876
	N	9	9	9
(C1) Competence	Pearson Correlation	.372	.256	.060
	Sig. (2-tailed)	.324	.506	.879
	N	9	9	9
(C2) Order	Pearson Correlation	.456	.446	.313
	Sig. (2-tailed)	.218	.229	.412
	N	9	9	9
(C3) Dutifulness	Pearson Correlation	-.128	.201	-.031
	Sig. (2-tailed)	.743	.605	.936
	N	9	9	9
(C4) Achievement Striving	Pearson Correlation	.134	.357	-.178
	Sig. (2-tailed)	.731	.345	.648
	N	9	9	9
(C5) Self-Discipline	Pearson Correlation	.147	.322	-.289
	Sig. (2-tailed)	.705	.397	.450
	N	9	9	9
(C6) Deliberation	Pearson Correlation	-.573	-.237	-.009
	Sig. (2-tailed)	.106	.540	.981
	N	9	9	9
MCAT Verbal	Pearson Correlation	.261	.208	-.378
	Sig. (2-tailed)	.497	.591	.315
	N	9	9	9

Correlations

		Repeat Hematology MDE	Repeat Neurology MDE	Repeat Brain & Behavior MDE
(A4) Compliance	Pearson Correlation	.374	-.139	.239
	Sig. (2-tailed)	.321	.722	.536
	N	9	9	9
(A5) Modesty	Pearson Correlation	.036	-.056	.019
	Sig. (2-tailed)	.926	.887	.960
	N	9	9	9
(A6) Tender-Mindedness	Pearson Correlation	-.151	-.106	.611
	Sig. (2-tailed)	.699	.786	.080
	N	9	9	9
(C1) Competence	Pearson Correlation	-.310	-.113	.761*
	Sig. (2-tailed)	.417	.771	.017
	N	9	9	9
(C2) Order	Pearson Correlation	-.482	-.231	.450
	Sig. (2-tailed)	.189	.549	.224
	N	9	9	9
(C3) Dutifulness	Pearson Correlation	-.171	-.192	.776*
	Sig. (2-tailed)	.660	.621	.014
	N	9	9	9
(C4) Achievement Striving	Pearson Correlation	-.353	.208	.545
	Sig. (2-tailed)	.351	.591	.129
	N	9	9	9
(C5) Self-Discipline	Pearson Correlation	-.289	.331	.510
	Sig. (2-tailed)	.450	.385	.161
	N	9	9	9
(C6) Deliberation	Pearson Correlation	-.155	-.332	.614
	Sig. (2-tailed)	.691	.382	.078
	N	9	9	9
MCAT Verbal	Pearson Correlation	-.009	.385	-.086
	Sig. (2-tailed)	.982	.306	.827
	N	9	9	9

Correlations

		Repeat Musculoskeletal MDE
(A4) Compliance	Pearson Correlation	.530
	Sig. (2-tailed)	.142
	N	9
(A5) Modesty	Pearson Correlation	.147
	Sig. (2-tailed)	.705
	N	9
(A6) Tender-Mindedness	Pearson Correlation	.529
	Sig. (2-tailed)	.143
	N	9
(C1) Competence	Pearson Correlation	.085
	Sig. (2-tailed)	.828
	N	9
(C2) Order	Pearson Correlation	-.112
	Sig. (2-tailed)	.775
	N	9
(C3) Dutifulness	Pearson Correlation	.509
	Sig. (2-tailed)	.161
	N	9
(C4) Achievement Striving	Pearson Correlation	.272
	Sig. (2-tailed)	.479
	N	9
(C5) Self-Discipline	Pearson Correlation	.194
	Sig. (2-tailed)	.617
	N	9
(C6) Deliberation	Pearson Correlation	.523
	Sig. (2-tailed)	.149
	N	9
MCAT Verbal	Pearson Correlation	-.834**
	Sig. (2-tailed)	.005
	N	9

Correlations

		(N) Neuroticism	(E) Extraversion	(O) Openness
MCAT Biological Sciences	Pearson Correlation	-.008	-.236*	.235*
	Sig. (2-tailed)	.929	.011	.011
	N	116	116	116
MCAT Physical Sciences	Pearson Correlation	.005	-.069	.182
	Sig. (2-tailed)	.958	.464	.051
	N	116	116	116
Yr 1 Phase A MDE	Pearson Correlation	-.077	-.193*	-.038
	Sig. (2-tailed)	.411	.038	.685
	N	115	115	115
Yr 1 Phase B MDE	Pearson Correlation	-.144	-.154	-.054
	Sig. (2-tailed)	.124	.100	.570
	N	115	115	115
Yr 1 Host Defense MDE	Pearson Correlation	-.006	-.047	-.125
	Sig. (2-tailed)	.947	.620	.186
	N	113	113	113
Yr 2 GI MDE	Pearson Correlation	.005	.189	-.029
	Sig. (2-tailed)	.973	.214	.852
	N	45	45	45
Yr 2 Renal MDE	Pearson Correlation	-.077	.097	-.152
	Sig. (2-tailed)	.615	.526	.319
	N	45	45	45
Yr 2 Infectious Diseases MDE	Pearson Correlation	.013	.057	-.246
	Sig. (2-tailed)	.935	.711	.103
	N	45	45	45
Yr 1 Hematology MDE	Pearson Correlation	-.001	-.007	-.029
	Sig. (2-tailed)	.991	.944	.767
	N	109	109	109
Yr 1 Neurology MDE	Pearson Correlation	-.165	.077	-.122
	Sig. (2-tailed)	.279	.616	.423
	N	45	45	45

Correlations

		(A) Agreeableness	(C) Conscientiousness	(N1) Anxiety
MCAT Biological Sciences	Pearson Correlation	.024	-.058	-.084
	Sig. (2-tailed)	.796	.533	.368
	N	116	116	116
MCAT Physical Sciences	Pearson Correlation	-.037	-.207*	-.053
	Sig. (2-tailed)	.695	.026	.575
	N	116	116	116
Yr 1 Phase A MDE	Pearson Correlation	.026	-.019	-.088
	Sig. (2-tailed)	.784	.837	.349
	N	115	115	115
Yr 1 Phase B MDE	Pearson Correlation	.042	.030	-.143
	Sig. (2-tailed)	.655	.747	.128
	N	115	115	115
Yr 1 Host Defense MDE	Pearson Correlation	.095	.153	.000
	Sig. (2-tailed)	.317	.106	1.000
	N	113	113	113
Yr 2 GI MDE	Pearson Correlation	.206	.347*	-.033
	Sig. (2-tailed)	.175	.020	.829
	N	45	45	45
Yr 2 Renal MDE	Pearson Correlation	.049	.143	-.096
	Sig. (2-tailed)	.747	.348	.531
	N	45	45	45
Yr 2 Infectious Diseases MDE	Pearson Correlation	.090	.313*	-.007
	Sig. (2-tailed)	.555	.036	.964
	N	45	45	45
Yr 1 Hematology MDE	Pearson Correlation	.031	.169	-.012
	Sig. (2-tailed)	.753	.079	.904
	N	109	109	109
Yr 1 Neurology MDE	Pearson Correlation	.091	.382**	-.213
	Sig. (2-tailed)	.551	.010	.159
	N	45	45	45

Correlations

		(N2) Angry Hostility	(N3) Depression	(N4) Self-Consciousness
MCAT Biological Sciences	Pearson Correlation	.004	.061	.107
	Sig. (2-tailed)	.963	.517	.252
	N	116	116	116
MCAT Physical Sciences	Pearson Correlation	.104	.077	.099
	Sig. (2-tailed)	.268	.413	.290
	N	116	116	116
Yr 1 Phase A MDE	Pearson Correlation	-.076	-.049	.098
	Sig. (2-tailed)	.421	.603	.297
	N	115	115	115
Yr 1 Phase B MDE	Pearson Correlation	-.102	-.126	-.006
	Sig. (2-tailed)	.280	.181	.949
	N	115	115	115
Yr 1 Host Defense MDE	Pearson Correlation	-.102	-.070	.084
	Sig. (2-tailed)	.282	.458	.378
	N	113	113	113
Yr 2 GI MDE	Pearson Correlation	-.152	-.205	-.008
	Sig. (2-tailed)	.319	.178	.959
	N	45	45	45
Yr 2 Renal MDE	Pearson Correlation	-.106	-.097	.017
	Sig. (2-tailed)	.486	.526	.910
	N	45	45	45
Yr 2 Infectious Diseases MDE	Pearson Correlation	-.075	-.159	.077
	Sig. (2-tailed)	.624	.297	.616
	N	45	45	45
Yr 1 Hematology MDE	Pearson Correlation	-.093	.010	.103
	Sig. (2-tailed)	.337	.918	.287
	N	109	109	109
Yr 1 Neurology MDE	Pearson Correlation	-.131	-.293	-.140
	Sig. (2-tailed)	.392	.050	.361
	N	45	45	45

Correlations

		(N5) Impulsiveness	(N6) Vulnerability	(E1) Warmth
MCAT Biological Sciences	Pearson Correlation	.004	.015	-.159
	Sig. (2-tailed)	.970	.872	.088
	N	116	116	116
MCAT Physical Sciences	Pearson Correlation	.082	.044	.038
	Sig. (2-tailed)	.384	.642	.685
	N	116	116	116
Yr 1 Phase A MDE	Pearson Correlation	.066	-.057	-.155
	Sig. (2-tailed)	.481	.547	.097
	N	115	115	115
Yr 1 Phase B MDE	Pearson Correlation	-.045	-.105	-.106
	Sig. (2-tailed)	.631	.265	.259
	N	115	115	115
Yr 1 Host Defense MDE	Pearson Correlation	.068	-.110	-.053
	Sig. (2-tailed)	.476	.244	.576
	N	113	113	113
Yr 2 GI MDE	Pearson Correlation	-.151	-.118	.155
	Sig. (2-tailed)	.323	.441	.309
	N	45	45	45
Yr 2 Renal MDE	Pearson Correlation	.036	-.120	.086
	Sig. (2-tailed)	.815	.431	.576
	N	45	45	45
Yr 2 Infectious Diseases MDE	Pearson Correlation	-.041	-.035	.019
	Sig. (2-tailed)	.788	.818	.904
	N	45	45	45
Yr 1 Hematology MDE	Pearson Correlation	-.011	-.163	-.001
	Sig. (2-tailed)	.908	.091	.989
	N	109	109	109
Yr 1 Neurology MDE	Pearson Correlation	-.160	-.235	.116
	Sig. (2-tailed)	.293	.120	.448
	N	45	45	45

Correlations

		(E2) Gregariousness	(E3) Assertiveness	(E4) Activity
MCAT Biological Sciences	Pearson Correlation	-.177	-.322**	-.216*
	Sig. (2-tailed)	.058	.000	.020
	N	116	116	116
MCAT Physical Sciences	Pearson Correlation	-.100	-.177	-.107
	Sig. (2-tailed)	.286	.058	.254
	N	116	116	116
Yr 1 Phase A MDE	Pearson Correlation	-.149	-.268**	-.230*
	Sig. (2-tailed)	.112	.004	.013
	N	115	115	115
Yr 1 Phase B MDE	Pearson Correlation	-.061	-.224*	-.169
	Sig. (2-tailed)	.518	.016	.072
	N	115	115	115
Yr 1 Host Defense MDE	Pearson Correlation	.030	-.144	-.099
	Sig. (2-tailed)	.753	.128	.298
	N	113	113	113
Yr 2 GI MDE	Pearson Correlation	.158	-.058	.189
	Sig. (2-tailed)	.300	.707	.213
	N	45	45	45
Yr 2 Renal MDE	Pearson Correlation	-.054	-.027	.082
	Sig. (2-tailed)	.722	.858	.594
	N	45	45	45
Yr 2 Infectious Diseases MDE	Pearson Correlation	.060	-.112	.005
	Sig. (2-tailed)	.695	.464	.976
	N	45	45	45
Yr 1 Hematology MDE	Pearson Correlation	-.042	.003	.085
	Sig. (2-tailed)	.663	.977	.379
	N	109	109	109
Yr 1 Neurology MDE	Pearson Correlation	-.038	.242	.148
	Sig. (2-tailed)	.805	.110	.331
	N	45	45	45

Correlations

		(E5) Excitement-Seeking	(E6) Positive Emotions	(O1) Fantasy
MCAT Biological Sciences	Pearson Correlation	-.027	-.003	.023
	Sig. (2-tailed)	.772	.975	.810
	N	116	116	116
MCAT Physical Sciences	Pearson Correlation	.041	.005	.140
	Sig. (2-tailed)	.659	.961	.134
	N	116	116	116
Yr 1 Phase A MDE	Pearson Correlation	.041	-.087	-.089
	Sig. (2-tailed)	.667	.356	.345
	N	115	115	115
Yr 1 Phase B MDE	Pearson Correlation	.001	-.006	-.078
	Sig. (2-tailed)	.990	.949	.407
	N	115	115	115
Yr 1 Host Defense MDE	Pearson Correlation	.101	-.013	-.104
	Sig. (2-tailed)	.288	.891	.274
	N	113	113	113
Yr 2 GI MDE	Pearson Correlation	.052	.426**	-.045
	Sig. (2-tailed)	.735	.004	.767
	N	45	45	45
Yr 2 Renal MDE	Pearson Correlation	.042	.239	-.157
	Sig. (2-tailed)	.783	.114	.302
	N	45	45	45
Yr 2 Infectious Diseases MDE	Pearson Correlation	.045	.139	-.355*
	Sig. (2-tailed)	.770	.363	.017
	N	45	45	45
Yr 1 Hematology MDE	Pearson Correlation	.007	.024	-.034
	Sig. (2-tailed)	.946	.803	.725
	N	109	109	109
Yr 1 Neurology MDE	Pearson Correlation	-.055	.233	-.325*
	Sig. (2-tailed)	.721	.123	.029
	N	45	45	45

Correlations

		(O2) Aesthetics	(O3) Feelings	(O4) Actions
MCAT Biological Sciences	Pearson Correlation	.195*	.036	.146
	Sig. (2-tailed)	.036	.701	.118
	N	116	116	116
MCAT Physical Sciences	Pearson Correlation	.068	.004	.155
	Sig. (2-tailed)	.470	.962	.097
	N	116	116	116
Yr 1 Phase A MDE	Pearson Correlation	-.106	-.201*	.002
	Sig. (2-tailed)	.259	.032	.982
	N	115	115	115
Yr 1 Phase B MDE	Pearson Correlation	-.065	-.182	.003
	Sig. (2-tailed)	.489	.051	.977
	N	115	115	115
Yr 1 Host Defense MDE	Pearson Correlation	-.054	-.153	-.039
	Sig. (2-tailed)	.570	.105	.684
	N	113	113	113
Yr 2 GI MDE	Pearson Correlation	.094	-.035	.037
	Sig. (2-tailed)	.539	.820	.811
	N	45	45	45
Yr 2 Renal MDE	Pearson Correlation	-.146	-.177	.009
	Sig. (2-tailed)	.338	.245	.955
	N	45	45	45
Yr 2 Infectious Diseases MDE	Pearson Correlation	-.046	-.201	-.140
	Sig. (2-tailed)	.763	.186	.360
	N	45	45	45
Yr 1 Hematology MDE	Pearson Correlation	.076	-.161	-.035
	Sig. (2-tailed)	.430	.094	.719
	N	109	109	109
Yr 1 Neurology MDE	Pearson Correlation	-.066	-.143	-.083
	Sig. (2-tailed)	.666	.349	.587
	N	45	45	45

Correlations

		(O5) Ideas	(O6) Values	(A1) Trust	(A2) Straightforward ness
MCAT Biological Sciences	Pearson Correlation	.141	.116	-.012	-.033
	Sig. (2-tailed)	.130	.216	.901	.727
	N	116	116	116	116
MCAT Physical Sciences	Pearson Correlation	.149	.077	-.065	-.126
	Sig. (2-tailed)	.111	.410	.486	.179
	N	116	116	116	116
Yr 1 Phase A MDE	Pearson Correlation	.018	.058	-.070	.030
	Sig. (2-tailed)	.849	.540	.456	.754
	N	115	115	115	115
Yr 1 Phase B MDE	Pearson Correlation	-.026	.011	.039	.048
	Sig. (2-tailed)	.781	.906	.680	.611
	N	115	115	115	115
Yr 1 Host Defense MDE	Pearson Correlation	-.036	-.102	-.038	.057
	Sig. (2-tailed)	.703	.283	.686	.551
	N	113	113	113	113
Yr 2 GI MDE	Pearson Correlation	.099	-.145	.069	.133
	Sig. (2-tailed)	.517	.340	.653	.384
	N	45	45	45	45
Yr 2 Renal MDE	Pearson Correlation	-.070	-.076	-.023	.025
	Sig. (2-tailed)	.650	.620	.879	.868
	N	45	45	45	45
Yr 2 Infectious Diseases MDE	Pearson Correlation	-.109	-.115	.022	.065
	Sig. (2-tailed)	.477	.451	.886	.670
	N	45	45	45	45
Yr 1 Hematology MDE	Pearson Correlation	.006	-.057	-.081	-.003
	Sig. (2-tailed)	.951	.557	.405	.973
	N	109	109	109	109
Yr 1 Neurology MDE	Pearson Correlation	.017	.074	-.021	.118
	Sig. (2-tailed)	.912	.631	.892	.439
	N	45	45	45	45

Correlations

		(A3) Altruism	(A4) Compliance	(A5) Modesty
MCAT Biological Sciences	Pearson Correlation	-.122	.082	-.065
	Sig. (2-tailed)	.192	.379	.489
	N	116	116	116
MCAT Physical Sciences	Pearson Correlation	-.017	-.065	-.013
	Sig. (2-tailed)	.856	.487	.890
	N	116	116	116
Yr 1 Phase A MDE	Pearson Correlation	-.065	.125	.029
	Sig. (2-tailed)	.490	.181	.756
	N	115	115	115
Yr 1 Phase B MDE	Pearson Correlation	-.101	.119	.044
	Sig. (2-tailed)	.284	.204	.638
	N	115	115	115
Yr 1 Host Defense MDE	Pearson Correlation	.036	.196*	.120
	Sig. (2-tailed)	.703	.038	.206
	N	113	113	113
Yr 2 GI MDE	Pearson Correlation	.169	.293	.000
	Sig. (2-tailed)	.268	.051	.998
	N	45	45	45
Yr 2 Renal MDE	Pearson Correlation	-.001	.196	-.005
	Sig. (2-tailed)	.994	.197	.972
	N	45	45	45
Yr 2 Infectious Diseases MDE	Pearson Correlation	.132	.123	-.121
	Sig. (2-tailed)	.386	.422	.428
	N	45	45	45
Yr 1 Hematology MDE	Pearson Correlation	.109	.194*	-.097
	Sig. (2-tailed)	.261	.044	.315
	N	109	109	109
Yr 1 Neurology MDE	Pearson Correlation	.139	.162	.014
	Sig. (2-tailed)	.363	.289	.926
	N	45	45	45

Correlations

		(A6) Tender-Mindedness	(C1) Competence	(C2) Order
MCAT Biological Sciences	Pearson Correlation	-.063	.032	-.153
	Sig. (2-tailed)	.500	.735	.101
	N	116	116	116
MCAT Physical Sciences	Pearson Correlation	-.100	-.173	-.303**
	Sig. (2-tailed)	.285	.063	.001
	N	116	116	116
Yr 1 Phase A MDE	Pearson Correlation	-.083	.066	-.096
	Sig. (2-tailed)	.379	.485	.309
	N	115	115	115
Yr 1 Phase B MDE	Pearson Correlation	-.092	.077	.019
	Sig. (2-tailed)	.328	.415	.841
	N	115	115	115
Yr 1 Host Defense MDE	Pearson Correlation	-.010	.160	.131
	Sig. (2-tailed)	.916	.091	.165
	N	113	113	113
Yr 2 GI MDE	Pearson Correlation	.248	.133	.276
	Sig. (2-tailed)	.101	.384	.067
	N	45	45	45
Yr 2 Renal MDE	Pearson Correlation	.030	.182	.055
	Sig. (2-tailed)	.847	.231	.721
	N	45	45	45
Yr 2 Infectious Diseases MDE	Pearson Correlation	.106	.239	.284
	Sig. (2-tailed)	.489	.114	.059
	N	45	45	45
Yr 1 Hematology MDE	Pearson Correlation	-.011	.205*	.105
	Sig. (2-tailed)	.908	.033	.278
	N	109	109	109
Yr 1 Neurology MDE	Pearson Correlation	.159	.246	.208
	Sig. (2-tailed)	.296	.103	.170
	N	45	45	45

Correlations

		(C3) Dutifulness	(C4) Achievement Striving	(C5) Self-Discipline
MCAT Biological Sciences	Pearson Correlation	.011	-.112	.047
	Sig. (2-tailed)	.906	.233	.616
	N	116	116	116
MCAT Physical Sciences	Pearson Correlation	-.077	-.161	-.173
	Sig. (2-tailed)	.410	.084	.063
	N	116	116	116
Yr 1 Phase A MDE	Pearson Correlation	.075	-.054	.053
	Sig. (2-tailed)	.425	.567	.577
	N	115	115	115
Yr 1 Phase B MDE	Pearson Correlation	.108	-.031	.124
	Sig. (2-tailed)	.249	.741	.188
	N	115	115	115
Yr 1 Host Defense MDE	Pearson Correlation	.119	.031	.173
	Sig. (2-tailed)	.210	.745	.067
	N	113	113	113
Yr 2 GI MDE	Pearson Correlation	.389**	.316*	.458**
	Sig. (2-tailed)	.008	.034	.002
	N	45	45	45
Yr 2 Renal MDE	Pearson Correlation	.192	.073	.273
	Sig. (2-tailed)	.207	.636	.070
	N	45	45	45
Yr 2 Infectious Diseases MDE	Pearson Correlation	.309*	.111	.299*
	Sig. (2-tailed)	.039	.468	.046
	N	45	45	45
Yr 1 Hematology MDE	Pearson Correlation	.059	.119	.189*
	Sig. (2-tailed)	.541	.219	.049
	N	109	109	109
Yr 1 Neurology MDE	Pearson Correlation	.398**	.336*	.463**
	Sig. (2-tailed)	.007	.024	.001
	N	45	45	45

Correlations

		(C6) Deliberation	MCAT Verbal	MCAT Biological Sciences
MCAT Biological Sciences	Pearson Correlation	.000	.022	1
	Sig. (2-tailed)	.998	.813	
	N	116	116	116
MCAT Physical Sciences	Pearson Correlation	-.033	.044	.260**
	Sig. (2-tailed)	.723	.641	.005
	N	116	116	116
Yr 1 Phase A MDE	Pearson Correlation	.116	.067	.410**
	Sig. (2-tailed)	.216	.476	.000
	N	115	115	115
Yr 1 Phase B MDE	Pearson Correlation	.143	.084	.312**
	Sig. (2-tailed)	.128	.370	.001
	N	115	115	115
Yr 1 Host Defense MDE	Pearson Correlation	.279**	-.098	.215*
	Sig. (2-tailed)	.003	.304	.022
	N	113	113	113
Yr 2 GI MDE	Pearson Correlation	.092	-.491**	.214
	Sig. (2-tailed)	.546	.001	.157
	N	45	45	45
Yr 2 Renal MDE	Pearson Correlation	.055	-.223	.288
	Sig. (2-tailed)	.721	.140	.055
	N	45	45	45
Yr 2 Infectious Diseases MDE	Pearson Correlation	.226	-.401**	.147
	Sig. (2-tailed)	.136	.006	.335
	N	45	45	45
Yr 1 Hematology MDE	Pearson Correlation	.095	.054	.166
	Sig. (2-tailed)	.325	.577	.085
	N	109	109	109
Yr 1 Neurology MDE	Pearson Correlation	.222	-.369*	.239
	Sig. (2-tailed)	.143	.013	.114
	N	45	45	45

Correlations

		MCAT Physical Sciences	Yr 1 Phase A MDE	Yr 1 Phase B MDE
MCAT Biological Sciences	Pearson Correlation	.260**	.410**	.312**
	Sig. (2-tailed)	.005	.000	.001
	N	116	115	115
MCAT Physical Sciences	Pearson Correlation	1	.274**	.209*
	Sig. (2-tailed)		.003	.025
	N	116	115	115
Yr 1 Phase A MDE	Pearson Correlation	.274**	1	.733**
	Sig. (2-tailed)	.003		.000
	N	115	115	115
Yr 1 Phase B MDE	Pearson Correlation	.209*	.733**	1
	Sig. (2-tailed)	.025	.000	
	N	115	115	115
Yr 1 Host Defense MDE	Pearson Correlation	.104	.705**	.722**
	Sig. (2-tailed)	.272	.000	.000
	N	113	113	113
Yr 2 GI MDE	Pearson Correlation	.088	.489**	.475**
	Sig. (2-tailed)	.566	.001	.001
	N	45	45	45
Yr 2 Renal MDE	Pearson Correlation	.136	.581**	.636**
	Sig. (2-tailed)	.371	.000	.000
	N	45	45	45
Yr 2 Infectious Diseases MDE	Pearson Correlation	-.051	.501**	.542**
	Sig. (2-tailed)	.741	.000	.000
	N	45	45	45
Yr 1 Hematology MDE	Pearson Correlation	.003	.526**	.526**
	Sig. (2-tailed)	.978	.000	.000
	N	109	109	109
Yr 1 Neurology MDE	Pearson Correlation	.066	.636**	.677**
	Sig. (2-tailed)	.668	.000	.000
	N	45	45	45

Correlations

		Yr 1 Host Defense MDE	Yr 2 GI MDE	Yr 2 Renal MDE
MCAT Biological Sciences	Pearson Correlation	.215*	.214	.288
	Sig. (2-tailed)	.022	.157	.055
	N	113	45	45
MCAT Physical Sciences	Pearson Correlation	.104	.088	.136
	Sig. (2-tailed)	.272	.566	.371
	N	113	45	45
Yr 1 Phase A MDE	Pearson Correlation	.705**	.489**	.581**
	Sig. (2-tailed)	.000	.001	.000
	N	113	45	45
Yr 1 Phase B MDE	Pearson Correlation	.722**	.475**	.636**
	Sig. (2-tailed)	.000	.001	.000
	N	113	45	45
Yr 1 Host Defense MDE	Pearson Correlation	1	.697**	.670**
	Sig. (2-tailed)		.000	.000
	N	113	45	45
Yr 2 GI MDE	Pearson Correlation	.697**	1	.630**
	Sig. (2-tailed)	.000		.000
	N	45	45	45
Yr 2 Renal MDE	Pearson Correlation	.670**	.630**	1
	Sig. (2-tailed)	.000	.000	
	N	45	45	45
Yr 2 Infectious Diseases MDE	Pearson Correlation	.581**	.742**	.652**
	Sig. (2-tailed)	.000	.000	.000
	N	45	44	44
Yr 1 Hematology MDE	Pearson Correlation	.650**	.620**	.687**
	Sig. (2-tailed)	.000	.000	.000
	N	109	45	45
Yr 1 Neurology MDE	Pearson Correlation	.748**	.679**	.654**
	Sig. (2-tailed)	.000	.000	.000
	N	45	45	45

Correlations

		Yr 2 Infectious Diseases MDE	Yr 1 Hematology MDE	Yr 1 Neurology MDE
MCAT Biological Sciences	Pearson Correlation	.147	.166	.239
	Sig. (2-tailed)	.335	.085	.114
	N	45	109	45
MCAT Physical Sciences	Pearson Correlation	-.051	.003	.066
	Sig. (2-tailed)	.741	.978	.668
	N	45	109	45
Yr 1 Phase A MDE	Pearson Correlation	.501**	.526**	.636**
	Sig. (2-tailed)	.000	.000	.000
	N	45	109	45
Yr 1 Phase B MDE	Pearson Correlation	.542**	.526**	.677**
	Sig. (2-tailed)	.000	.000	.000
	N	45	109	45
Yr 1 Host Defense MDE	Pearson Correlation	.581**	.650**	.748**
	Sig. (2-tailed)	.000	.000	.000
	N	45	109	45
Yr 2 GI MDE	Pearson Correlation	.742**	.620**	.679**
	Sig. (2-tailed)	.000	.000	.000
	N	44	45	45
Yr 2 Renal MDE	Pearson Correlation	.652**	.687**	.654**
	Sig. (2-tailed)	.000	.000	.000
	N	44	45	45
Yr 2 Infectious Diseases MDE	Pearson Correlation	1	.566**	.613**
	Sig. (2-tailed)		.000	.000
	N	45	44	44
Yr 1 Hematology MDE	Pearson Correlation	.566**	1	.785**
	Sig. (2-tailed)	.000		.000
	N	44	109	45
Yr 1 Neurology MDE	Pearson Correlation	.613**	.785**	1
	Sig. (2-tailed)	.000	.000	
	N	44	45	45

Correlations

		Yr 1 Brain & Behavior MDE	Yr 1 Musculoskeletal MDE	Yr 1 Community Epidimiology Study Grade
MCAT Biological Sciences	Pearson Correlation	.258*	.411**	.203*
	Sig. (2-tailed)	.023	.005	.030
	N	77	45	115
MCAT Physical Sciences	Pearson Correlation	.002	.071	.067
	Sig. (2-tailed)	.986	.644	.478
	N	77	45	115
Yr 1 Phase A MDE	Pearson Correlation	.527**	.664**	.246**
	Sig. (2-tailed)	.000	.000	.008
	N	77	45	115
Yr 1 Phase B MDE	Pearson Correlation	.569**	.557**	.351**
	Sig. (2-tailed)	.000	.000	.000
	N	77	45	115
Yr 1 Host Defense MDE	Pearson Correlation	.661**	.588**	.281**
	Sig. (2-tailed)	.000	.000	.003
	N	77	45	113
Yr 2 GI MDE	Pearson Correlation	.457**	.618**	-.021
	Sig. (2-tailed)	.002	.000	.891
	N	45	45	45
Yr 2 Renal MDE	Pearson Correlation	.553**	.703**	.304*
	Sig. (2-tailed)	.000	.000	.042
	N	45	45	45
Yr 2 Infectious Diseases MDE	Pearson Correlation	.497**	.623**	.107
	Sig. (2-tailed)	.001	.000	.483
	N	44	44	45
Yr 1 Hematology MDE	Pearson Correlation	.625**	.730**	.327**
	Sig. (2-tailed)	.000	.000	.001
	N	77	45	109
Yr 1 Neurology MDE	Pearson Correlation	.587**	.771**	.234
	Sig. (2-tailed)	.000	.000	.122
	N	45	45	45

Correlations

		Yr 2 Cardiology MDE	Yr 2 Biostatistics	Yr 2 Pulmonology MDE
MCAT Biological Sciences	Pearson Correlation	.202	.018	.364*
	Sig. (2-tailed)	.183	.906	.014
	N	45	45	45
MCAT Physical Sciences	Pearson Correlation	.138	.214	.225
	Sig. (2-tailed)	.366	.158	.138
	N	45	45	45
Yr 1 Phase A MDE	Pearson Correlation	.688**	.329*	.673**
	Sig. (2-tailed)	.000	.027	.000
	N	45	45	45
Yr 1 Phase B MDE	Pearson Correlation	.496**	.307*	.682**
	Sig. (2-tailed)	.001	.040	.000
	N	45	45	45
Yr 1 Host Defense MDE	Pearson Correlation	.642**	.216	.630**
	Sig. (2-tailed)	.000	.153	.000
	N	45	45	45
Yr 2 GI MDE	Pearson Correlation	.594**	.235	.666**
	Sig. (2-tailed)	.000	.120	.000
	N	45	45	45
Yr 2 Renal MDE	Pearson Correlation	.647**	.279	.805**
	Sig. (2-tailed)	.000	.063	.000
	N	45	45	45
Yr 2 Infectious Diseases MDE	Pearson Correlation	.470**	.197	.666**
	Sig. (2-tailed)	.001	.200	.000
	N	44	44	44
Yr 1 Hematology MDE	Pearson Correlation	.644**	.432**	.801**
	Sig. (2-tailed)	.000	.003	.000
	N	45	45	45
Yr 1 Neurology MDE	Pearson Correlation	.730**	.332*	.705**
	Sig. (2-tailed)	.000	.026	.000
	N	45	45	45

Correlations

		Yr 2 Endocrinology MDE	Yr 2 Research Design	Repeat Phase A MDE
MCAT Biological Sciences	Pearson Correlation	.217	-.013	-.400
	Sig. (2-tailed)	.148	.932	.286
	N	46	45	9
MCAT Physical Sciences	Pearson Correlation	.207	.050	.041
	Sig. (2-tailed)	.168	.745	.916
	N	46	45	9
Yr 1 Phase A MDE	Pearson Correlation	.557**	.297*	.496
	Sig. (2-tailed)	.000	.048	.175
	N	46	45	9
Yr 1 Phase B MDE	Pearson Correlation	.584**	.277	-.273
	Sig. (2-tailed)	.000	.065	.477
	N	46	45	9
Yr 1 Host Defense MDE	Pearson Correlation	.604**	.258	.676*
	Sig. (2-tailed)	.000	.087	.046
	N	46	45	9
Yr 2 GI MDE	Pearson Correlation	.672**	.057	.[c]
	Sig. (2-tailed)	.000	.710	.
	N	45	45	1
Yr 2 Renal MDE	Pearson Correlation	.795**	.191	.[c]
	Sig. (2-tailed)	.000	.210	.
	N	45	45	1
Yr 2 Infectious Diseases MDE	Pearson Correlation	.695**	.180	.[c]
	Sig. (2-tailed)	.000	.242	.
	N	45	44	0
Yr 1 Hematology MDE	Pearson Correlation	.648**	.370*	.471
	Sig. (2-tailed)	.000	.012	.201
	N	45	45	9
Yr 1 Neurology MDE	Pearson Correlation	.602**	.288	.[c]
	Sig. (2-tailed)	.000	.055	.
	N	45	45	1

Correlations

		Repeat Phase B MDE	Repeat Phase A SAP	Repeat Host Defense MDE
MCAT Biological Sciences	Pearson Correlation	-.102	-.573	-.561
	Sig. (2-tailed)	.794	.107	.116
	N	9	9	9
MCAT Physical Sciences	Pearson Correlation	-.381	.018	-.436
	Sig. (2-tailed)	.312	.962	.241
	N	9	9	9
Yr 1 Phase A MDE	Pearson Correlation	.225	.149	.184
	Sig. (2-tailed)	.561	.703	.635
	N	9	9	9
Yr 1 Phase B MDE	Pearson Correlation	-.023	-.091	-.283
	Sig. (2-tailed)	.954	.816	.461
	N	9	9	9
Yr 1 Host Defense MDE	Pearson Correlation	.302	.328	.563
	Sig. (2-tailed)	.429	.389	.115
	N	9	9	9
Yr 2 GI MDE	Pearson Correlation	.[c]	.[c]	.[c]
	Sig. (2-tailed)	.	.	.
	N	1	1	1
Yr 2 Renal MDE	Pearson Correlation	.[c]	.[c]	.[c]
	Sig. (2-tailed)	.	.	.
	N	1	1	1
Yr 2 Infectious Diseases MDE	Pearson Correlation	.[c]	.[c]	.[c]
	Sig. (2-tailed)	.	.	.
	N	0	0	0
Yr 1 Hematology MDE	Pearson Correlation	.335	.645	.415
	Sig. (2-tailed)	.377	.061	.266
	N	9	9	9
Yr 1 Neurology MDE	Pearson Correlation	.[c]	.[c]	.[c]
	Sig. (2-tailed)	.	.	.
	N	1	1	1

Correlations

		Repeat Hematology MDE	Repeat Neurology MDE	Repeat Brain & Behavior MDE
MCAT Biological Sciences	Pearson Correlation	.560	.387	-.155
	Sig. (2-tailed)	.117	.304	.690
	N	9	9	9
MCAT Physical Sciences	Pearson Correlation	.605	.433	-.468
	Sig. (2-tailed)	.085	.244	.204
	N	9	9	9
Yr 1 Phase A MDE	Pearson Correlation	.184	-.211	.215
	Sig. (2-tailed)	.635	.585	.579
	N	9	9	9
Yr 1 Phase B MDE	Pearson Correlation	-.018	.055	.607
	Sig. (2-tailed)	.962	.888	.083
	N	9	9	9
Yr 1 Host Defense MDE	Pearson Correlation	-.159	-.606	.518
	Sig. (2-tailed)	.683	.084	.153
	N	9	9	9
Yr 2 GI MDE	Pearson Correlation	.[c]	.[c]	.[c]
	Sig. (2-tailed)	.	.	.
	N	1	1	1
Yr 2 Renal MDE	Pearson Correlation	.[c]	.[c]	.[c]
	Sig. (2-tailed)	.	.	.
	N	1	1	1
Yr 2 Infectious Diseases MDE	Pearson Correlation	.[c]	.[c]	.[c]
	Sig. (2-tailed)	.	.	.
	N	0	0	0
Yr 1 Hematology MDE	Pearson Correlation	-.173	-.352	.521
	Sig. (2-tailed)	.656	.353	.150
	N	9	9	9
Yr 1 Neurology MDE	Pearson Correlation	.[c]	.[c]	.[c]
	Sig. (2-tailed)	.	.	.
	N	1	1	1

Correlations

		Repeat Musculoskeletal MDE
MCAT Biological Sciences	Pearson Correlation	-.278
	Sig. (2-tailed)	.469
	N	9
MCAT Physical Sciences	Pearson Correlation	-.070
	Sig. (2-tailed)	.859
	N	9
Yr 1 Phase A MDE	Pearson Correlation	-.068
	Sig. (2-tailed)	.862
	N	9
Yr 1 Phase B MDE	Pearson Correlation	.156
	Sig. (2-tailed)	.689
	N	9
Yr 1 Host Defense MDE	Pearson Correlation	.004
	Sig. (2-tailed)	.992
	N	9
Yr 2 GI MDE	Pearson Correlation	.[c]
	Sig. (2-tailed)	.
	N	1
Yr 2 Renal MDE	Pearson Correlation	.[c]
	Sig. (2-tailed)	.
	N	1
Yr 2 Infectious Diseases MDE	Pearson Correlation	.[c]
	Sig. (2-tailed)	.
	N	0
Yr 1 Hematology MDE	Pearson Correlation	.055
	Sig. (2-tailed)	.888
	N	9
Yr 1 Neurology MDE	Pearson Correlation	.[c]
	Sig. (2-tailed)	.
	N	1

Correlations

		(N) Neuroticism	(E) Extraversion	(O) Openness
Yr 1 Brain & Behavior MDE	Pearson Correlation	-.008	-.085	-.024
	Sig. (2-tailed)	.944	.462	.839
	N	77	77	77
Yr 1 Musculoskeletal MDE	Pearson Correlation	-.157	.077	-.034
	Sig. (2-tailed)	.301	.617	.823
	N	45	45	45
Yr 1 Community Epidimiology Study Grade	Pearson Correlation	-.050	-.194*	-.012
	Sig. (2-tailed)	.597	.037	.895
	N	115	115	115
Yr 2 Cardiology MDE	Pearson Correlation	-.232	.023	-.080
	Sig. (2-tailed)	.125	.878	.599
	N	45	45	45
Yr 2 Biostatistics	Pearson Correlation	.058	-.014	.128
	Sig. (2-tailed)	.706	.929	.403
	N	45	45	45
Yr 2 Pulmonology MDE	Pearson Correlation	-.190	.012	.079
	Sig. (2-tailed)	.211	.938	.604
	N	45	45	45
Yr 2 Endocrinology MDE	Pearson Correlation	-.023	.041	-.178
	Sig. (2-tailed)	.880	.788	.236
	N	46	46	46
Yr 2 Research Design	Pearson Correlation	.062	.065	.054
	Sig. (2-tailed)	.684	.670	.724
	N	45	45	45
Repeat Phase A MDE	Pearson Correlation	.107	.187	-.572
	Sig. (2-tailed)	.784	.630	.108
	N	9	9	9
Repeat Phase B MDE	Pearson Correlation	-.457	.541	.047
	Sig. (2-tailed)	.217	.133	.904
	N	9	9	9

Correlations

		(A) Agreeableness	(C) Conscientiousness	(N1) Anxiety
Yr 1 Brain & Behavior MDE	Pearson Correlation	.050	.154	-.088
	Sig. (2-tailed)	.665	.182	.447
	N	77	77	77
Yr 1 Musculoskeletal MDE	Pearson Correlation	.155	.243	-.202
	Sig. (2-tailed)	.310	.108	.183
	N	45	45	45
Yr 1 Community Epidimiology Study Grade	Pearson Correlation	-.147	-.069	-.049
	Sig. (2-tailed)	.116	.463	.606
	N	115	115	115
Yr 2 Cardiology MDE	Pearson Correlation	.080	.282	-.234
	Sig. (2-tailed)	.601	.060	.122
	N	45	45	45
Yr 2 Biostatistics	Pearson Correlation	-.031	.032	.110
	Sig. (2-tailed)	.840	.836	.473
	N	45	45	45
Yr 2 Pulmonology MDE	Pearson Correlation	.081	.158	-.222
	Sig. (2-tailed)	.596	.299	.143
	N	45	45	45
Yr 2 Endocrinology MDE	Pearson Correlation	.047	.112	-.087
	Sig. (2-tailed)	.757	.460	.567
	N	46	46	46
Yr 2 Research Design	Pearson Correlation	-.031	.063	.023
	Sig. (2-tailed)	.841	.679	.880
	N	45	45	45
Repeat Phase A MDE	Pearson Correlation	-.063	-.313	.359
	Sig. (2-tailed)	.872	.412	.342
	N	9	9	9
Repeat Phase B MDE	Pearson Correlation	-.193	-.054	-.381
	Sig. (2-tailed)	.620	.891	.311
	N	9	9	9

Correlations

		(N2) Angry Hostility	(N3) Depression	(N4) Self-Consciousness
Yr 1 Brain & Behavior MDE	Pearson Correlation	.009	-.049	.139
	Sig. (2-tailed)	.937	.670	.227
	N	77	77	77
Yr 1 Musculoskeletal MDE	Pearson Correlation	-.211	-.244	-.078
	Sig. (2-tailed)	.163	.107	.608
	N	45	45	45
Yr 1 Community Epidimiology Study Grade	Pearson Correlation	.022	-.022	.081
	Sig. (2-tailed)	.819	.818	.387
	N	115	115	115
Yr 2 Cardiology MDE	Pearson Correlation	-.234	-.277	-.157
	Sig. (2-tailed)	.122	.066	.303
	N	45	45	45
Yr 2 Biostatistics	Pearson Correlation	-.006	.051	-.017
	Sig. (2-tailed)	.967	.737	.910
	N	45	45	45
Yr 2 Pulmonology MDE	Pearson Correlation	-.186	-.205	-.023
	Sig. (2-tailed)	.221	.178	.881
	N	45	45	45
Yr 2 Endocrinology MDE	Pearson Correlation	.003	.003	.095
	Sig. (2-tailed)	.982	.984	.529
	N	46	46	46
Yr 2 Research Design	Pearson Correlation	.093	.060	.030
	Sig. (2-tailed)	.542	.694	.843
	N	45	45	45
Repeat Phase A MDE	Pearson Correlation	.151	.222	.013
	Sig. (2-tailed)	.698	.566	.973
	N	9	9	9
Repeat Phase B MDE	Pearson Correlation	-.282	-.542	-.484
	Sig. (2-tailed)	.462	.132	.187
	N	9	9	9

Correlations

		(N5) Impulsiveness	(N6) Vulnerability	(E1) Warmth
Yr 1 Brain & Behavior MDE	Pearson Correlation	.091	-.174	-.025
	Sig. (2-tailed)	.432	.131	.827
	N	77	77	77
Yr 1 Musculoskeletal MDE	Pearson Correlation	-.122	-.193	.129
	Sig. (2-tailed)	.424	.203	.398
	N	45	45	45
Yr 1 Community Epidimiology Study Grade	Pearson Correlation	.070	-.021	-.216*
	Sig. (2-tailed)	.457	.821	.021
	N	115	115	115
Yr 2 Cardiology MDE	Pearson Correlation	-.142	-.326*	.077
	Sig. (2-tailed)	.353	.029	.617
	N	45	45	45
Yr 2 Biostatistics	Pearson Correlation	.083	.000	-.022
	Sig. (2-tailed)	.587	.999	.888
	N	45	45	45
Yr 2 Pulmonology MDE	Pearson Correlation	-.044	-.286	.036
	Sig. (2-tailed)	.776	.057	.814
	N	45	45	45
Yr 2 Endocrinology MDE	Pearson Correlation	-.009	-.091	.091
	Sig. (2-tailed)	.954	.546	.547
	N	46	46	46
Yr 2 Research Design	Pearson Correlation	.061	-.149	.063
	Sig. (2-tailed)	.690	.329	.679
	N	45	45	45
Repeat Phase A MDE	Pearson Correlation	.116	.081	-.038
	Sig. (2-tailed)	.766	.836	.922
	N	9	9	9
Repeat Phase B MDE	Pearson Correlation	.304	-.113	.021
	Sig. (2-tailed)	.426	.773	.957
	N	9	9	9

Correlations

		(E2) Gregariousness	(E3) Assertiveness	(E4) Activity
Yr 1 Brain & Behavior MDE	Pearson Correlation	-.136	-.072	.017
	Sig. (2-tailed)	.238	.531	.884
	N	77	77	77
Yr 1 Musculoskeletal MDE	Pearson Correlation	-.068	-.028	.065
	Sig. (2-tailed)	.658	.855	.672
	N	45	45	45
Yr 1 Community Epidimiology Study Grade	Pearson Correlation	-.201*	-.136	-.128
	Sig. (2-tailed)	.031	.147	.173
	N	115	115	115
Yr 2 Cardiology MDE	Pearson Correlation	-.085	.065	.139
	Sig. (2-tailed)	.581	.672	.361
	N	45	45	45
Yr 2 Biostatistics	Pearson Correlation	.002	.040	.059
	Sig. (2-tailed)	.992	.792	.699
	N	45	45	45
Yr 2 Pulmonology MDE	Pearson Correlation	-.108	-.036	.112
	Sig. (2-tailed)	.480	.815	.465
	N	45	45	45
Yr 2 Endocrinology MDE	Pearson Correlation	-.016	-.046	.172
	Sig. (2-tailed)	.913	.759	.253
	N	46	46	46
Yr 2 Research Design	Pearson Correlation	.007	.109	.144
	Sig. (2-tailed)	.963	.478	.345
	N	45	45	45
Repeat Phase A MDE	Pearson Correlation	-.014	-.396	.092
	Sig. (2-tailed)	.971	.291	.814
	N	9	9	9
Repeat Phase B MDE	Pearson Correlation	.532	.293	.458
	Sig. (2-tailed)	.141	.443	.215
	N	9	9	9

Correlations

		(E5) Excitement-Seeking	(E6) Positive Emotions	(O1) Fantasy
Yr 1 Brain & Behavior MDE	Pearson Correlation	-.078	.093	-.048
	Sig. (2-tailed)	.502	.420	.676
	N	77	77	77
Yr 1 Musculoskeletal MDE	Pearson Correlation	-.012	.284	-.190
	Sig. (2-tailed)	.940	.058	.212
	N	45	45	45
Yr 1 Community Epidimiology Study Grade	Pearson Correlation	-.041	-.097	-.057
	Sig. (2-tailed)	.664	.302	.548
	N	115	115	115
Yr 2 Cardiology MDE	Pearson Correlation	-.081	.135	-.183
	Sig. (2-tailed)	.596	.377	.229
	N	45	45	45
Yr 2 Biostatistics	Pearson Correlation	-.023	.050	-.161
	Sig. (2-tailed)	.878	.743	.291
	N	45	45	45
Yr 2 Pulmonology MDE	Pearson Correlation	-.047	.261	-.127
	Sig. (2-tailed)	.757	.083	.404
	N	45	45	45
Yr 2 Endocrinology MDE	Pearson Correlation	-.120	.167	-.168
	Sig. (2-tailed)	.425	.267	.264
	N	46	46	46
Yr 2 Research Design	Pearson Correlation	.044	.070	-.165
	Sig. (2-tailed)	.775	.648	.277
	N	45	45	45
Repeat Phase A MDE	Pearson Correlation	-.243	.059	-.039
	Sig. (2-tailed)	.528	.881	.920
	N	9	9	9
Repeat Phase B MDE	Pearson Correlation	.894**	-.230	-.210
	Sig. (2-tailed)	.001	.552	.587
	N	9	9	9

Correlations

		(O2) Aesthetics	(O3) Feelings	(O4) Actions
Yr 1 Brain & Behavior MDE	Pearson Correlation	.027	-.204	-.077
	Sig. (2-tailed)	.816	.075	.506
	N	77	77	77
Yr 1 Musculoskeletal MDE	Pearson Correlation	.016	-.102	-.152
	Sig. (2-tailed)	.915	.507	.318
	N	45	45	45
Yr 1 Community Epidimiology Study Grade	Pearson Correlation	.000	-.091	.022
	Sig. (2-tailed)	.998	.332	.819
	N	115	115	115
Yr 2 Cardiology MDE	Pearson Correlation	-.087	-.181	-.171
	Sig. (2-tailed)	.568	.235	.262
	N	45	45	45
Yr 2 Biostatistics	Pearson Correlation	.119	.151	.145
	Sig. (2-tailed)	.438	.322	.343
	N	45	45	45
Yr 2 Pulmonology MDE	Pearson Correlation	.094	-.123	.084
	Sig. (2-tailed)	.541	.420	.584
	N	45	45	45
Yr 2 Endocrinology MDE	Pearson Correlation	-.073	-.263	-.034
	Sig. (2-tailed)	.628	.077	.822
	N	46	46	46
Yr 2 Research Design	Pearson Correlation	-.022	.239	.076
	Sig. (2-tailed)	.884	.113	.621
	N	45	45	45
Repeat Phase A MDE	Pearson Correlation	-.553	-.432	-.051
	Sig. (2-tailed)	.123	.246	.896
	N	9	9	9
Repeat Phase B MDE	Pearson Correlation	.265	-.165	.274
	Sig. (2-tailed)	.490	.670	.476
	N	9	9	9

Correlations

		(O5) Ideas	(O6) Values	(A1) Trust	(A2) Straightforwardness
Yr 1 Brain & Behavior MDE	Pearson Correlation	-.011	.083	.008	.076
	Sig. (2-tailed)	.927	.474	.944	.511
	N	77	77	77	77
Yr 1 Musculoskeletal MDE	Pearson Correlation	.145	.093	.117	.109
	Sig. (2-tailed)	.341	.545	.445	.478
	N	45	45	45	45
Yr 1 Community Epidimiology Study Grade	Pearson Correlation	-.087	.067	-.109	-.155
	Sig. (2-tailed)	.353	.474	.247	.098
	N	115	115	115	115
Yr 2 Cardiology MDE	Pearson Correlation	.064	.120	.044	.059
	Sig. (2-tailed)	.675	.432	.773	.700
	N	45	45	45	45
Yr 2 Biostatistics	Pearson Correlation	.091	.254	-.199	-.064
	Sig. (2-tailed)	.553	.092	.189	.677
	N	45	45	45	45
Yr 2 Pulmonology MDE	Pearson Correlation	.110	.128	.059	.034
	Sig. (2-tailed)	.472	.401	.699	.826
	N	45	45	45	45
Yr 2 Endocrinology MDE	Pearson Correlation	-.157	-.121	-.019	.050
	Sig. (2-tailed)	.297	.422	.902	.742
	N	46	46	46	46
Yr 2 Research Design	Pearson Correlation	.064	.211	-.191	-.044
	Sig. (2-tailed)	.677	.164	.209	.775
	N	45	45	45	45
Repeat Phase A MDE	Pearson Correlation	-.757*	-.722*	-.248	-.159
	Sig. (2-tailed)	.018	.028	.520	.683
	N	9	9	9	9
Repeat Phase B MDE	Pearson Correlation	.398	.303	.473	.233
	Sig. (2-tailed)	.289	.428	.198	.546
	N	9	9	9	9

Correlations

		(A3) Altruism	(A4) Compliance	(A5) Modesty
Yr 1 Brain & Behavior MDE	Pearson Correlation	-.014	.216	-.009
	Sig. (2-tailed)	.906	.059	.937
	N	77	77	77
Yr 1 Musculoskeletal MDE	Pearson Correlation	.178	.205	.012
	Sig. (2-tailed)	.243	.177	.940
	N	45	45	45
Yr 1 Community Epidimiology Study Grade	Pearson Correlation	-.196*	-.018	-.040
	Sig. (2-tailed)	.036	.851	.668
	N	115	115	115
Yr 2 Cardiology MDE	Pearson Correlation	.100	.205	-.010
	Sig. (2-tailed)	.514	.176	.947
	N	45	45	45
Yr 2 Biostatistics	Pearson Correlation	-.044	-.022	.135
	Sig. (2-tailed)	.776	.888	.378
	N	45	45	45
Yr 2 Pulmonology MDE	Pearson Correlation	.042	.167	-.074
	Sig. (2-tailed)	.785	.271	.630
	N	45	45	45
Yr 2 Endocrinology MDE	Pearson Correlation	-.031	.173	-.100
	Sig. (2-tailed)	.838	.250	.511
	N	46	46	46
Yr 2 Research Design	Pearson Correlation	-.049	-.024	.228
	Sig. (2-tailed)	.751	.874	.132
	N	45	45	45
Repeat Phase A MDE	Pearson Correlation	-.055	.138	-.184
	Sig. (2-tailed)	.888	.724	.636
	N	9	9	9
Repeat Phase B MDE	Pearson Correlation	.058	.138	-.538
	Sig. (2-tailed)	.882	.723	.135
	N	9	9	9

Correlations

		(A6) Tender-Mindedness	(C1) Competence	(C2) Order
Yr 1 Brain & Behavior MDE	Pearson Correlation	-.065	.165	.146
	Sig. (2-tailed)	.574	.150	.206
	N	77	77	77
Yr 1 Musculoskeletal MDE	Pearson Correlation	.081	.250	.141
	Sig. (2-tailed)	.595	.097	.355
	N	45	45	45
Yr 1 Community Epidimiology Study Grade	Pearson Correlation	-.212*	.058	-.023
	Sig. (2-tailed)	.023	.540	.810
	N	115	115	115
Yr 2 Cardiology MDE	Pearson Correlation	.101	.249	.117
	Sig. (2-tailed)	.507	.099	.443
	N	45	45	45
Yr 2 Biostatistics	Pearson Correlation	-.026	-.027	-.102
	Sig. (2-tailed)	.864	.859	.505
	N	45	45	45
Yr 2 Pulmonology MDE	Pearson Correlation	.069	.224	.025
	Sig. (2-tailed)	.652	.140	.870
	N	45	45	45
Yr 2 Endocrinology MDE	Pearson Correlation	.026	.075	.024
	Sig. (2-tailed)	.866	.619	.874
	N	46	46	46
Yr 2 Research Design	Pearson Correlation	.056	.051	-.056
	Sig. (2-tailed)	.717	.740	.717
	N	45	45	45
Repeat Phase A MDE	Pearson Correlation	.060	-.131	.088
	Sig. (2-tailed)	.879	.737	.823
	N	9	9	9
Repeat Phase B MDE	Pearson Correlation	-.025	.372	.456
	Sig. (2-tailed)	.950	.324	.218
	N	9	9	9

Correlations

		(C3) Dutifulness	(C4) Achievement Striving	(C5) Self-Discipline
Yr 1 Brain & Behavior MDE	Pearson Correlation	.109	.023	.140
	Sig. (2-tailed)	.347	.843	.224
	N	77	77	77
Yr 1 Musculoskeletal MDE	Pearson Correlation	.316*	.154	.388**
	Sig. (2-tailed)	.034	.312	.008
	N	45	45	45
Yr 1 Community Epidimiology Study Grade	Pearson Correlation	-.094	-.159	-.042
	Sig. (2-tailed)	.319	.091	.653
	N	115	115	115
Yr 2 Cardiology MDE	Pearson Correlation	.343*	.301*	.466**
	Sig. (2-tailed)	.021	.044	.001
	N	45	45	45
Yr 2 Biostatistics	Pearson Correlation	.039	.029	.128
	Sig. (2-tailed)	.797	.850	.404
	N	45	45	45
Yr 2 Pulmonology MDE	Pearson Correlation	.268	.042	.328*
	Sig. (2-tailed)	.075	.786	.028
	N	45	45	45
Yr 2 Endocrinology MDE	Pearson Correlation	.103	-.033	.221
	Sig. (2-tailed)	.497	.829	.140
	N	46	46	46
Yr 2 Research Design	Pearson Correlation	.022	.059	.081
	Sig. (2-tailed)	.888	.699	.597
	N	45	45	45
Repeat Phase A MDE	Pearson Correlation	-.206	-.378	-.404
	Sig. (2-tailed)	.595	.316	.280
	N	9	9	9
Repeat Phase B MDE	Pearson Correlation	-.128	.134	.147
	Sig. (2-tailed)	.743	.731	.705
	N	9	9	9

Correlations

		(C6) Deliberation	MCAT Verbal	MCAT Biological Sciences
Yr 1 Brain & Behavior MDE	Pearson Correlation	.166	-.002	.258*
	Sig. (2-tailed)	.150	.984	.023
	N	77	77	77
Yr 1 Musculoskeletal MDE	Pearson Correlation	.056	-.269	.411**
	Sig. (2-tailed)	.715	.074	.005
	N	45	45	45
Yr 1 Community Epidimiology Study Grade	Pearson Correlation	-.026	.323**	.203*
	Sig. (2-tailed)	.780	.000	.030
	N	115	115	115
Yr 2 Cardiology MDE	Pearson Correlation	.065	-.196	.202
	Sig. (2-tailed)	.671	.196	.183
	N	45	45	45
Yr 2 Biostatistics	Pearson Correlation	-.079	.060	.018
	Sig. (2-tailed)	.607	.696	.906
	N	45	45	45
Yr 2 Pulmonology MDE	Pearson Correlation	.036	-.099	.364*
	Sig. (2-tailed)	.814	.519	.014
	N	45	45	45
Yr 2 Endocrinology MDE	Pearson Correlation	.090	-.200	.217
	Sig. (2-tailed)	.554	.183	.148
	N	46	46	46
Yr 2 Research Design	Pearson Correlation	-.042	.279	-.013
	Sig. (2-tailed)	.786	.064	.932
	N	45	45	45
Repeat Phase A MDE	Pearson Correlation	-.252	-.132	-.400
	Sig. (2-tailed)	.513	.735	.286
	N	9	9	9
Repeat Phase B MDE	Pearson Correlation	-.573	.261	-.102
	Sig. (2-tailed)	.106	.497	.794
	N	9	9	9

Correlations

		MCAT Physical Sciences	Yr 1 Phase A MDE	Yr 1 Phase B MDE
Yr 1 Brain & Behavior MDE	Pearson Correlation	.002	.527**	.569**
	Sig. (2-tailed)	.986	.000	.000
	N	77	77	77
Yr 1 Musculoskeletal MDE	Pearson Correlation	.071	.664**	.557**
	Sig. (2-tailed)	.644	.000	.000
	N	45	45	45
Yr 1 Community Epidimiology Study Grade	Pearson Correlation	.067	.246**	.351**
	Sig. (2-tailed)	.478	.008	.000
	N	115	115	115
Yr 2 Cardiology MDE	Pearson Correlation	.138	.688**	.496**
	Sig. (2-tailed)	.366	.000	.001
	N	45	45	45
Yr 2 Biostatistics	Pearson Correlation	.214	.329*	.307*
	Sig. (2-tailed)	.158	.027	.040
	N	45	45	45
Yr 2 Pulmonology MDE	Pearson Correlation	.225	.673**	.682**
	Sig. (2-tailed)	.138	.000	.000
	N	45	45	45
Yr 2 Endocrinology MDE	Pearson Correlation	.207	.557**	.584**
	Sig. (2-tailed)	.168	.000	.000
	N	46	46	46
Yr 2 Research Design	Pearson Correlation	.050	.297*	.277
	Sig. (2-tailed)	.745	.048	.065
	N	45	45	45
Repeat Phase A MDE	Pearson Correlation	.041	.496	-.273
	Sig. (2-tailed)	.916	.175	.477
	N	9	9	9
Repeat Phase B MDE	Pearson Correlation	-.381	.225	-.023
	Sig. (2-tailed)	.312	.561	.954
	N	9	9	9

Correlations

		Yr 1 Host Defense MDE	Yr 2 GI MDE	Yr 2 Renal MDE
Yr 1 Brain & Behavior MDE	Pearson Correlation	.661**	.457**	.553**
	Sig. (2-tailed)	.000	.002	.000
	N	77	45	45
Yr 1 Musculoskeletal MDE	Pearson Correlation	.588**	.618**	.703**
	Sig. (2-tailed)	.000	.000	.000
	N	45	45	45
Yr 1 Community Epidimiology Study Grade	Pearson Correlation	.281**	-.021	.304*
	Sig. (2-tailed)	.003	.891	.042
	N	113	45	45
Yr 2 Cardiology MDE	Pearson Correlation	.642**	.594**	.647**
	Sig. (2-tailed)	.000	.000	.000
	N	45	45	45
Yr 2 Biostatistics	Pearson Correlation	.216	.235	.279
	Sig. (2-tailed)	.153	.120	.063
	N	45	45	45
Yr 2 Pulmonology MDE	Pearson Correlation	.630**	.666**	.805**
	Sig. (2-tailed)	.000	.000	.000
	N	45	45	45
Yr 2 Endocrinology MDE	Pearson Correlation	.604**	.672**	.795**
	Sig. (2-tailed)	.000	.000	.000
	N	46	45	45
Yr 2 Research Design	Pearson Correlation	.258	.057	.191
	Sig. (2-tailed)	.087	.710	.210
	N	45	45	45
Repeat Phase A MDE	Pearson Correlation	.676*	.c	.c
	Sig. (2-tailed)	.046	.	.
	N	9	1	1
Repeat Phase B MDE	Pearson Correlation	.302	.c	.c
	Sig. (2-tailed)	.429	.	.
	N	9	1	1

Correlations

		Yr 2 Infectious Diseases MDE	Yr 1 Hematology MDE	Yr 1 Neurology MDE
Yr 1 Brain & Behavior MDE	Pearson Correlation	.497**	.625**	.587**
	Sig. (2-tailed)	.001	.000	.000
	N	44	77	45
Yr 1 Musculoskeletal MDE	Pearson Correlation	.623**	.730**	.771**
	Sig. (2-tailed)	.000	.000	.000
	N	44	45	45
Yr 1 Community Epidimiology Study Grade	Pearson Correlation	.107	.327**	.234
	Sig. (2-tailed)	.483	.001	.122
	N	45	109	45
Yr 2 Cardiology MDE	Pearson Correlation	.470**	.644**	.730**
	Sig. (2-tailed)	.001	.000	.000
	N	44	45	45
Yr 2 Biostatistics	Pearson Correlation	.197	.432**	.332*
	Sig. (2-tailed)	.200	.003	.026
	N	44	45	45
Yr 2 Pulmonology MDE	Pearson Correlation	.666**	.801**	.705**
	Sig. (2-tailed)	.000	.000	.000
	N	44	45	45
Yr 2 Endocrinology MDE	Pearson Correlation	.695**	.648**	.602**
	Sig. (2-tailed)	.000	.000	.000
	N	45	45	45
Yr 2 Research Design	Pearson Correlation	.180	.370*	.288
	Sig. (2-tailed)	.242	.012	.055
	N	44	45	45
Repeat Phase A MDE	Pearson Correlation	.[c]	.471	.[c]
	Sig. (2-tailed)	.	.201	.
	N	0	9	1
Repeat Phase B MDE	Pearson Correlation	.[c]	.335	.[c]
	Sig. (2-tailed)	.	.377	.
	N	0	9	1

Correlations

		Yr 1 Brain & Behavior MDE	Yr 1 Musculoskeletal MDE	Yr 1 Community Epidimiology Study Grade
Yr 1 Brain & Behavior MDE	Pearson Correlation	1	.511**	.320**
	Sig. (2-tailed)		.000	.005
	N	77	45	77
Yr 1 Musculoskeletal MDE	Pearson Correlation	.511**	1	.003
	Sig. (2-tailed)	.000		.986
	N	45	45	45
Yr 1 Community Epidimiology Study Grade	Pearson Correlation	.320**	.003	1
	Sig. (2-tailed)	.005	.986	
	N	77	45	115
Yr 2 Cardiology MDE	Pearson Correlation	.376*	.775**	.115
	Sig. (2-tailed)	.011	.000	.450
	N	45	45	45
Yr 2 Biostatistics	Pearson Correlation	.213	.227	.474**
	Sig. (2-tailed)	.159	.133	.001
	N	45	45	45
Yr 2 Pulmonology MDE	Pearson Correlation	.672**	.755**	.321*
	Sig. (2-tailed)	.000	.000	.032
	N	45	45	45
Yr 2 Endocrinology MDE	Pearson Correlation	.602**	.593**	.154
	Sig. (2-tailed)	.000	.000	.306
	N	45	45	46
Yr 2 Research Design	Pearson Correlation	.274	.167	.418**
	Sig. (2-tailed)	.069	.273	.004
	N	45	45	45
Repeat Phase A MDE	Pearson Correlation	.276	.c	-.253
	Sig. (2-tailed)	.653	.	.511
	N	5	1	9
Repeat Phase B MDE	Pearson Correlation	.418	.c	.490
	Sig. (2-tailed)	.484	.	.180
	N	5	1	9

Correlations

		Yr 2 Cardiology MDE	Yr 2 Biostatistics	Yr 2 Pulmonology MDE
Yr 1 Brain & Behavior MDE	Pearson Correlation	.376*	.213	.672**
	Sig. (2-tailed)	.011	.159	.000
	N	45	45	45
Yr 1 Musculoskeletal MDE	Pearson Correlation	.775**	.227	.755**
	Sig. (2-tailed)	.000	.133	.000
	N	45	45	45
Yr 1 Community Epidimiology Study Grade	Pearson Correlation	.115	.474**	.321*
	Sig. (2-tailed)	.450	.001	.032
	N	45	45	45
Yr 2 Cardiology MDE	Pearson Correlation	1	.296*	.712**
	Sig. (2-tailed)		.048	.000
	N	45	45	45
Yr 2 Biostatistics	Pearson Correlation	.296*	1	.488**
	Sig. (2-tailed)	.048		.001
	N	45	45	45
Yr 2 Pulmonology MDE	Pearson Correlation	.712**	.488**	1
	Sig. (2-tailed)	.000	.001	
	N	45	45	45
Yr 2 Endocrinology MDE	Pearson Correlation	.639**	.277	.761**
	Sig. (2-tailed)	.000	.065	.000
	N	45	45	45
Yr 2 Research Design	Pearson Correlation	.318*	.574**	.399**
	Sig. (2-tailed)	.033	.000	.007
	N	45	45	45
Repeat Phase A MDE	Pearson Correlation	.[c]	.[c]	.[c]
	Sig. (2-tailed)	.	.	.
	N	1	1	1
Repeat Phase B MDE	Pearson Correlation	.[c]	.[c]	.[c]
	Sig. (2-tailed)	.	.	.
	N	1	1	1

Correlations

		Yr 2 Endocrinology MDE	Yr 2 Research Design	Repeat Phase A MDE
Yr 1 Brain & Behavior MDE	Pearson Correlation	.602**	.274	.276
	Sig. (2-tailed)	.000	.069	.653
	N	45	45	5
Yr 1 Musculoskeletal MDE	Pearson Correlation	.593**	.167	.c
	Sig. (2-tailed)	.000	.273	.
	N	45	45	1
Yr 1 Community Epidimiology Study Grade	Pearson Correlation	.154	.418**	-.253
	Sig. (2-tailed)	.306	.004	.511
	N	46	45	9
Yr 2 Cardiology MDE	Pearson Correlation	.639**	.318*	.c
	Sig. (2-tailed)	.000	.033	.
	N	45	45	1
Yr 2 Biostatistics	Pearson Correlation	.277	.574**	.c
	Sig. (2-tailed)	.065	.000	.
	N	45	45	1
Yr 2 Pulmonology MDE	Pearson Correlation	.761**	.399**	.c
	Sig. (2-tailed)	.000	.007	.
	N	45	45	1
Yr 2 Endocrinology MDE	Pearson Correlation	1	.204	.c
	Sig. (2-tailed)		.179	.
	N	46	45	1
Yr 2 Research Design	Pearson Correlation	.204	1	.c
	Sig. (2-tailed)	.179		.
	N	45	45	1
Repeat Phase A MDE	Pearson Correlation	.c	.c	1
	Sig. (2-tailed)	.	.	
	N	1	1	9
Repeat Phase B MDE	Pearson Correlation	.c	.c	-.035
	Sig. (2-tailed)	.	.	.929
	N	1	1	9

Correlations

		Repeat Phase B MDE	Repeat Phase A SAP	Repeat Host Defense MDE
Yr 1 Brain & Behavior MDE	Pearson Correlation	.418	-.499	.259
	Sig. (2-tailed)	.484	.392	.674
	N	5	5	5
Yr 1 Musculoskeletal MDE	Pearson Correlation	.[c]	.[c]	.[c]
	Sig. (2-tailed)	.	.	.
	N	1	1	1
Yr 1 Community Epidimiology Study Grade	Pearson Correlation	.490	.019	-.061
	Sig. (2-tailed)	.180	.961	.877
	N	9	9	9
Yr 2 Cardiology MDE	Pearson Correlation	.[c]	.[c]	.[c]
	Sig. (2-tailed)	.	.	.
	N	1	1	1
Yr 2 Biostatistics	Pearson Correlation	.[c]	.[c]	.[c]
	Sig. (2-tailed)	.	.	.
	N	1	1	1
Yr 2 Pulmonology MDE	Pearson Correlation	.[c]	.[c]	.[c]
	Sig. (2-tailed)	.	.	.
	N	1	1	1
Yr 2 Endocrinology MDE	Pearson Correlation	.[c]	.[c]	.[c]
	Sig. (2-tailed)	.	.	.
	N	1	1	1
Yr 2 Research Design	Pearson Correlation	.[c]	.[c]	.[c]
	Sig. (2-tailed)	.	.	.
	N	1	1	1
Repeat Phase A MDE	Pearson Correlation	-.035	.305	.767*
	Sig. (2-tailed)	.929	.425	.016
	N	9	9	9
Repeat Phase B MDE	Pearson Correlation	1	.193	.102
	Sig. (2-tailed)		.620	.793
	N	9	9	9

Correlations

		Repeat Hematology MDE	Repeat Neurology MDE	Repeat Brain & Behavior MDE
Yr 1 Brain & Behavior MDE	Pearson Correlation	.285	-.477	.652
	Sig. (2-tailed)	.642	.417	.233
	N	5	5	5
Yr 1 Musculoskeletal MDE	Pearson Correlation	.[c]	.[c]	.[c]
	Sig. (2-tailed)	.	.	.
	N	1	1	1
Yr 1 Community Epidimiology Study Grade	Pearson Correlation	-.308	-.091	.351
	Sig. (2-tailed)	.420	.816	.355
	N	9	9	9
Yr 2 Cardiology MDE	Pearson Correlation	.[c]	.[c]	.[c]
	Sig. (2-tailed)	.	.	.
	N	1	1	1
Yr 2 Biostatistics	Pearson Correlation	.[c]	.[c]	.[c]
	Sig. (2-tailed)	.	.	.
	N	1	1	1
Yr 2 Pulmonology MDE	Pearson Correlation	.[c]	.[c]	.[c]
	Sig. (2-tailed)	.	.	.
	N	1	1	1
Yr 2 Endocrinology MDE	Pearson Correlation	.[c]	.[c]	.[c]
	Sig. (2-tailed)	.	.	.
	N	1	1	1
Yr 2 Research Design	Pearson Correlation	.[c]	.[c]	.[c]
	Sig. (2-tailed)	.	.	.
	N	1	1	1
Repeat Phase A MDE	Pearson Correlation	-.210	-.625	-.137
	Sig. (2-tailed)	.588	.072	.725
	N	9	9	9
Repeat Phase B MDE	Pearson Correlation	.190	.039	.103
	Sig. (2-tailed)	.625	.921	.793
	N	9	9	9

Correlations

		Repeat Musculoskeletal MDE
Yr 1 Brain & Behavior MDE	Pearson Correlation	-.075
	Sig. (2-tailed)	.905
	N	5
Yr 1 Musculoskeletal MDE	Pearson Correlation	.[c]
	Sig. (2-tailed)	.
	N	1
Yr 1 Community Epidimiology Study Grade	Pearson Correlation	-.616
	Sig. (2-tailed)	.078
	N	9
Yr 2 Cardiology MDE	Pearson Correlation	.[c]
	Sig. (2-tailed)	.
	N	1
Yr 2 Biostatistics	Pearson Correlation	.[c]
	Sig. (2-tailed)	.
	N	1
Yr 2 Pulmonology MDE	Pearson Correlation	.[c]
	Sig. (2-tailed)	.
	N	1
Yr 2 Endocrinology MDE	Pearson Correlation	.[c]
	Sig. (2-tailed)	.
	N	1
Yr 2 Research Design	Pearson Correlation	.[c]
	Sig. (2-tailed)	.
	N	1
Repeat Phase A MDE	Pearson Correlation	.100
	Sig. (2-tailed)	.797
	N	9
Repeat Phase B MDE	Pearson Correlation	-.453
	Sig. (2-tailed)	.220
	N	9

Correlations

		(N) Neuroticism	(E) Extraversion	(O) Openness
Repeat Phase A SAP	Pearson Correlation	-.281	.247	-.046
	Sig. (2-tailed)	.463	.522	.907
	N	9	9	9
Repeat Host Defense MDE	Pearson Correlation	-.115	.216	-.798**
	Sig. (2-tailed)	.768	.577	.010
	N	9	9	9
Repeat Hematology MDE	Pearson Correlation	.123	.177	.543
	Sig. (2-tailed)	.753	.648	.131
	N	9	9	9
Repeat Neurology MDE	Pearson Correlation	.047	-.058	.717*
	Sig. (2-tailed)	.904	.881	.030
	N	9	9	9
Repeat Brain & Behavior MDE	Pearson Correlation	-.456	.282	-.001
	Sig. (2-tailed)	.217	.462	.997
	N	9	9	9
Repeat Musculoskeletal MDE	Pearson Correlation	-.381	.341	-.506
	Sig. (2-tailed)	.311	.369	.165
	N	9	9	9

Correlations

		(A) Agreeableness	(C) Conscientiousness	(N1) Anxiety
Repeat Phase A SAP	Pearson Correlation	-.096	.311	-.217
	Sig. (2-tailed)	.807	.415	.575
	N	9	9	9
Repeat Host Defense MDE	Pearson Correlation	.018	-.127	.276
	Sig. (2-tailed)	.962	.745	.473
	N	9	9	9
Repeat Hematology MDE	Pearson Correlation	.213	-.437	.029
	Sig. (2-tailed)	.583	.240	.940
	N	9	9	9
Repeat Neurology MDE	Pearson Correlation	-.205	.030	-.365
	Sig. (2-tailed)	.597	.939	.334
	N	9	9	9
Repeat Brain & Behavior MDE	Pearson Correlation	.462	.698*	-.294
	Sig. (2-tailed)	.211	.037	.442
	N	9	9	9
Repeat Musculoskeletal MDE	Pearson Correlation	.622	.234	-.232
	Sig. (2-tailed)	.074	.544	.547
	N	9	9	9

Correlations

		(N2) Angry Hostility	(N3) Depression	(N4) Self-Consciousness
Repeat Phase A SAP	Pearson Correlation	-.191	-.339	-.374
	Sig. (2-tailed)	.622	.371	.321
	N	9	9	9
Repeat Host Defense MDE	Pearson Correlation	-.067	.053	-.217
	Sig. (2-tailed)	.865	.893	.575
	N	9	9	9
Repeat Hematology MDE	Pearson Correlation	.098	-.091	.219
	Sig. (2-tailed)	.801	.817	.572
	N	9	9	9
Repeat Neurology MDE	Pearson Correlation	.112	-.010	.181
	Sig. (2-tailed)	.774	.979	.642
	N	9	9	9
Repeat Brain & Behavior MDE	Pearson Correlation	-.666	-.620	-.574
	Sig. (2-tailed)	.050	.075	.106
	N	9	9	9
Repeat Musculoskeletal MDE	Pearson Correlation	-.481	-.162	-.318
	Sig. (2-tailed)	.190	.677	.404
	N	9	9	9

Correlations

		(N5) Impulsiveness	(N6) Vulnerability	(E1) Warmth
Repeat Phase A SAP	Pearson Correlation	-.353	-.575	.146
	Sig. (2-tailed)	.352	.105	.708
	N	9	9	9
Repeat Host Defense MDE	Pearson Correlation	.110	-.013	.092
	Sig. (2-tailed)	.779	.973	.814
	N	9	9	9
Repeat Hematology MDE	Pearson Correlation	.480	.289	-.024
	Sig. (2-tailed)	.191	.451	.951
	N	9	9	9
Repeat Neurology MDE	Pearson Correlation	-.061	-.013	-.192
	Sig. (2-tailed)	.877	.974	.621
	N	9	9	9
Repeat Brain & Behavior MDE	Pearson Correlation	-.479	-.564	.638
	Sig. (2-tailed)	.192	.113	.065
	N	9	9	9
Repeat Musculoskeletal MDE	Pearson Correlation	-.501	-.412	.623
	Sig. (2-tailed)	.169	.271	.073
	N	9	9	9

Correlations

		(E2) Gregariousness	(E3) Assertiveness	(E4) Activity
Repeat Phase A SAP	Pearson Correlation	.024	.265	.603
	Sig. (2-tailed)	.951	.491	.086
	N	9	9	9
Repeat Host Defense MDE	Pearson Correlation	.207	-.200	-.082
	Sig. (2-tailed)	.592	.605	.834
	N	9	9	9
Repeat Hematology MDE	Pearson Correlation	.073	-.418	.238
	Sig. (2-tailed)	.853	.263	.538
	N	9	9	9
Repeat Neurology MDE	Pearson Correlation	-.071	.277	.282
	Sig. (2-tailed)	.856	.470	.463
	N	9	9	9
Repeat Brain & Behavior MDE	Pearson Correlation	.263	.443	.223
	Sig. (2-tailed)	.494	.233	.565
	N	9	9	9
Repeat Musculoskeletal MDE	Pearson Correlation	.438	.097	.130
	Sig. (2-tailed)	.238	.805	.738
	N	9	9	9

Correlations

		(E5) Excitement-Seeking	(E6) Positive Emotions	(O1) Fantasy
Repeat Phase A SAP	Pearson Correlation	.094	.145	-.050
	Sig. (2-tailed)	.811	.710	.899
	N	9	9	9
Repeat Host Defense MDE	Pearson Correlation	.076	-.386	-.416
	Sig. (2-tailed)	.845	.305	.265
	N	9	9	9
Repeat Hematology MDE	Pearson Correlation	.069	.467	.418
	Sig. (2-tailed)	.860	.205	.263
	N	9	9	9
Repeat Neurology MDE	Pearson Correlation	-.034	.295	.331
	Sig. (2-tailed)	.931	.441	.385
	N	9	9	9
Repeat Brain & Behavior MDE	Pearson Correlation	.393	.299	-.116
	Sig. (2-tailed)	.296	.434	.766
	N	9	9	9
Repeat Musculoskeletal MDE	Pearson Correlation	-.266	.161	-.486
	Sig. (2-tailed)	.490	.679	.184
	N	9	9	9

Correlations

		(O2) Aesthetics	(O3) Feelings	(O4) Actions
Repeat Phase A SAP	Pearson Correlation	-.010	-.220	.527
	Sig. (2-tailed)	.980	.570	.145
	N	9	9	9
Repeat Host Defense MDE	Pearson Correlation	-.710*	-.686*	-.384
	Sig. (2-tailed)	.032	.041	.307
	N	9	9	9
Repeat Hematology MDE	Pearson Correlation	.412	.218	.669*
	Sig. (2-tailed)	.271	.574	.049
	N	9	9	9
Repeat Neurology MDE	Pearson Correlation	.682*	.637	.437
	Sig. (2-tailed)	.043	.065	.240
	N	9	9	9
Repeat Brain & Behavior MDE	Pearson Correlation	.176	-.291	.025
	Sig. (2-tailed)	.652	.447	.950
	N	9	9	9
Repeat Musculoskeletal MDE	Pearson Correlation	-.410	-.542	-.166
	Sig. (2-tailed)	.273	.132	.669
	N	9	9	9

Correlations

		(O5) Ideas	(O6) Values	(A1) Trust	(A2) Straightforwardness
Repeat Phase A SAP	Pearson Correlation	-.097	-.281	-.207	.188
	Sig. (2-tailed)	.803	.464	.592	.628
	N	9	9	9	9
Repeat Host Defense MDE	Pearson Correlation	-.533	-.544	.113	-.100
	Sig. (2-tailed)	.140	.130	.773	.798
	N	9	9	9	9
Repeat Hematology MDE	Pearson Correlation	.313	.518	.169	.371
	Sig. (2-tailed)	.412	.153	.664	.325
	N	9	9	9	9
Repeat Neurology MDE	Pearson Correlation	.458	.559	-.038	-.053
	Sig. (2-tailed)	.215	.118	.922	.892
	N	9	9	9	9
Repeat Brain & Behavior MDE	Pearson Correlation	.307	-.290	.351	.651
	Sig. (2-tailed)	.421	.448	.355	.057
	N	9	9	9	9
Repeat Musculoskeletal MDE	Pearson Correlation	-.250	-.195	.468	.320
	Sig. (2-tailed)	.517	.615	.203	.401
	N	9	9	9	9

Correlations

		(A3) Altruism	(A4) Compliance	(A5) Modesty
Repeat Phase A SAP	Pearson Correlation	-.016	-.042	-.253
	Sig. (2-tailed)	.968	.914	.511
	N	9	9	9
Repeat Host Defense MDE	Pearson Correlation	.176	.133	-.217
	Sig. (2-tailed)	.651	.732	.575
	N	9	9	9
Repeat Hematology MDE	Pearson Correlation	.138	.374	.036
	Sig. (2-tailed)	.723	.321	.926
	N	9	9	9
Repeat Neurology MDE	Pearson Correlation	-.250	-.139	-.056
	Sig. (2-tailed)	.517	.722	.887
	N	9	9	9
Repeat Brain & Behavior MDE	Pearson Correlation	.467	.239	.019
	Sig. (2-tailed)	.205	.536	.960
	N	9	9	9
Repeat Musculoskeletal MDE	Pearson Correlation	.670*	.530	.147
	Sig. (2-tailed)	.049	.142	.705
	N	9	9	9

Correlations

		(A6) Tender-Mindedness	(C1) Competence	(C2) Order
Repeat Phase A SAP	Pearson Correlation	.474	.256	.446
	Sig. (2-tailed)	.198	.506	.229
	N	9	9	9
Repeat Host Defense MDE	Pearson Correlation	.061	.060	.313
	Sig. (2-tailed)	.876	.879	.412
	N	9	9	9
Repeat Hematology MDE	Pearson Correlation	-.151	-.310	-.482
	Sig. (2-tailed)	.699	.417	.189
	N	9	9	9
Repeat Neurology MDE	Pearson Correlation	-.106	-.113	-.231
	Sig. (2-tailed)	.786	.771	.549
	N	9	9	9
Repeat Brain & Behavior MDE	Pearson Correlation	.611	.761*	.450
	Sig. (2-tailed)	.080	.017	.224
	N	9	9	9
Repeat Musculoskeletal MDE	Pearson Correlation	.529	.085	-.112
	Sig. (2-tailed)	.143	.828	.775
	N	9	9	9

Correlations

		(C3) Dutifulness	(C4) Achievement Striving	(C5) Self-Discipline
Repeat Phase A SAP	Pearson Correlation	.201	.357	.322
	Sig. (2-tailed)	.605	.345	.397
	N	9	9	9
Repeat Host Defense MDE	Pearson Correlation	-.031	-.178	-.289
	Sig. (2-tailed)	.936	.648	.450
	N	9	9	9
Repeat Hematology MDE	Pearson Correlation	-.171	-.353	-.289
	Sig. (2-tailed)	.660	.351	.450
	N	9	9	9
Repeat Neurology MDE	Pearson Correlation	-.192	.208	.331
	Sig. (2-tailed)	.621	.591	.385
	N	9	9	9
Repeat Brain & Behavior MDE	Pearson Correlation	.776*	.545	.510
	Sig. (2-tailed)	.014	.129	.161
	N	9	9	9
Repeat Musculoskeletal MDE	Pearson Correlation	.509	.272	.194
	Sig. (2-tailed)	.161	.479	.617
	N	9	9	9

Correlations

		(C6) Deliberation	MCAT Verbal	MCAT Biological Sciences
Repeat Phase A SAP	Pearson Correlation	-.237	.208	-.573
	Sig. (2-tailed)	.540	.591	.107
	N	9	9	9
Repeat Host Defense MDE	Pearson Correlation	-.009	-.378	-.561
	Sig. (2-tailed)	.981	.315	.116
	N	9	9	9
Repeat Hematology MDE	Pearson Correlation	-.155	-.009	.560
	Sig. (2-tailed)	.691	.982	.117
	N	9	9	9
Repeat Neurology MDE	Pearson Correlation	-.332	.385	.387
	Sig. (2-tailed)	.382	.306	.304
	N	9	9	9
Repeat Brain & Behavior MDE	Pearson Correlation	.614	-.086	-.155
	Sig. (2-tailed)	.078	.827	.690
	N	9	9	9
Repeat Musculoskeletal MDE	Pearson Correlation	.523	-.834**	-.278
	Sig. (2-tailed)	.149	.005	.469
	N	9	9	9

Correlations

		MCAT Physical Sciences	Yr 1 Phase A MDE	Yr 1 Phase B MDE
Repeat Phase A SAP	Pearson Correlation	.018	.149	-.091
	Sig. (2-tailed)	.962	.703	.816
	N	9	9	9
Repeat Host Defense MDE	Pearson Correlation	-.436	.184	-.283
	Sig. (2-tailed)	.241	.635	.461
	N	9	9	9
Repeat Hematology MDE	Pearson Correlation	.605	.184	-.018
	Sig. (2-tailed)	.085	.635	.962
	N	9	9	9
Repeat Neurology MDE	Pearson Correlation	.433	-.211	.055
	Sig. (2-tailed)	.244	.585	.888
	N	9	9	9
Repeat Brain & Behavior MDE	Pearson Correlation	-.468	.215	.607
	Sig. (2-tailed)	.204	.579	.083
	N	9	9	9
Repeat Musculoskeletal MDE	Pearson Correlation	-.070	-.068	.156
	Sig. (2-tailed)	.859	.862	.689
	N	9	9	9

Correlations

		Yr 1 Host Defense MDE	Yr 2 GI MDE	Yr 2 Renal MDE
Repeat Phase A SAP	Pearson Correlation	.328	.[c]	.[c]
	Sig. (2-tailed)	.389	.	.
	N	9	1	1
Repeat Host Defense MDE	Pearson Correlation	.563	.[c]	.[c]
	Sig. (2-tailed)	.115	.	.
	N	9	1	1
Repeat Hematology MDE	Pearson Correlation	-.159	.[c]	.[c]
	Sig. (2-tailed)	.683	.	.
	N	9	1	1
Repeat Neurology MDE	Pearson Correlation	-.606	.[c]	.[c]
	Sig. (2-tailed)	.084	.	.
	N	9	1	1
Repeat Brain & Behavior MDE	Pearson Correlation	.518	.[c]	.[c]
	Sig. (2-tailed)	.153	.	.
	N	9	1	1
Repeat Musculoskeletal MDE	Pearson Correlation	.004	.[c]	.[c]
	Sig. (2-tailed)	.992	.	.
	N	9	1	1

Correlations

		Yr 2 Infectious Diseases MDE	Yr 1 Hematology MDE	Yr 1 Neurology MDE
Repeat Phase A SAP	Pearson Correlation	.[c]	.645	.[c]
	Sig. (2-tailed)	.	.061	.
	N	0	9	1
Repeat Host Defense MDE	Pearson Correlation	.[c]	.415	.[c]
	Sig. (2-tailed)	.	.266	.
	N	0	9	1
Repeat Hematology MDE	Pearson Correlation	.[c]	-.173	.[c]
	Sig. (2-tailed)	.	.656	.
	N	0	9	1
Repeat Neurology MDE	Pearson Correlation	.[c]	-.352	.[c]
	Sig. (2-tailed)	.	.353	.
	N	0	9	1
Repeat Brain & Behavior MDE	Pearson Correlation	.[c]	.521	.[c]
	Sig. (2-tailed)	.	.150	.
	N	0	9	1
Repeat Musculoskeletal MDE	Pearson Correlation	.[c]	.055	.[c]
	Sig. (2-tailed)	.	.888	.
	N	0	9	1

Correlations

		Yr 1 Brain & Behavior MDE	Yr 1 Musculoskeletal MDE	Yr 1 Community Epidimiology Study Grade
Repeat Phase A SAP	Pearson Correlation	-.499	.[c]	.019
	Sig. (2-tailed)	.392	.	.961
	N	5	1	9
Repeat Host Defense MDE	Pearson Correlation	.259	.[c]	-.061
	Sig. (2-tailed)	.674	.	.877
	N	5	1	9
Repeat Hematology MDE	Pearson Correlation	.285	.[c]	-.308
	Sig. (2-tailed)	.642	.	.420
	N	5	1	9
Repeat Neurology MDE	Pearson Correlation	-.477	.[c]	-.091
	Sig. (2-tailed)	.417	.	.816
	N	5	1	9
Repeat Brain & Behavior MDE	Pearson Correlation	.652	.[c]	.351
	Sig. (2-tailed)	.233	.	.355
	N	5	1	9
Repeat Musculoskeletal MDE	Pearson Correlation	-.075	.[c]	-.616
	Sig. (2-tailed)	.905	.	.078
	N	5	1	9

Correlations

		Yr 2 Cardiology MDE	Yr 2 Biostatistics	Yr 2 Pulmonology MDE
Repeat Phase A SAP	Pearson Correlation	.[c]	.[c]	.[c]
	Sig. (2-tailed)	.	.	.
	N	1	1	1
Repeat Host Defense MDE	Pearson Correlation	.[c]	.[c]	.[c]
	Sig. (2-tailed)	.	.	.
	N	1	1	1
Repeat Hematology MDE	Pearson Correlation	.[c]	.[c]	.[c]
	Sig. (2-tailed)	.	.	.
	N	1	1	1
Repeat Neurology MDE	Pearson Correlation	.[c]	.[c]	.[c]
	Sig. (2-tailed)	.	.	.
	N	1	1	1
Repeat Brain & Behavior MDE	Pearson Correlation	.[c]	.[c]	.[c]
	Sig. (2-tailed)	.	.	.
	N	1	1	1
Repeat Musculoskeletal MDE	Pearson Correlation	.[c]	.[c]	.[c]
	Sig. (2-tailed)	.	.	.
	N	1	1	1

Correlations

		Yr 2 Endocrinology MDE	Yr 2 Research Design	Repeat Phase A MDE
Repeat Phase A SAP	Pearson Correlation	.[c]	.[c]	.305
	Sig. (2-tailed)	.	.	.425
	N	1	1	9
Repeat Host Defense MDE	Pearson Correlation	.[c]	.[c]	.767*
	Sig. (2-tailed)	.	.	.016
	N	1	1	9
Repeat Hematology MDE	Pearson Correlation	.[c]	.[c]	-.210
	Sig. (2-tailed)	.	.	.588
	N	1	1	9
Repeat Neurology MDE	Pearson Correlation	.[c]	.[c]	-.625
	Sig. (2-tailed)	.	.	.072
	N	1	1	9
Repeat Brain & Behavior MDE	Pearson Correlation	.[c]	.[c]	-.137
	Sig. (2-tailed)	.	.	.725
	N	1	1	9
Repeat Musculoskeletal MDE	Pearson Correlation	.[c]	.[c]	.100
	Sig. (2-tailed)	.	.	.797
	N	1	1	9

Correlations

		Repeat Phase B MDE	Repeat Phase A SAP	Repeat Host Defense MDE
Repeat Phase A SAP	Pearson Correlation	.193	1	-.025
	Sig. (2-tailed)	.620		.950
	N	9	9	9
Repeat Host Defense MDE	Pearson Correlation	.102	-.025	1
	Sig. (2-tailed)	.793	.950	
	N	9	9	9
Repeat Hematology MDE	Pearson Correlation	.190	-.174	-.481
	Sig. (2-tailed)	.625	.655	.190
	N	9	9	9
Repeat Neurology MDE	Pearson Correlation	.039	.091	-.883**
	Sig. (2-tailed)	.921	.816	.002
	N	9	9	9
Repeat Brain & Behavior MDE	Pearson Correlation	.103	.261	-.008
	Sig. (2-tailed)	.793	.498	.983
	N	9	9	9
Repeat Musculoskeletal MDE	Pearson Correlation	-.453	-.300	.334
	Sig. (2-tailed)	.220	.433	.380
	N	9	9	9

Correlations

		Repeat Hematology MDE	Repeat Neurology MDE	Repeat Brain & Behavior MDE
Repeat Phase A SAP	Pearson Correlation	-.174	.091	.261
	Sig. (2-tailed)	.655	.816	.498
	N	9	9	9
Repeat Host Defense MDE	Pearson Correlation	-.481	-.883**	-.008
	Sig. (2-tailed)	.190	.002	.983
	N	9	9	9
Repeat Hematology MDE	Pearson Correlation	1	.430	-.144
	Sig. (2-tailed)		.248	.713
	N	9	9	9
Repeat Neurology MDE	Pearson Correlation	.430	1	-.315
	Sig. (2-tailed)	.248		.410
	N	9	9	9
Repeat Brain & Behavior MDE	Pearson Correlation	-.144	-.315	1
	Sig. (2-tailed)	.713	.410	
	N	9	9	9
Repeat Musculoskeletal MDE	Pearson Correlation	-.234	-.265	.055
	Sig. (2-tailed)	.545	.490	.889
	N	9	9	9

Correlations

		Repeat Musculoskeletal MDE
Repeat Phase A SAP	Pearson Correlation	-.300
	Sig. (2-tailed)	.433
	N	9
Repeat Host Defense MDE	Pearson Correlation	.334
	Sig. (2-tailed)	.380
	N	9
Repeat Hematology MDE	Pearson Correlation	-.234
	Sig. (2-tailed)	.545
	N	9
Repeat Neurology MDE	Pearson Correlation	-.265
	Sig. (2-tailed)	.490
	N	9
Repeat Brain & Behavior MDE	Pearson Correlation	.055
	Sig. (2-tailed)	.889
	N	9
Repeat Musculoskeletal MDE	Pearson Correlation	1
	Sig. (2-tailed)	
	N	9

*. Correlation is significant at the 0.05 level (2-tailed).

**. Correlation is significant at the 0.01 level (2-tailed).

c. Cannot be computed because at least one of the variables is constant.
